# Spleen‐targeted neoantigen DNA vaccine for personalized immunotherapy of hepatocellular carcinoma

**DOI:** 10.15252/emmm.202216836

**Published:** 2023-08-08

**Authors:** Ming Wu, Zijin Luo, Zhixiong Cai, Qianqian Mao, Zhenli Li, Hao Li, Cao Zhang, Yuting Zhang, Aoxue Zhong, Liming Wu, Xiaolong Liu

**Affiliations:** ^1^ The United Innovation of Mengchao Hepatobiliary Technology Key Laboratory of Fujian Province Mengchao Hepatobiliary Hospital of Fujian Medical University Fuzhou China; ^2^ The Liver Center of Fujian Province Fujian Medical University Fuzhou China; ^3^ Mengchao Med‐X Center Fuzhou University Fuzhou China; ^4^ Department of Hepatobiliary and Pancreatic Surgery, the First Affiliated Hospital, Zhejiang Provincial Key Laboratory of Pancreatic Disease, School of Medicine Zhejiang University Hangzhou China

**Keywords:** hepatocellular carcinoma, neoantigen DNA vaccine, personalized immunotherapy, RBC hitchhiking, spleen targeting, Cancer, Immunology

## Abstract

Neoantigens are emerging as attractive targets to develop personalized cancer vaccines, but their immunization efficacy is severely hampered by their restricted accessibility to lymphoid tissues where immune responses are initiated. Leveraging the capability of red blood cells (RBCs) to capture and present pathogens in peripheral blood to the antigen‐presenting cells (APCs) in spleen, we developed a RBC‐driven spleen targeting strategy to deliver DNA vaccine encoding hepatocellular carcinoma (HCC) neoantigen. The DNA vaccine‐encapsulating polymeric nanoparticles that were intentionally hitchhiked on the preisolated RBCs could preferentially accumulate in the spleen to promote the neoantigen expression by APCs, resulting in the burst of neoantigen‐specific T‐cell immunity to prevent tumorigenesis in a personalized manner, and slow down tumor growth in the established aggressively growing HCC. Remarkably, when combined with anti‐PD‐1, the vaccine achieved complete tumor regression and generated a robust systemic immune response with long‐term tumor‐specific immunological memory, which thoroughly prevented tumor recurrence and spontaneous lung metastasis. This study offers a prospective strategy to develop personalized neoantigen vaccines for augmenting cancer immunotherapy efficiency in immune “cold” HCC.

## Graphical abstract

To address the limited efficacy of current personalized cancer vaccines and improve hepatocellular carcinoma (HCC) immunotherapy, a red blood cell (RBC) hitchhiking strategy was developed for targeted delivery of a neoantigen DNA vaccine to the spleen.

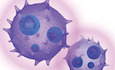

**The paper explained**

**Problem**

Neoantigens hold great promise for the development of personalized cancer vaccines to achieve targeted immunotherapy. However, hepatocellular carcinoma (HCC) often exhibits a highly immune “cold” microenvironment with a low to moderate tumor mutational burden (TMB), limiting the efficacy of such vaccines. In this study, we developed a red blood cell (RBC)‐hitchhiking strategy to deliver the neoantigen DNA vaccine for targeted HCC immunotherapy, aiming to drive personalized antitumor immunity for HCC.

**Results**

The DNA vaccine‐encapsulating polymeric nanoparticles were deliberately hitchhiked on the preisolated red blood cells (RBCs) to facilitate their preferential accumulation in the spleen after systemic administration. This led to enhanced expression of neoantigens by antigen‐presenting cells (APCs), triggering a robust neoantigen‐specific T‐cell immune response. As a result, personalized prevention of tumorigenesis and suppression of tumor growth were achieved. Notably, when combined with anti‐PD‐1 checkpoint blockade, this vaccination approach resulted in complete tumor regression in 75% of mice with subcutaneous HCC and 100% of mice with orthotopic HCC, leading to significantly prolonged survival. Furthermore, the cured mice exhibited a strong systemic immune response and long‐term tumor‐specific immunological memory, effectively preventing tumor recurrence and spontaneous lung metastasis.

**Impact**

This study presents an efficient immunization strategy by using the RBC‐hitchhiking neoantigen DNA nanovaccine for personalized HCC treatment, which provides valuable insights for designing novel and effective therapeutic vaccines to against refractory solid tumors.

## Introduction

Personalized neoantigen cancer vaccines (NCVs), driven by the next‐generation sequencing and innovative bioinformatics to identify nonsynonymous genome alterations of cancer cells, have gradually emerged as a promising therapeutic approach to fight against malignancy with merits of exquisite specificity, high immunogenicity, and lack of central tolerance (Hu *et al*, 2018). Since the first‐in‐human trail of dendritic cell (DC)‐based NCVs on three patients with advanced melanoma in 2015 (Carreno *et al*, 2015), such an approach has been extensively applied in multiple malignancies, mainly including melanoma and non‐small‐cell lung cancer (NSCLC) that are characterized by well‐established immunogenicity and high tumor mutation burden (TMB) of more than 20 somatic mutations per megabase (Mut/Mb; Schumacher & Schreiber, 2015; Sahin *et al*, 2017). In contrast, the HCC bearing a highly immune “cold” microenvironment with a low‐to‐moderate TMB (median number of 5 Mut/Mb), as well as the limited, exhausted, or dysfunctional tumor‐infiltrating lymphocytes (TILs), generally responds poorly to various immunotherapies with an overall response rate (ORR) of less than 20% (Kelley, 2020; Sangro *et al*, 2021). To date, the early‐phase clinical trials of NCVs in HCC are fairly rare, and no impressive results have yet been reported despite being demonstrated to be safe and have certain immunologic outcomes (http://www.clinicaltrials.gov). To increase their antitumor activity, our group recently has screened a pool of neoantigen peptides that can be presented on MHC class I or II of APCs and then specifically recognized by T cells to elicit antitumor immunity in animal models and patients with HCC, through optimizing the identification pipeline of neoepitopes (Zeng *et al*, 2020; Cai *et al*, 2021; Zhao *et al*, 2022b). Although the “quality” of the neoantigens is important, the options of formulations and delivery vehicles also largely determine the immunoactivity extent of cancer vaccines (Kuai *et al*, 2017; Zhang *et al*, 2021a).

NCVs can be delivered in the formats of peptide, DNA, RNA, and even dendritic cells (DCs), etc. Compared with other formulations, DNA‐based NCVs can provide a long‐term antigen‐specific immune response once APCs are successfully transfected by a plasmid DNA encoding neoantigens (Rice *et al*, 2008; Nguyen *et al*, 2020). Additionally, DNA vaccines have advantages of molecular flexibility, thermal stability, and relative ease of manufacture (Li *et al*, 2021). However, direct immunization of DNA‐based vaccines has faced several obstacles. The large size, highly negative charge, and susceptibility to degradation of DNA result in low cellular uptake, bioavailability, and transfection, which ultimately lead to minimal immunogenicity of DNA‐based vaccines (Nguyen *et al*, 2020). These limitations can be overcome through development of nanoscaled delivery vehicles to effectively protect and transport DNA into cells. Nevertheless, conventional delivery of DNA‐based vaccines via intramuscular, intradermal, or subcutaneous administration routes still suffers from limited availability, as they are mostly taken up by myocytes or keratinocytes rather than professional APCs such as dendritic cells (DCs; Zhang *et al*, 2021a; Wang *et al*, 2022; Zhao *et al*, 2022a). How to achieve the best spatiotemporal orchestration, distinct tissue compartmental distribution, and diverse cell type accumulation of nanoscaled vaccines for efficiently presenting neoantigens on professional APCs and cross‐priming neoantigen‐specific T cells remains to be resolved.

Spleen, as the biggest peripheral lymphoid organ containing abundant immune cells such as APCs and T cells, is the ideal place for activating DCs and priming neoantigen‐specific T‐cell response (Zhai *et al*, 2021). Therefore, targeted delivery of neoantigens to the spleen can provoke an efficient and systemic antitumor immunity. Considerable efforts aimed at spleen‐targeted delivery have been largely resorted to modifying nanoparticles with affinity ligands (Kedmi *et al*, 2018). Besides, growing evidences have demonstrated that the biodistribution of lipoplex‐based nanovehicles can be shifted from liver to spleen by adjusting the surface charge from positive or neutral to negative after systematic administration (Kranz *et al*, 2016; Cheng *et al*, 2020). Nevertheless, negative charge will become a roadblock with respect to endocytosis into APCs for neoantigen transfection and neoepitope presentation. Biomimicry could provide a choice to simultaneously satisfy the high organ targeting and specific cell transfection for DNA‐based vaccines. Very recently, Mitragotri group has developed a series of “RBC‐hitchhiking” delivery strategies to address the challenges faced by nanomedicine for site‐specific delivery of a range of payloads, including small molecules, peptides, proteins, and antibodies (Brenner *et al*, 2018; Zhao *et al*, 2019, 2021). Notably, the innate and unique ability of RBCs to capture and present pathogens on their surface to the professional APCs in spleen (in terms of “blood filter” role) could also be leveraged to engineer an efficient vehicle handoff for spleen targeting without specific modifications (Ukidve *et al*, 2020). This provides an amazing opportunity to effectively targeted delivery of the DNA vaccine into spleen by the elaborately tailored design of RBC‐based strategies to boost a strong anticancer immune response.

**Figure EV1:**
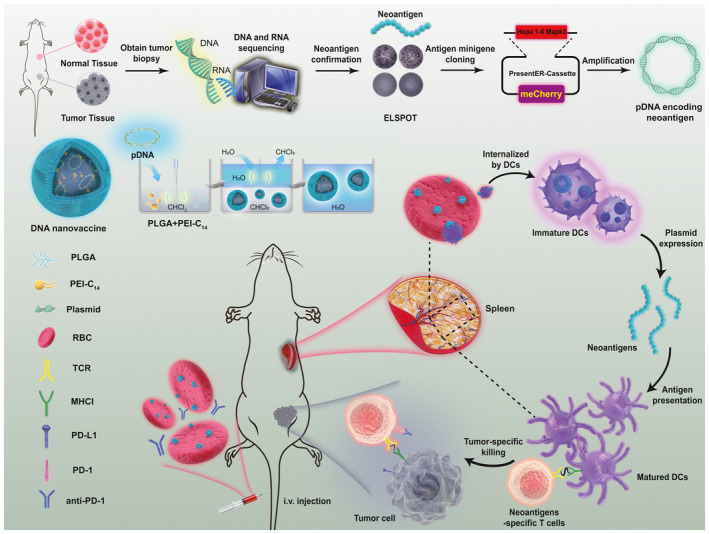
Schematic illustration of the highly efficient DNA neoantigen vaccine by utilizing a cascade delivery strategy to drive personalized immunotherapy against HCC The Hepa 1‐6 tumor‐specific neoantigens were firstly screened by transcriptome sequencing and whole‐exome sequencing, and the pDNA encoding neoantigens were constructed by using “PresentER” cassette. Subsequently, the DNA nanovaccines were synthesized by the double microemulsion method (W/O/W) with PEI_25000_‐C_14_ and PLGA, and then attached on the surface of the preisolated RBCs by the electrostatic adsorption to form a RBC‐hitchhiking DNA nanovaccine (RBC‐Nanovaccine). After systemic delivery, RBC‐Nanovaccines are accumulated into the body's biggest lymphoid organ (spleen) by leveraging the innate “blood filtration” function of RBCs, then dislodging them from the RBCs to antigen‐presenting cells (APCs) to promote the neoantigen production and cross‐presentation. Finally, antigen‐specific immune responses were activated, which prevented tumorigenesis and inhibited tumor growth and recurrence especially in combination with anti‐PD‐1 antibody, as evidenced in different models of Hepa 1‐6 tumor‐bearing mice.

Here, we developed a highly efficient DNA neoantigen vaccine by utilizing a cascade delivery strategy to improve its bioavailability in the spleen to drive personalized immunotherapy against HCC (Fig EV1). Specifically, we firstly engineered a polymer–lipid nanoparticle (NP) that encapsulated the plasmid DNA (pDNA, carrying neoantigen transgene expression cassette) by self‐assembly with the tetradecane‐graft polyethyleneimine (PEI_25000_‐C_14_) and PLGA, and these preformed pDNA‐NPs (termed as nanovaccines) were then attached to the surface of preisolated RBCs for relayed delivery of DNA vaccine. Purposefully, the RBCs serve as the primary delivery vehicle to selectively dislodge nanovaccines into spleen by leveraging its “blood filter” role (Lloyd & Strickland, 2010), while polymer–lipid nanoparticles as the secondary delivery vehicle can protect pDNA from rapid degradation and facilitate its traverse across APC membrane for neoantigen expression (Krohn‐Grimberghe *et al*, 2020). Using this cascade strategy to deliver neoantigen screened from murine HCC cell line (Hepa 1‐6) through our well‐established protocol, we successfully developed and optimized a prospective DNA vaccine to prevent tumorigenesis as well as inhibit tumor growth and recurrence/metastasis, especially combined with anti‐PD‐1 antibody to remodel immune “cold” microenvironment in murine HCC model, which offers a personalized, potent, and translatable immunization paradigm for future HCC treatment.

## Results

### Synthesis and characterization of HCC neoantigen‐based DNA nanovaccine

**Figure 1:**
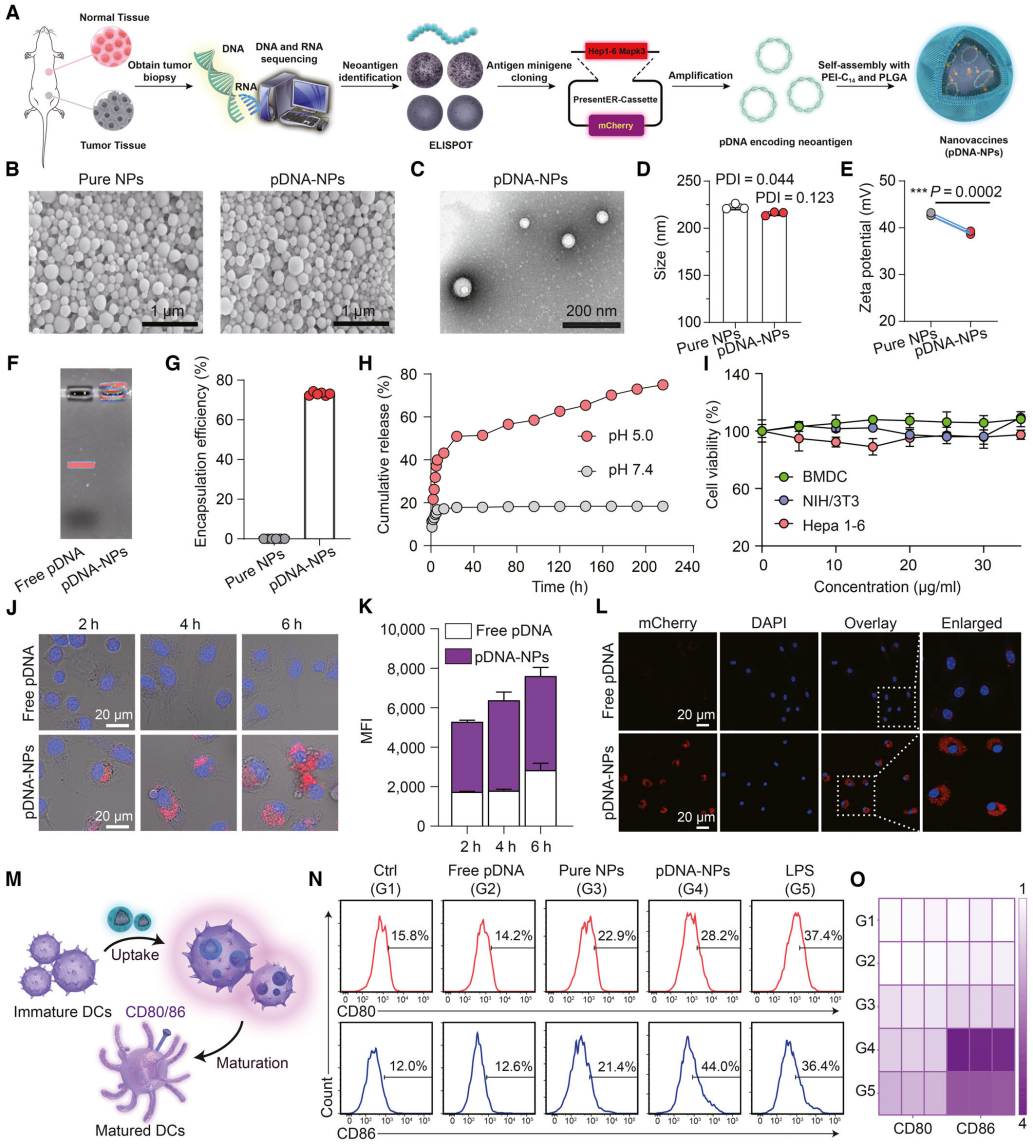
Preparation of DNA nanovaccines for neoantigen delivery A
Schematic illustration of the construction of DNA nanovaccines (pDNA‐NPs) encoding Hepa 1‐6 liver cancer cell‐specific neoantigen.B
SEM images of pure NPs and pDNA‐NPs.C
TEM image of pDNA‐NPs.D–F
Average size (D), zeta potential (E), and agarose gel electrophoresis (F) of pure NPs and pDNA‐NPs (*n* = 3 biological replicates per group).G
The encapsulation efficiency and content of pDNA‐NPs, and pure NPs were taken as benchmark (*n* = 6 biological replicates per group).H
The release kinetics of pDNA from pDNA‐NPs in acidic (pH 5.0) and neutral (pH 7.4) conditions (*n* = 3 biological replicates per group).I
Cell viability of BMDCs, NIH/3T3, and Hepa 1‐6 cells incubated with different concentrations of pDNA‐NPs (*n* = 5 biological replicates per group). The shadow indicated the errors.J, K
CLSM images (J) and flow cytometry detecting the mean fluorescence intensity (MFI) (K) of BMDCs treated with free pDNA and pDNA‐NPs for 2, 4, and 6 h, respectively. Nucleus was stained with DAPI (blue) and pDNA was labeled with YOYO‐3 (red) in (J). *n* = 3 biological replicates per group in (K).L
CLSM images of mCherry expression in BMDCs transfected with free pDNA and pDNA‐NPs for 48 h.M
Schematic illustration of the maturation of BMDCs stimulated by different formulations.N, O
The expression of CD80 and CD86 after BMDCs co‐incubated with free pDNA, pure NPs, and pDNA‐NPs for 24 h, respectively. LPS was used as the positive control. Data information: Data in (D), (E), (G), (H), (I), and (K) are mean ± SD. Statistical analyses were conducted by two‐tailed Student's *t*‐test in (E); ***P < 0.001. Note: For the cellular uptake experiments, we used DNA without mCherry tag but stained with YOYO‐3. For transfection validation, we used mCherry‐tagged DNA without YOYO‐3 staining. However, neither of these fluorescent dyes was used in the subsequent functional experiments. Source data are available online for this figure.

In our previous study, a Hepa 1‐6 tumor cell‐specific neoantigen (MKARNYLQSLPSKTKVA → MKARNYLQFLPSKTKVA) with high affinity to MHC I H‐2K^b^ (C57BL/6 mice) was identified based on whole‐exome sequencing and transcriptomic sequencing of murine HCC cell line (Hepa 1‐6 cells) and C57BL/6 mouse tail tissue using our proprietary bioinformatics strategies, and further validated with strong antitumor immunogenicity *in vivo* (Chen *et al*, 2022). Afterward, the sequence of neoantigen peptide was engineered into a DNA plasmid (pDNA) by using a “PresentER” cassette comprised of endoplasmic reticulum (ER) signal sequence followed by a short peptide/epitope, which enabled readily display of neoantigen epitope on APC cells according to previously reported methods (Fig 1A; Gejman *et al*, 2018). Of note, the pDNA contains a mCherry tag in “PresentER” cassette to monitor neoantigen expression. The successful construction and feasibility of pDNA as a neoantigen resource were demonstrated by the sequencing results and mCherry expression in 293FT cells (Appendix Fig S1). Next, to facilitate pDNA encapsulation and cytosolic transport, we synthesized a lipid‐like cationic polymer PEI_25000_‐C_14_ (Appendix Fig S2) through the ring opening of 1,2‐epoxytetradecane by branched PEI (Mw ~ 25,000), which can reduce the cytotoxicity of PEI and improve its transfection efficiency (Krohn‐Grimberghe *et al*, 2020). PEI_25000_‐C_14_, PLGA, and pDNA were then self‐assembled into nanovaccines by a double emulsion method, in which PEI_25000_‐C_14_ was used for pDNA complexation via electrostatic interactions, and PLGA (a widely clinically used biodegradable and biocompatible polymer) was served as a stable container for PEI_25000_‐C_14_/pDNA complex (Islam *et al*, 2018). The DNA nanovaccines (pDNA‐NPs) displayed a uniform and spherical morphology with an average diameter of 100 nm (Fig 1B and C), as characterized by scanning electron microscope (SEM) and transmission electron microscope (TEM). In addition, dynamic light scattering (DLS) analysis indicated that the hydrodynamic diameter of DNA nanovaccines (pDNA‐NPs) was 215.7 ± 2.1 nm, which was almost the same as that of pure NPs without loading of pDNA (222.7 ± 3.1 nm; Fig 1D and Appendix Fig S3), indicating that the encapsulation of pDNA did not affect the polydispersity of nanoparticles. Meanwhile, the zeta potential of DNA nanovaccines (pDNA‐NPs) slightly decreased from 42.8 ± 0.38 to 38.9 ± 0.36 mV due to encapsulation of negatively charged pDNA (Fig 1E). Such a positively charged feature can be helpful to attach the nanovaccines on RBCs and deliver the pDNA into APCs through electrostatic interaction with the negatively charged cell membrane. To further confirm the encapsulation of pDNA, it was prelabeled with YOYO‐3 for constructing DNA nanovaccine and then analyzed by agarose gel electrophoresis. As shown in Fig 1F, the pDNA in nanovaccine showed no electrophoretic shift in comparison with free pDNA, indicating the effective packaging of pDNA into nanovaccine, with an encapsulation efficiency of 73.07 ± 0.86% (Fig 1G and Appendix Fig S4). The release kinetics of pDNA from nanovaccine was further investigated at 37°C under a mimic physiological condition (pH 7.4) and lysosomal acidic environment (pH 5.0). As shown in Fig 1H, the pDNA release was much faster at pH 5.0 in comparison with that at pH 7.4, with a cumulative release of 51.42 ± 0.72 and 17.90 ± 0.36% within 48 h, respectively. This result indicates that the nanovaccines can prevent pDNA release from the bloodstream while enabling its release from lysosome to cytoplasm for gene transduction.

### DNA nanovaccine with low cytotoxicity and high transfection efficiency in APCs

The *in vitro* cytotoxicity of DNA nanovaccines was evaluated on various cells, including mouse bone marrow‐derived dendritic cells (BMDCs), mouse embryo fibroblast cells (NIH/3T3), and mouse HCC cells (Hepa 1‐6). After incubation with DNA nanovaccines for 24 h, the cell viability was still maintained above 80% even at the highest concentration of 35 μg/ml (Fig 1I). Since effective interaction of our DNA nanovaccine with APC is a prerequisite for neoantigen processing and cross‐presentation, we explored the internalization of YOYO‐3‐labeled DNA nanovaccines in BMDCs using confocal laser scanning microscopy (CLSM) and flow cytometry. The intracellular red signal from YOYO‐3‐labeled pDNA correlated well with incubation time, and the internalization efficacy (in terms of mean MFI detected by flow cytometry) of DNA nanovaccines was much higher than that of free pDNA, verifying that the as‐prepared polymer–lipid nanoparticulated vehicles could efficiently deliver pDNA into APCs (Fig 1J and K, and Appendix Fig S5). As the incubation time increased to 24 h, some of the YOYO‐3 fluorescence signals appeared in the cell nucleus, indicating successful delivery of DNA into the nucleus (Appendix Fig S6). Additionally, we examined the transfection efficiency in BMDCs by CLSM. As expected, the fluorescence signal of mCherry was clearly observed after incubation with DNA nanovaccines for 48 h, which was significantly higher than that of BMDCs incubated with free pDNA (Fig 1L). Maturation of DCs is a distinct feature for antigen presentation to elicit subsequent immune responses (Xu *et al*, 2020). Interestingly, BMDCs treated with pure NPs without pDNA also exhibited an obvious upregulation of co‐stimulatory molecules (CD80 and CD86) compared with the control and free pDNA groups, which is consistent with the previous reports that lipid‐like cationic polymers (PEI_25000_‐C_14_ components in our nanocarriers) could induce dendritic cell maturation through stimulating the Toll‐like receptor 4 (TLR4) signaling pathway to act as adjuvants (Xu *et al*, 2020; Zhang *et al*, 2021b). Most significantly, the assembled DNA nanovaccines induced the highest level of BMDC maturation (Fig 1M–O and Appendix Fig S7). This is because that the DNA vaccine itself can stimulate DC maturation through both the TLR9 and STING pathways (Deb *et al*, 2020), and the nanovaccine can further amplify this effect by enhancing the efficiency of DC uptake. These results indicate that the as‐prepared polymer–lipid hybrid biocompatible nanoparticles with innate immune activity can act as a delivery vehicle to effectively transport pDNA in APCs for their activation, which could contribute to following neoantigen cross‐presentation and specific T‐cell response elicitation.

### Red blood cell hitchhiking of DNA nanovaccines to improve spleen accumulation

**Figure 2:**
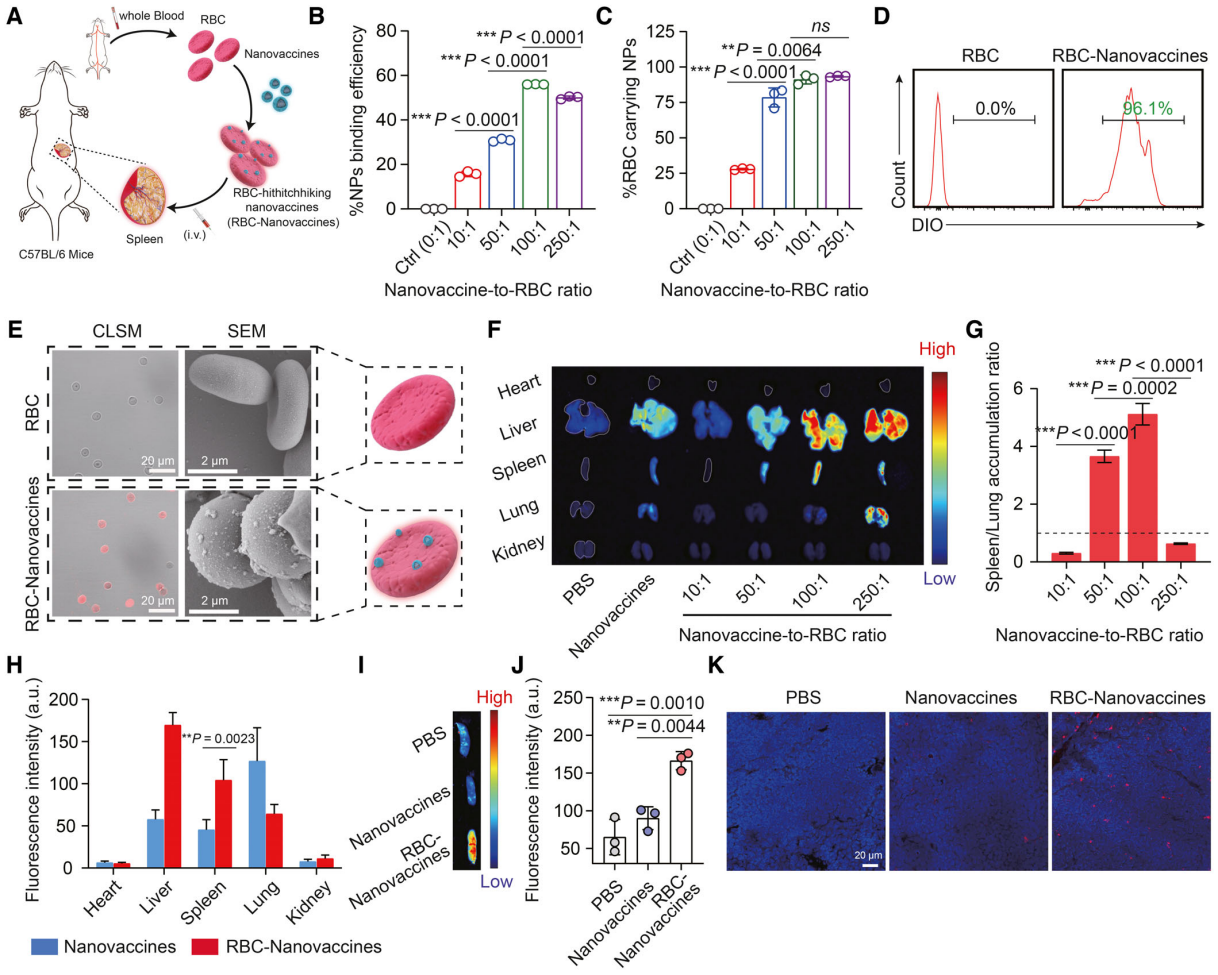
DNA nanovaccines hitchhiked on RBCs to achieve spleen accumulation Schematic illustration of the construction of RBC‐hitchhiking DNA nanovaccines (RBC‐Nanovaccines) for spleen‐targeted delivery.Attaching efficiency of DNA nanovaccines on RBCs with different feed ratios (*n* = 3 biological replicates per group).The percentage of RBC carrying DIO‐labeled DNA nanovaccines at different nanovaccine‐to‐RBC ratios, which is detected by flow cytometry (*n* = 3 biological replicates per group).Representative flow cytometry histograms of pure RBC or RBC attached with DIO‐labeled DNA nanovaccines at the nanovaccine‐to‐RBC ratio of 100:1.CLSM (left) and SEM (right) images of pure RBCs or RBCs attaching with DiI‐labeled DNA nanovaccines at the nanovaccine‐to‐RBC of 100:1.
*Ex vivo* fluorescence images of major organs 24 h after intravenous injection of the DiI‐labeled DNA nanovaccines hitchhiked on RBCs at different feed ratios, and compared to pure DNA nanovaccines.Spleen‐to‐lung fluorescence intensity accumulation ratios calculated from their fluorescence intensity in (F) (*n* = 3 animals per group).Biodistribution of DiI‐labeled pure DNA nanovaccines and RBC‐hitchhiking DNA nanovaccines in major organs, expressed in terms of % injected dose per gram of tissue (*n* = 4–6 animals per group).Fluorescence images of the spleen 48 h after intravenous administration of pure DNA nanovaccines and RBC‐hitchhiking DNA nanovaccines.Analysis of the fluorescence intensity of the spleen in (I) (*n* = 3 animals per group).CLSM images of cryosections of spleen 48 h after intravenous injection of pure DNA nanovaccines and RBC‐hitchhiking DNA nanovaccines (nucleus were stained with DAPI). Data information: Data in (B), (C), (G), (H), and (J) are presented as mean ± SD. Statistical significance was calculated by two‐tailed Student's *t*‐test in (B, C, G, H) and one‐way ANOVA with Tukey's multiple comparison test in (J). *P < 0.05, **P < 0.01, and ***P < 0.001. ns means no significant difference. Source data are available online for this figure.

Before using nanocarriers to improve the transport of DNA vaccines into APC cells, another important prerequisite is that these nanosized vaccines should be effectively delivered to lymphoid tissues/organs (spleen in this case). To further improve the bioavailability of DNA vaccines, we developed a cascade delivery strategy that could deliver DNA nanovaccines into spleen by leveraging the “blood filter” role of red blood cells (RBCs). The intended workflow is to first attach the DNA nanovaccines to RBCs *ex vivo*, followed by intravascular administration to accumulate in spleen (Fig 2A). To investigate the hitchhiking behavior, RBCs extracted from C57BL/6 mice were washed with cold PBS to remove serum and then incubated with DiO‐ or DiI‐labeled DNA nanovaccines at different feed ratios of nanovaccine‐to‐RBC for 1 h. Unexpectedly, the attachment efficiency (expressed as the percentage of nanovaccines attached to RBCs within the feed amount of nanovaccines) quantified by fluorescence measurement was found to be positively dependent on the feed ratio, which reached a maximum of 56.0 ± 0.090% at a nanovaccine‐to‐RBC ratio of 100:1 (Fig 2B and Appendix Fig S8). Meanwhile, the flow cytometry analysis showed that almost all RBCs were hitchhiked with DNA nanovaccines at the nanovaccine‐to‐RBC ratio of 100:1 (Fig 2C and D). Next, we employed a hemolysis assay to evaluate the influence of nanovaccine attachment to RBCs. As shown in Appendix Fig S9, there was an obvious hemolysis phenomenon when the nanovaccine‐to‐RBC ratio increased to 250:1, but with minimal effect below 100:1. The presence of DNA nanovaccines on the surface of the RBC exemplified at nanovaccine‐to‐RBC ratio of 100:1 was further visually confirmed by CLSM and SEM imaging (Fig 2E).

Lung has previously been demonstrated to be the first downstream organ of RBC‐hitchhiking nanoparticles through intravenous (i.v.) injection, where the nanoparticles are easily dislodged from the RBCs owing to the high shear stress in narrow lung capillaries (Brenner *et al*, 2018; Zhao *et al*, 2021). Thus, to assess whether RBC‐hitchhiking DNA nanovaccines can escape from lungs to deliver their cargos elsewhere (spleen in this case), we tested the *in vivo* biodistribution at various feed ratios of nanovaccine‐to‐RBC but injecting with the same number of RBCs. After 24 h of injection, the fluorescence intensities of the harvested organs especially lung and spleen were evaluated (Fig 2F and G). A low nanovaccine‐to‐RBC (10:1) ratio resulted in a low spleen‐to‐lung accumulation ratio (~0.3), while a high nanovaccine‐to‐RBC ratio showed an increased spleen accumulation with the spleen‐to‐lung ratio of 3.6 and 5.1 at nanovaccine‐to‐RBC ratio of 50:1 and 100:1, respectively. However, as the feed ratio of nanovaccine‐to‐RBC further increased to 250:1, the spleen‐to‐lung ratio decreased again to 0.6, likely ascribing to the aggregation of RBCs at higher nanovaccine‐to‐RBC ratio to clog into the pulmonary vessels (Brenner *et al*, 2018). To further clarify the relevant mechanisms of organ tropism profiles, we compared shear resistance of RBC‐hitchhiking nanovaccines at the nanovaccine‐to‐RBC ratios of 10:1 and 100:1 at a low (~1 Pa) or high (6 Pa) shear stress. As shown in Appendix Fig S10, detachment of the nanovaccine cargos from RBCs was found to be dependent on shear stress and nanovaccine‐to‐RBC ratio. Obviously, above 75% of the hitchhiked DNA nanovaccines at loading ratio of 10:1 came off at the lung capillaries‐related shear stress (6 Pa), but exhibited stronger shear resistance with loading ratio increased to 100:1, possibly because of the stiffening of RBCs with high nanovaccine loads (Ukidve *et al*, 2020). This result indicated that a substantial proportion of DNA nanovaccines hitchhiking on RBCs could successfully pass through lung capillaries and be captured in spleen at high nanovaccine‐to‐RBC ratios. Notably, the nanovaccine‐to‐RBC ratio at 100:1 achieved the best enrichment in spleen, and thus, RBC‐hitchhiking nanovaccines at this ratio were selected for following studies. Meanwhile, their biodistribution at the ratio of 100:1 was compared with that of equivalent amount of nanovaccines. As shown in Fig 2H and Appendix Fig S11, the nanovaccines were easily cleared out from mice with much less accumulation in spleen in comparison with those hitchhiking on RBCs, highlighting the superiority of RBC to deliver DNA nanovaccines into spleen. Additionally, we included a comparison group in which the nanovaccines were coated with RBC membrane (Nanovaccine@RBCM) instead of being adsorbed on RBCs. However, due to the strong positive charge of the nanovaccine itself, we observed the formation of aggregates when mixed with negatively charged red blood cell membranes. These aggregates led to significant deposition in the lungs while limited accumulation in the spleen upon intravenous administration (Appendix Fig S11).

To further confirm the internalization of nano‐packaged DNA by APC cells in the spleen, the DiI‐labeled RBC‐Nanovaccines were intravenously injected into C57BL/6 mice. After 24 h of injection, the percentage of DiI^+^CD11c^+^ DCs isolated from spleen was analyzed by flow cytometry. As depicted in Appendix Fig S12, mice treated with RBC‐Nanovaccines demonstrated significantly higher uptake efficacy in DCs, with a dual positive ratio of 9.83 ± 1.86%, compared to those treated with pure nanovaccine (4.45 ± 0.84%) or Nanovaccine@RBCM (4.12 ± 1.12%). Accordingly, the transfection efficacy of pDNA in spleen, as reflected by mCherry expression, was much higher in the group of RBC‐hitchhiking nanovaccine than in the pure nanovaccine group at the same injection dose (Fig 2I–K and Appendix Fig S13). Collectively, these results suggest that DNA nanovaccines hitchhiking on RBC at appropriate density (100:1) are able to escape mechanical dislodgement in lungs and then promote DNA‐based vaccine accumulation and neoantigen expression in spleen, which might be favor for eliciting neoantigen‐specific immune response.

### Red blood cell‐hitchhiking DNA nanovaccines to trigger neoantigen‐specific T‐cell response

**Figure 3:**
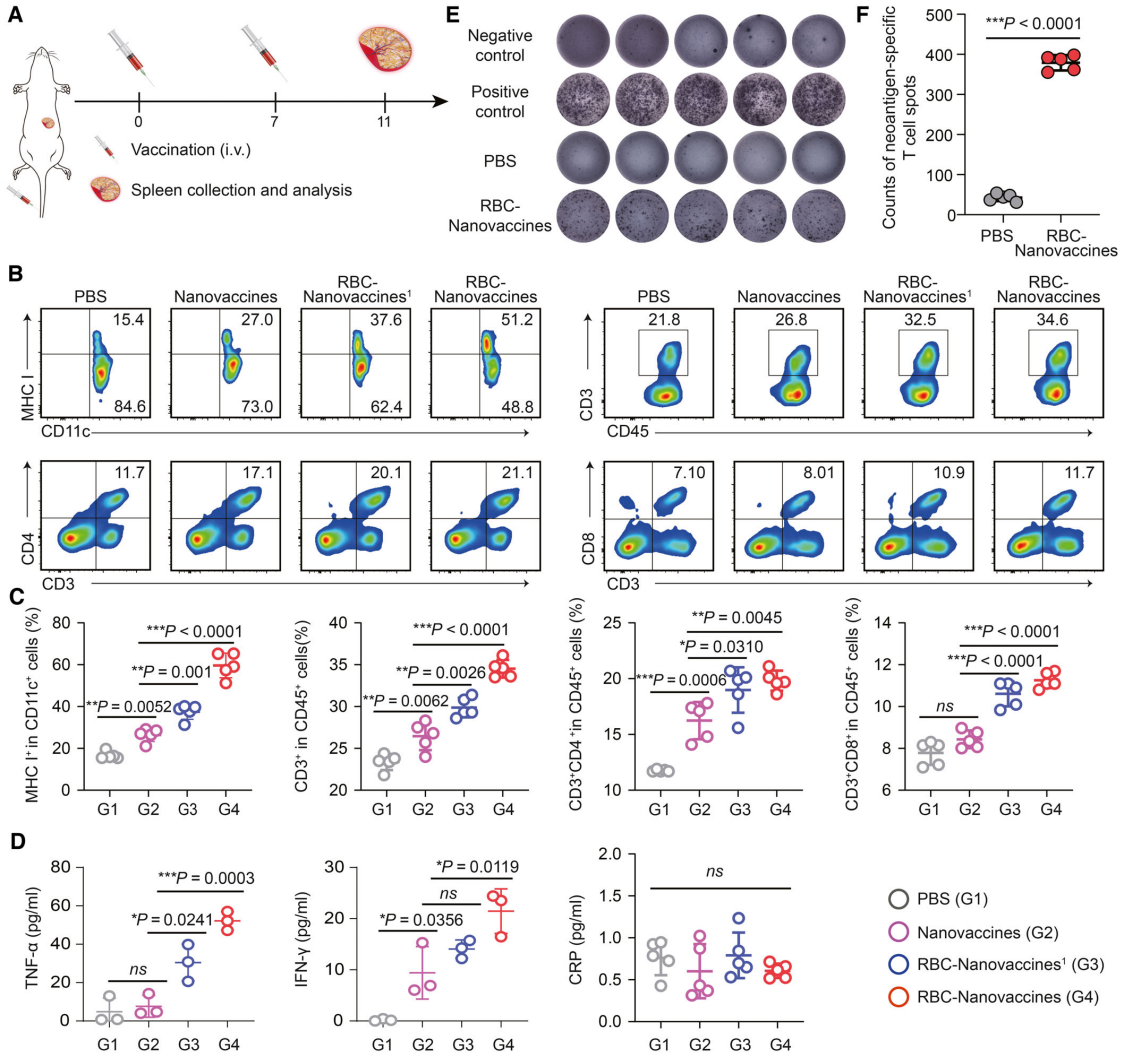
RBC‐hitchhiking improves neoantigen‐specific T‐cell response of DNA nanovaccines A
Scheme of the immunization procedure of RBC‐Nanovaccines.B, C
The expression of MHC I in CD11c^+^ DCs and the percentage of CD45^+^CD3^+^, CD3^+^CD4^+^, and CD3^+^CD8^+^ T cells in the spleen were analyzed by flow cytometry at the 11^th^ day after treatment with PBS, Nanovaccines, and RBC‐Nanovaccines once or twice. *n* = 5 animals per group in (C); data were pooled from two independent experiments.D
ELISA analysis of the concentration of TNF‐α, IFN‐γ, and CRP in the peripheral blood of mice after different treatments (*n* = 3–5 animals per group).E
ELISPOT analysis of IFN‐γ spot‐forming.F
The corresponding quantification numbers of spots in (E). *n* = 5 animals per group; data were pooled from two independent experiments. Data information: Data in (C), (D), and (F) are presented as mean ± SD. Statistical significance was calculated by one‐way ANOVA with Tukey's multiple comparison test in (C, D) and two‐tailed Student's *t*‐test in (F). *P < 0.05, **P < 0.01 and ***P < 0.001. ns means no significant difference. Source data are available online for this figure.

We next examined the immune response triggered by RBC‐hitchhiking DNA vaccines *in vivo*. C57BL/6 mice were immunized twice through i.v. injection with differently engineered vaccines once a week for 2 weeks. Especially for the RBC‐Nanovaccines, we further compared the immune response to that with only one vaccination (designated as RBC‐Nanovaccines^1^). Theoretically, systematically injected vaccines can induce a rapid but not sustained immune response due to their ready diffuse and being cleared up from the host, so two more vaccinations are frequently adopted to elicit durable immune response (“priming + boosting” effect) for cancer treatment (Sahin *et al*, 2020; Zhang *et al*, 2020). Then, 4 days after the second vaccination, the spleen and peripheral blood of the mice were collected for comprehensive immune profiling (Fig 3A). The expression of MHC I on the surface of DC cells in the spleen was analyzed by flow cytometry, and the proportion of MHC I^+^CD11c^+^ DCs in the spleen immunized twice with RBC‐Nanovaccines was 59.64 ± 5.91%, which was significantly higher than that of the control group (with PBS injection; 16.58 ± 1.79%), Nanovaccine group (26.44 ± 3.17%), and RBC‐Nanovaccines^1^ group (37.50 ± 3.60%; Fig 3B and C, and Appendix Fig S14), indicating that the neoantigen encoding pDNA was more effectively captured and expressed by APCs in spleen with the help of RBCs, and then more efficiently triggered the maturation of APCs, which is favorable for mediating the generation of T‐cell immune responses. To verify this hypothesis, the population of T cells in the spleen was analyzed by flow cytometry at the same time. As expected, the RBC‐Nanovaccines group showed significant enhancement in CD45^+^CD3^+^ (effector), CD3^+^CD4^+^ (helper), and CD3^+^CD8^+^ (cytotoxic) T cells in comparison with other groups (Fig 3B and C, and Appendix Figs S15 and S16). Meanwhile, the inoculation of RBC‐Nanovaccines also induced the secretion of antitumor cytokines of tumor necrosis factor‐α (TNF‐α) and interferon‐γ (IFN‐γ) in peripheral blood (Fig 3D). However, both indicators remained within the safe range (Chen *et al*, 2021; Nakamura *et al*, 2022). Additionally, there were no significant changes in C‐reactive protein (CRP) levels after administration (Fig 3D), and no abnormalities were observed in the test animals. These preliminary results demonstrated a low risk of RBC‐Nanovaccines to trigger a cytokine release storm.

**Figure EV2:**
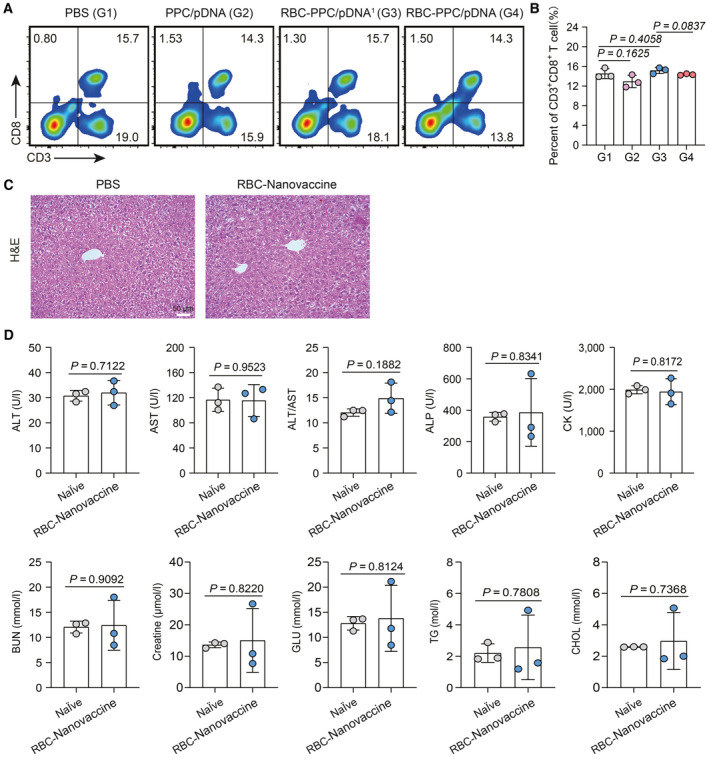
Immune response in the liver tissue after RBC‐Nanovaccine immunization A, B
The percentage of CD3^+^CD8^+^ T cells in the liver after different treatments analyzed by flow cytometry (*n* = 3 animals per group). Data in (B) are presented as mean ± SD. Statistical significance was calculated by two‐tailed Student's *t*‐test. *P* > 0.05 means no significant difference.C
H&E stained micrographs of the liver tissue collected from PBS and RBC‐Nanovaccine treated mice.D
Serum biochemistry data of Naïve group and RBC‐Nanovaccine treated group, including alanine aminotransferase (ALT), aspartate aminotransferase (AST), alkaline phosphatase (ALP), ALT/AST, creatine kinase (CK), blood urea nitrogen (BUN), creatine, glucose acid (GLU), serum total cholesterol (TG), and cholesterol (CHOL) (*n* = 3 animals per group). Data are presented as mean ± SD. Statistical significance was calculated by unpaired two‐tailed Student's *t*‐test (B). *P* > 0.05 means no significant difference.

To evaluate neoantigen‐specific CD8^+^ T‐cell response, T cells were collected from the spleen of mice after the final immunization and then incubated with BMDCs (which were pulsed with the HCC neoantigen peptide in advance) for IFN‐γ detection. This ELISPOT assay demonstrated that inoculation with RBC‐Nanovaccines effectively induced HCC neoantigen‐specific T‐cell immune response, with much more positive spots than the control group (379.00 ± 19.21 vs. 42.8 ± 9.04; Fig 3E and F, and Appendix Fig S17). These results indicate that the RBC‐Nanovaccines can improve the DNA vaccine immunogenicity by co‐transporting polymer–lipid hybrid biocompatible nanoadjuvant and RBCs. However, as a substantial portion of DNA vaccines was also sequestered in liver (Fig 2F), the immune response in the liver tissue was further assessed. The result in Fig EV2A and B revealed that RBC‐Nanovaccine immunization had negligible influence on the frequency of CD3^+^CD8^+^ T cells in liver, which might be due to the weak antigen‐presenting ability of liver resident macrophages such as plasmacytoid dendritic cells, liver sinusoidal endothelial cells, Kupffer cells, and hepatocytes in contrast to professional APCs in spleen after trapping DNA vaccines, thus leading to limited cellular immune effect locally in the liver (Thomson & Knolle, 2010; Atasheva *et al*, 2020). In addition, histological analysis of liver tissue and serum chemistry analysis of ALT, AST, and ALP in the liver, as well as other indicators, also supported the limited immune‐related adverse events caused by our proposed vaccination paradigm in liver for potential clinical translation (Fig EV2C and D).

**Figure EV3:**
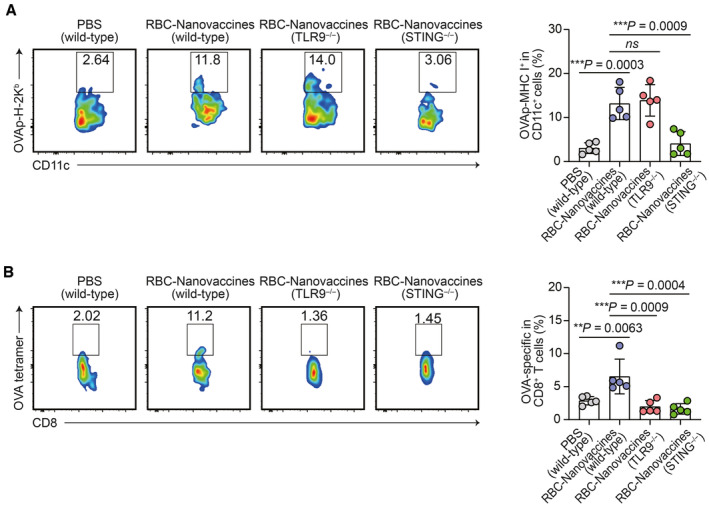
The specific immune response driving by RBC‐Nanovaccines by using OVA as the model antigen Flow cytometry analysis of OVAp‐H‐2K^b^ complex‐positive DCs in C57BL/6 wild‐type, TLR9^−/−^, and STING^−/−^ mice (*n* = 5 animals per group).Flow cytometry analysis of OVAp‐tetramer‐positive CD8^+^ T cells in C57BL/6 wild‐type, TLR9^−/−^, and STING^−/−^ mice (*n* = 5 animals per group). Data information: Data are presented as mean ± SD. Statistical significance was calculated by one‐way ANOVA with Tukey's multiple comparison test. **P < 0.01 and ***P < 0.001.

Furthermore, we performed additional experiments using OVA as a model antigen to examine the impact of RBC‐Nanovaccines on antigen cross‐presentation and the induction of specific T‐cell responses. In line with the vaccination protocol depicted in Fig 3A, we intravenously immunized C57BL/6 wild‐type mice with two doses of RBC‐Nanovaccines carrying pDNA encoding the MHC I‐restricted OVA_257–264_ peptide (SIINFEKL). In addition, we included C57BL/6 mice that are genetically deficient for STING (STING^−/−^) or TLR9 (TLR9^−/−^) as the control groups because these two proteins are crucial in the DNA sensing pathways for initiating immune responses. As shown in Fig EV3, after immunization with RBC‐Nanovaccines, the presentation of OVA_257–264_ by MHC I H‐2K^b^ (H‐2K^b^‐OVA) was clearly detected in splenic CD11c^+^ DCs from wild‐type mice and TLR9^−/−^ mice, whereas there was almost no H‐2K^b^‐OVA positive signal in the splenic CD11c^+^ DCs collected from STING^−/−^ mice. Next, we further determined the OVA‐specific T cells in splenocytes. Remarkably, immunization with RBC‐Nanovaccines resulted in a much higher proportion of antigen‐specific T cells in wild‐type mice, with the percentage of OVA tetramer^+^ in CD3^+^CD8^+^ cells reaching 6.55 ± 2.64%. In comparison, the percentage observed in STING^−/−^ mice was 1.64 ± 0.79% and in TLR9^−/−^ mice was 1.97 ± 0.94%. In TLR9^−/−^ mice, antigenic epitopes were found to be presented on DC cells, but no antigen‐specific T cells were detected. This absence of antigen‐specific T cells can be attributed to the essential role of TLR9 signaling pathways in T cells, which are required for their effective clonal expansion (Rahman *et al*, 2009). Collectively, these results demonstrate that RBC‐Nanovaccines can induce an antigen‐specific immune response, which is closely associated with the DNA‐activated STING and TLR9 pathways.

### Personalized immunity of RBC‐hitchhiking DNA nanovaccines against HCC

**Figure 4:**
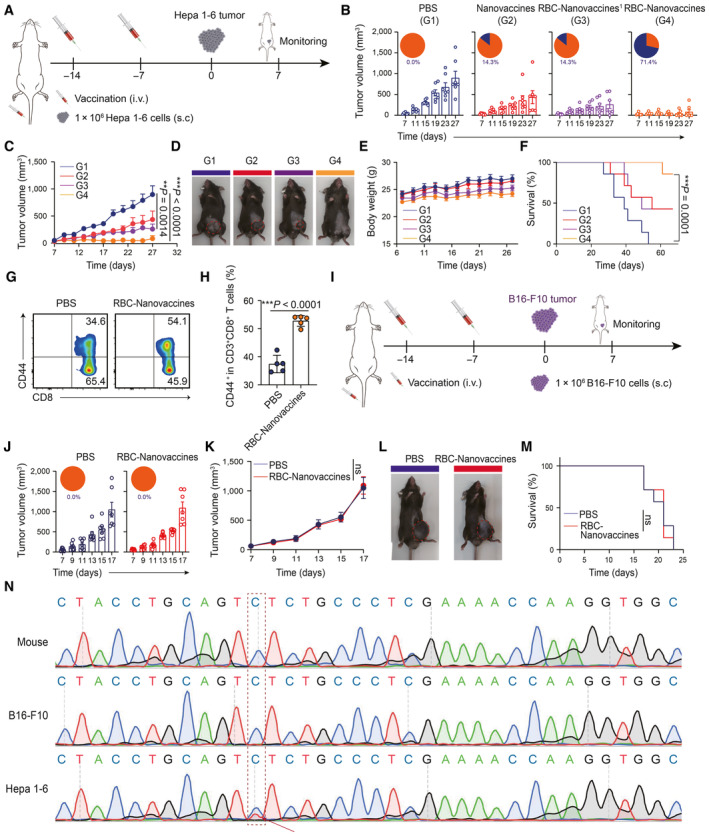
RBC‐hitchhiking DNA nanovaccines evoked personalized prophylactic protection with immunological memory A
Schematic illustration of the schedule for the prophylactic protection against Hepa 1‐6 tumor.B, C
Individual Hepa 1‐6 tumor growth with fraction of tumor‐free (TF) (B) and average tumor growth curves (C), after inoculation with PBS, Nanovaccines, and RBC‐Nanovaccines once or twice (*n* = 7 animals per group).D
Representative images of Hepa 1‐6 tumor‐bearing mice in each group at the 27^th^ day after receiving different treatments as indicated. The tumor area was labeled with red dashed circle.E, F
The body weight change (E) and survival curves (F) of Hepa 1‐6 tumor‐bearing mice after receiving different treatments as indicated (*n* = 7 animals per group).G, H
The percentage of CD8^+^CD44^high^ memory T cells in the spleen was analyzed by flow cytometry on the 7^th^ day after the second vaccination of PBS and RBC‐Nanovaccines. *n* = 5 animals per group in (H); data were pooled from two independent experiments.I
Schematic illustration of the schedule for the prophylactic protection against B16‐F10 tumor.J, K
Individual B16‐F10 tumor growth with fraction of tumor‐free (TF) (J) and average tumor growth curves (K), after inoculation with PBS and RBC‐Nanovaccines twice (*n* = 7 animals per group).L
Representative images of Hepa 1‐6 tumor‐bearing mice in each group at the 17^th^ day after receiving different treatments as indicated.M
The survival curves of various treated mice (*n* = 7 animals per group).N
DNA sequencing results of mutated genes for neoantigen encoding in mouse normal tissue, B16‐F10, and Hepa 1‐6 tumor. Data information: Data in (B, C, E, J, and K) are presented as mean ± SEM, while data in (H) are presented as mean ± SD. Statistical significance was calculated by two‐tailed Student's *t*‐test in (H), one‐way ANOVA with Tukey's multiple comparison test in (C, K), and log‐rank (Mantel‐Cox) test in (F, M). *P < 0.05, **P < 0.01 and ***P < 0.001. ns means no significant difference. Source data are available online for this figure.

To further evaluate the feasibility of RBC‐hitchhiking DNA nanovaccines (RBC‐Nanovaccines) to exert immune‐protective effect against tumorigenesis, the immunized mice aforementioned above were subcutaneously (s.c.) challenged with Hepa 1‐6 cells at the 7 days after the second vaccination (Fig 4A). The tumors of mice inoculated with PBS suffered from rapid growth, whereas mice treated with Nanovaccines and a single inoculation of RBC‐Nanovaccine moderately delayed the tumor growth but still failed to prevent tumor progression. Notably, two inoculations with RBC‐Nanovaccines achieved the highest efficiency in preventing tumor growth with 71.4% (5/7) remaining tumor‐free, compared with the PBS (0/7, 0.0%), Nanovaccines (1/7, 14.3%), and RBC‐Nanovaccines^1^ groups (1/7, 14.3%) at the day 27 (Fig 4B–D and Appendix Fig S18). Meanwhile, none of the mice showed an obvious body weight loss during vaccination (Fig 4E), indicating the limited side effects of the above treatments. Furthermore, RBC‐Nanovaccines significantly prolonged the overall survival of the mice, and only one mouse (1/7) had to be killed due to the tumor volume exceeding the limits of ethical regulations throughout the course of the study, whereas mice in other groups all succumbed to tumors with limited survival benefits (Fig 4F). As immunological memory has been reported to be one of the most important factors induced by the prophylactic vaccine contributing to tumor rejection (Nguyen *et al*, 2020), we further investigated the memory T‐cell subtype in the spleen by flow cytometry on the 7^th^ day after the second vaccination. Compared to naïve mice (PBS group), RBC‐Nanovaccine vaccinated mice exhibited a 1.5‐fold higher percentage of CD8^+^ memory T cells (CD8^+^CD44^high^), which can be reactivated upon the secondary tumor challenge (Fig 4G and H). Taken together, the RBC‐Nanovaccines could efficiently induce CD8 memory T cells to suppress tumor growth in a prophylactic setting.

In comparison, the immunized mice were subcutaneously (s.c.) challenged with B16‐F10 cells to assess the personalized antitumor responses elicited by RBC‐Nanovaccines specifically against Hepa 1‐6 tumors (Fig 4I). RBC‐Nanovaccines were found to be powerless in the face of B16‐F10 melanoma cell challenge, as the immunized mice presented the same rapid tumor growth trend as those in PBS control group without any survival benefits (Fig 4J–M and Appendix Fig S19). To further explore the underlying mechanism, the Hepa 1‐6 tumors, B16‐F10 tumors, and mouse tail tissues were harvested for complementary DNA (cDNA) sequencing. As shown in Fig 4N, B16‐F10 tumors and normal tissues did not contain mutated genes since neoantigen encoding unique to Hepa 1‐6 cells, supporting the selective preventive role of our DNA vaccine against Hepa 1‐6 tumor in a personalized manner.

**Figure EV4:**
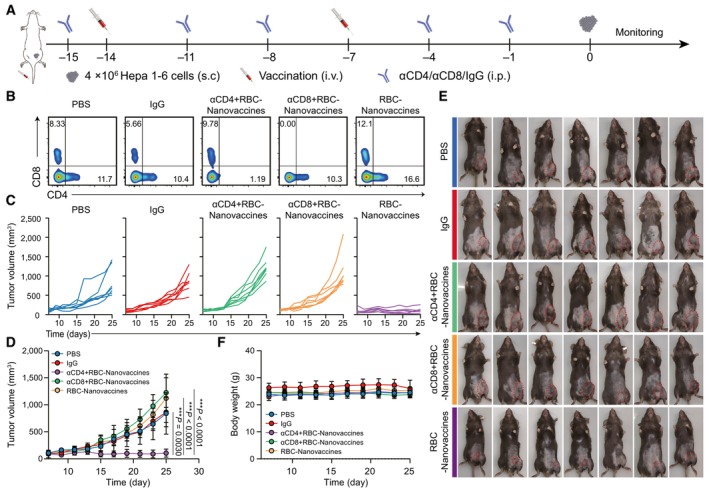
RBC‐Nanovaccines evoke prophylactic protection through CD4^+^ T and CD8^+^ T dependent cell responses A
Schematic illustration of the schedule for the prophylactic protection against Hepa 1‐6 tumor.B
The percentages of CD4^+^ and CD8^+^ T cells in the peripheral blood after receiving different treatments, which were analyzed by flow cytometry on the 0^th^ day.C, D
Individual growth curves (C) and average tumor growth curves (D) after receiving different treatments (*n* = 7 animals per group).E
Representative images of Hepa 1‐6 tumor‐bearing mice in each group at the 25^th^ day. The tumor area was labeled with red dashed circle.F
The body weight change of Hepa 1‐6 tumor‐bearing mice during the course of various treatments (*n* = 5 animals per group). Data information: Data are presented as mean ± SD. Statistical significance was calculated by one‐way ANOVA with Tukey's multiple comparison test. ***P < 0.001.

To further assess the role of T‐cell populations in preventing tumorigenesis, we conducted additional experiments to investigate the effects of RBC‐Nanovaccines on tumor growth in mice depleted of CD8^+^ or CD4^+^ T cells. This was achieved by intraperitoneal injection of the corresponding neutralizing antibodies. The experimental timeline is illustrated in Fig EV4A. CD8^+^ and CD4^+^ T‐cell depletion was confirmed by flow cytometric analysis of peripheral blood mononuclear cells (PBMCs; Fig EV4B). Continuous depletion studies (Fig EV4C–E) revealed that the antitumor effects of RBC‐Nanovaccines were largely abolished upon depletion of CD8^+^ T cells. Surprisingly, the protective effect was also lost after depletion of CD4^+^ T cells, although the selection of neoantigens was based on their affinity to MHC I H‐2K^b^. These findings collectively demonstrate that the antitumor response induced by RBC‐Nanovaccines depends on CD8^+^ T cells through CD4^+^ T‐cell‐dependent mechanisms. It has been well reported that CD4^+^ T cells play a synergistic role with CD8^+^ T cells by recruiting them to the tumor site for enhancing their proliferation and effector functions (Marzo *et al*, 1999; Bos & Sherman, 2010).

### RBC‐hitchhiked DNA nanovaccines combined with immune checkpoint inhibitor of anti‐PD‐1 to trigger potent antitumor therapeutic effect

**Figure 5:**
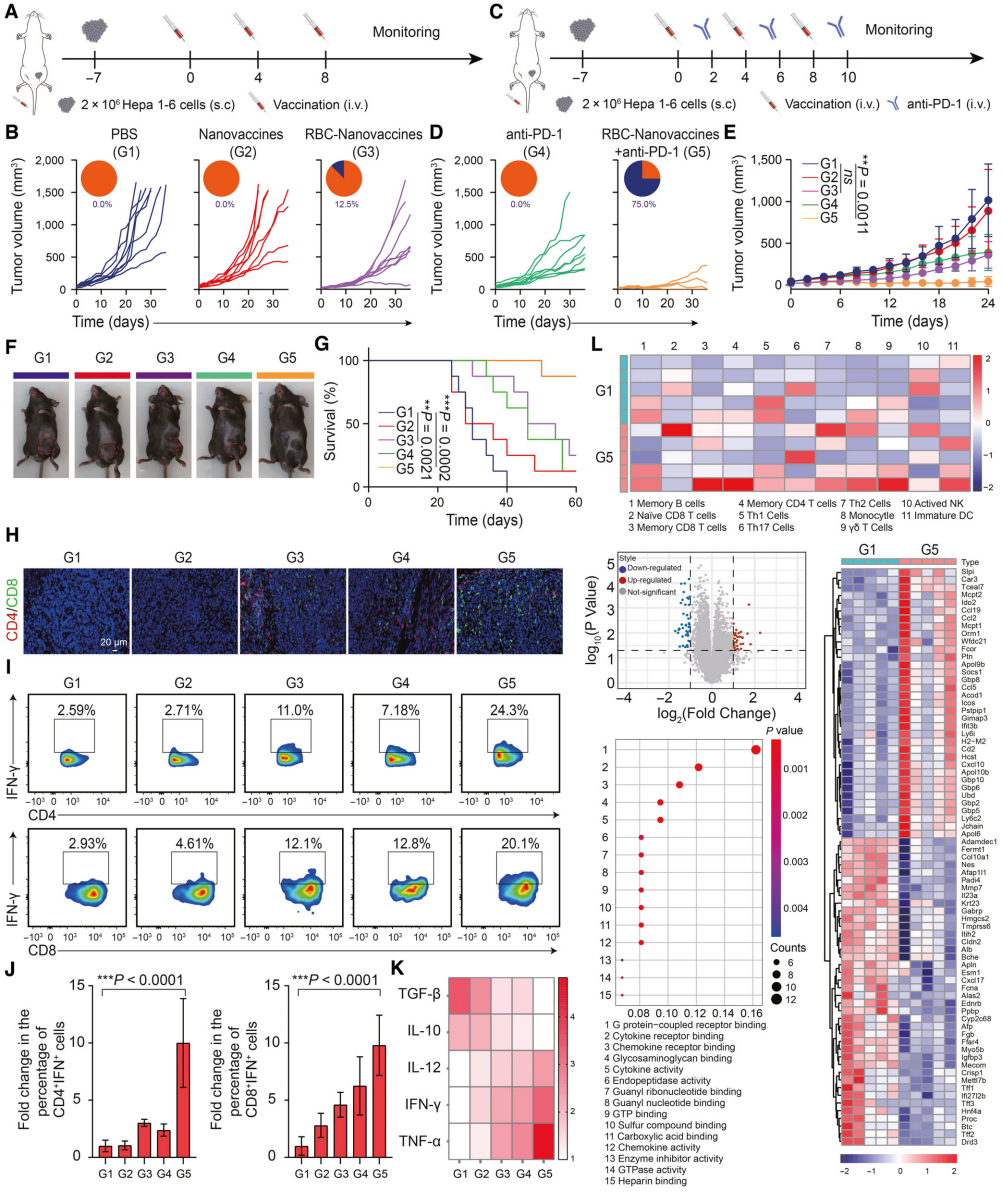
The RBC‐hitchhiking DNA nanovaccines combines with anti‐PD‐1 antibody to inhibit established Hepa 1‐6 HCC tumor A
Schematic illustration of the schedule for the monotherapy of RBC‐Nanovaccines on Hepa 1‐6 tumor.B
Individual Hepa 1‐6 tumor growth with fraction of completely tumor regression (CR) after inoculation with PBS, Nanovaccines, and RBC‐Nanovaccines for three times (*n* = 8 animals per group).C
Schematic illustration of the schedule for the combination therapy of RBC‐Nanovaccines and anti‐PD‐1 antibody on Hepa 1‐6 tumor.D, E
Individual Hepa 1‐6 tumor growth with fraction of completely tumor regression (CR) (D) and average tumor growth curves (E), after receiving different treatments as indicated (*n* = 8 animals per group).F
Representative images of Hepa 1‐6 tumor‐bearing mice in each group at the 24^th^ day after receiving different treatments as indicated.G
The survival curves of Hepa 1‐6 tumor‐bearing mice after receiving different treatments as indicated (*n* = 8 animals per group).H
Immunofluorescence staining of tumor infiltrated CD4^+^ T cells (red) and CD8^+^ T cells (green) on the 24^th^ day after receiving different treatments as indicated.I, J
The percentage of IFN‐γ^+^CD4^+^ and IFN‐γ^+^CD8^+^ T cells in the tumor after receiving different treatments was analyzed by flow cytometry on the 24^th^ day. *n* = 5 animals per group in (J); data were pooled from two independent experiments.K
Heat map analysis showing the concentration of TGF‐β, IL‐10, IL‐12, IFN‐γ, and TNF‐α in the tumor from mice after different treatments.L
Transcriptomic analysis of Hepa 1‐6 tumor tissues after the combinational treatment of RBC‐Nanovaccines and anti‐PD‐1 antibody, including heatmap of the associated immune cells and volcano plot, GO pathway, and heatmap of DEGs. Data information: Data in (E) are presented as mean ± SEM, while data in (J) are presented as mean ± SD. Statistical significance was calculated by one‐way ANOVA with Tukey's multiple comparison test in (E, J) and Gehan–Breslow–Wilcoxon test in (G). **P < 0.01 and ***P < 0.001. ns means no significant difference. Source data are available online for this figure.

**Figure EV5:**
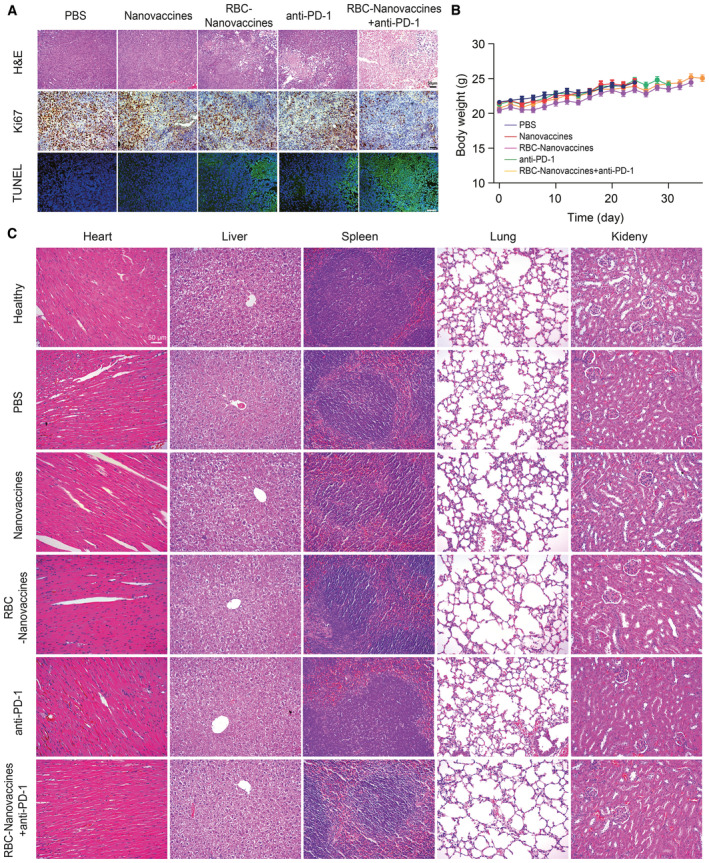
Therapeutic effects of the RBC‐hitchhiking DNA nanovaccine combining with anti‐PD‐1 antibody in the established Hepa 1‐6 HCC tumor model Optical microscopy images of H&E, Ki67 antigen immunohistochemistry staining, and TUNEL staining of tumor slices after receiving different treatments. Scale bar: 50 μm.Body weight change of Hepa 1‐6 tumor‐bearing mice after vaccination as indicated. Data are expressed as mean ± SEM (*n* = 8 animals per group).Histological analysis of H&E staining of mice major organs (heart, liver, spleen, lung, and kidney) collected from Hepa 1‐6 tumor bearing mice receiving indicated treatments.

Having confirmed the personalized immunization for HCC prevention, we next evaluated the antitumor efficacy of RBC‐Nanovaccines in the established HCC model with an immune “cold” microenvironment which inhibits T‐cell infiltration (Appendix Fig S20). The C57BL/6 mice were s.c. inoculated with Hepa 1‐6 cells on day 7 in advance and then immunized with PBS, Nanovaccines, or RBC‐Nanovaccines three times through i.v. injection on days 0, 4, and 8 (Fig 5A). As shown in Fig 5B, compared with PBS and Nanovaccine groups which suffered from rapid tumor growth, the mice treated with RBC‐Nanovaccines significantly delayed tumor growth. Regrettably, RBC‐Nanovaccines as a monotherapy failed to induce tumor regression, with only one out of eight mice responding very well (Fig 5B), probably ascribing to other negative immune‐regulating pathways to assist the escape of HCC cells from immune surveillance. Interestingly, the most common negative immune checkpoint PD‐L1 was found to be obviously upregulated in tumor tissues of RBC‐Nanovaccines‐treated mice according to immunofluorescence (IF) and immunohistochemistry (IHC) analysis (Appendix Fig S21). The PD‐1 and PD‐L1 axis between T cells and tumor cells could lead to T‐cell anergy, which might be the reason for relatively poor response to our RBC‐Nanovaccines. However, such disappointing phenomena can be rescued by the checkpoint inhibitors of anti‐PD‐1 and anti‐PD‐L1 that have achieved great success in many solid tumors including HCC but are still restricted by low immune response in clinic. Thus, to unleash the full potential of the neoantigen‐specific T‐cell responses, we further combined RBC‐Nanovaccines with an immune checkpoint inhibitor (anti‐PD‐1; Fig 5C). Unsurprisingly, the anti‐PD‐1 alone did not induce tumor regression (0/8, 0.0%) despite an early delay in tumor growth. However, when combined with RBC‐Nanovaccines, the tumor regression rate increased to 75.0% (6/8) with complete cure at day 36 (Fig 5D–F; Appendix Fig S22), thus showing the highest therapeutic effect among all groups. Accordingly, the combination therapy substantially prolonged the overall survival of mice (Fig 5G). Subsequently, the antitumor activity of RBC‐Nanovaccines combined with anti‐PD‐1 was analyzed by hematoxylin and eosin (H&E), Ki67, and TUNEL staining of tumor slices, demonstrating that the combination therapy had an excellent antitumor effect (Fig EV5A). In addition, the mice receiving different treatments showed no significant body weight loss and no obvious damage to major organs (Fig EV5B and C). These results verify that the combination of RBC‐Nanovaccines and anti‐PD‐1 antibody further improves the antitumor efficacy.

To further elucidate the antitumor immune response of RBC‐Nanovaccines combined with anti‐PD‐1, the infiltration of effector T cells in the tumor was analyzed by immunofluorescence staining, and the results displayed that the most enrichment of fluorescent signals of CD4^+^ T cells and CD8^+^ T cells was observed in the combination treatment group compared to other treated groups (Fig 5H and Appendix Fig S20). Meanwhile, the RBC‐Nanovaccines plus anti‐PD‐1 remarkably enhance the repertoires of IFN‐γ^+^CD4^+^ T cells and IFN‐γ^+^CD8^+^ T cells (Fig 5I and J, and Appendix Fig S23), thus potentiating the adaptive immune response for tumor control (Zhu *et al*, 2017; Zhao *et al*, 2021). Meanwhile, we analyzed the cytokines in the tumor by ELISA, and the analysis showed that the combination therapy significantly increased the immunostimulatory cytokines (TNF‐α, IFN‐γ, and IL‐12) and decreased the immunosuppressive cytokines (IL‐10 and TGF‐β) compared with the monotherapy (Fig 5K and Appendix Fig S24). Then, we further elucidate the underlying mechanisms by evaluating the transcriptomic signature of tumor samples after combination therapy. The heatmap analysis from RNA‐seq data demonstrated that the combination therapy significantly enhanced immune cell infiltration (memory B cells, memory CD8 T cells, NK cells, and macrophages) in the tumor (Fig 5L). Subsequently, volcano plot, GO pathway, and heat map analysis of differentially expressed genes (DEGs) showed that the upregulated genes after combination treatment with RBC‐Nanovaccines and anti‐PD‐1 were mainly involved in T‐cell activation, cytokine and chemokine secretion, and inflammatory responses (Fig 5L). Thus, these results indicate that our RBC‐hitchhiking DNA nanovaccines are able to fight against aggressively growing HCC tumor when combined with immune checkpoint blockade to unleash the immune negative regulating signals.

**Figure 6:**
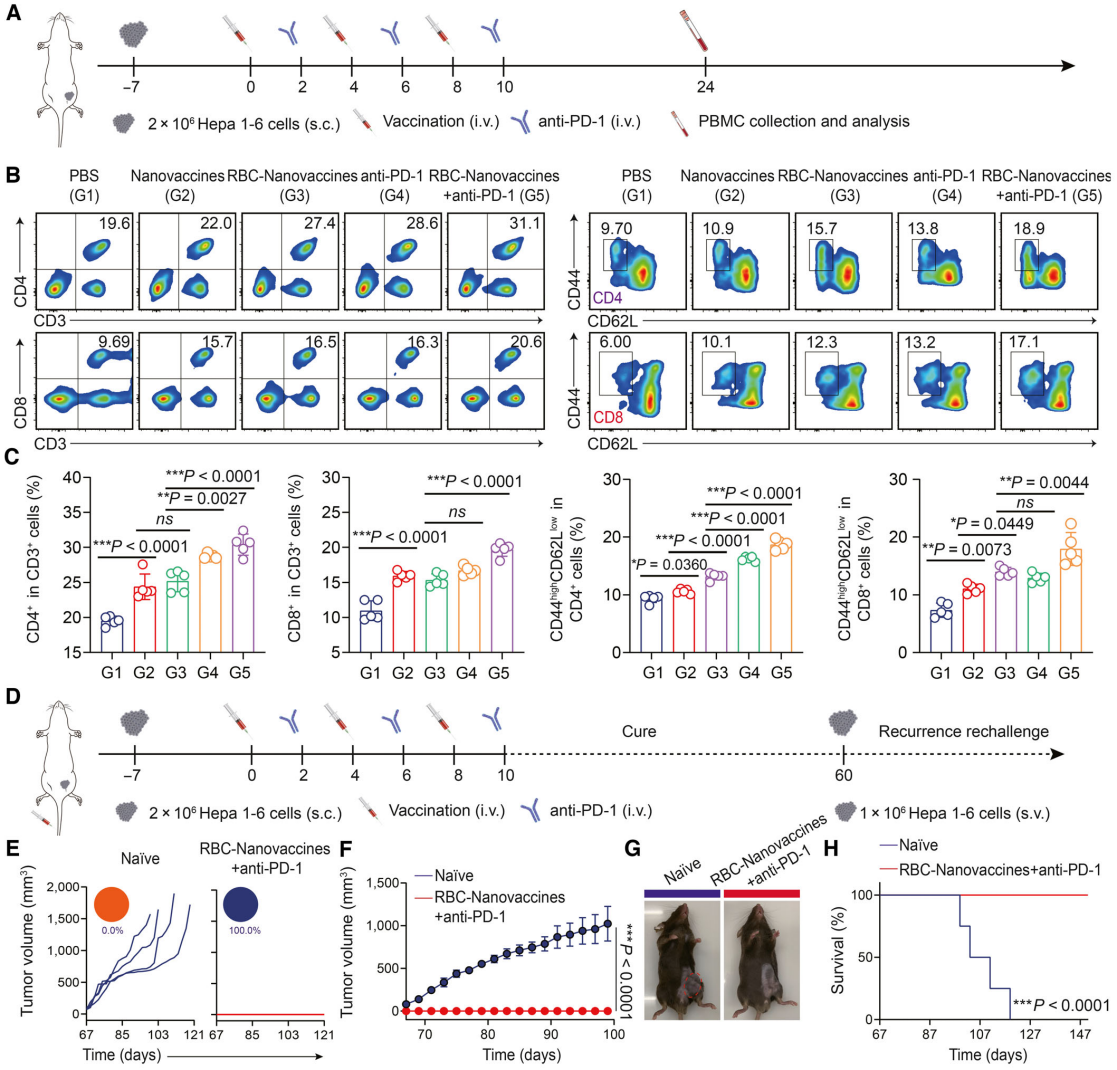
RBC‐hitchhiking DNA nanovaccines combining with anti‐PD‐1 antibody to evoke durable immunological memory to suppress Hepa 1‐6 tumor recurrence A
Schematic illustration of the schedule for peripheral blood collection of mice after different treatments.B, C
Percentage of CD3^+^CD4^+^, CD3^+^CD8^+^, and memory T cells (CD4^+^CD44^high^CD62L^low^ and CD8^+^CD44^high^CD62L^low^) in the peripheral blood at the 24^th^ day after receiving different treatments. *n* = 5 animals per group in (C); data were pooled from two independent experiments.D
Schematic illustration of the schedule for the tumor s.c. re‐challenge.E, F
Individual Hepa 1‐6 tumor growth with fraction of completely tumor regression (CR) (E) and average tumor growth curves (F), after s.c. re‐challenge of Hepa 1‐6 cells. *n* = 4 animals per group in (F).G
Representative images of Hepa 1‐6 tumor‐bearing mice in each group at the 100^th^ day after subcutaneous rechallenge.H
The survival curves of Hepa 1‐6 tumor‐bearing mice after subcutaneous rechallenge (*n* = 4 animals per group). Data information: Data in (C) are presented as mean ± SD, while data in (F) are presented as mean ± SEM. Statistical significance was calculated by one‐way ANOVA with Tukey's multiple comparison test in (C, F) and log‐rank (Mantel‐Cox) test in (H). *P < 0.05, **P < 0.01, and ***P < 0.001. ns means no significant difference. Source data are available online for this figure.

Next, to further determine whether the systemic immune responses are evoked after receiving the combinational therapy, we analyzed the effector T cells in the peripheral blood of mice receiving different treatments by flow cytometry (Fig 6A), and the analysis demonstrated that the combinational therapy induced more frequency of CD3^+^CD4^+^ and CD3^+^CD8^+^ T cells in blood circulation system (Fig 6B and C, and Appendix Fig S25). Furthermore, the fraction of effector memory T cells (CD4^+^CD44^high^CD62L^low^ and CD8^+^CD44^high^CD62L^low^) was significantly increased in the combination treatment group compared with the untreated group (*P* < 0.001) in the peripheral blood (Fig 6B and C, and Appendix Fig S25), demonstrating that combination treatment with RBC‐Nanovaccines and anti‐PD‐1 antibody could elicit a long‐lasting memory immune response to prevent tumor recurrence. To prove this hypothesis, the cured mice were re‐inoculated in the contralateral flank with Hepa 1‐6 cells on day 60 again (Fig 6D). Notably, 100% of the cured mice prevented the re‐challenge with Hepa 1‐6 tumor cells, whereas the mice in the naïve group were unable to reject tumor growth (Fig 6E–H and Appendix Fig S26), indicating an effective immunological memory against tumor recurrence.

**Figure 7:**
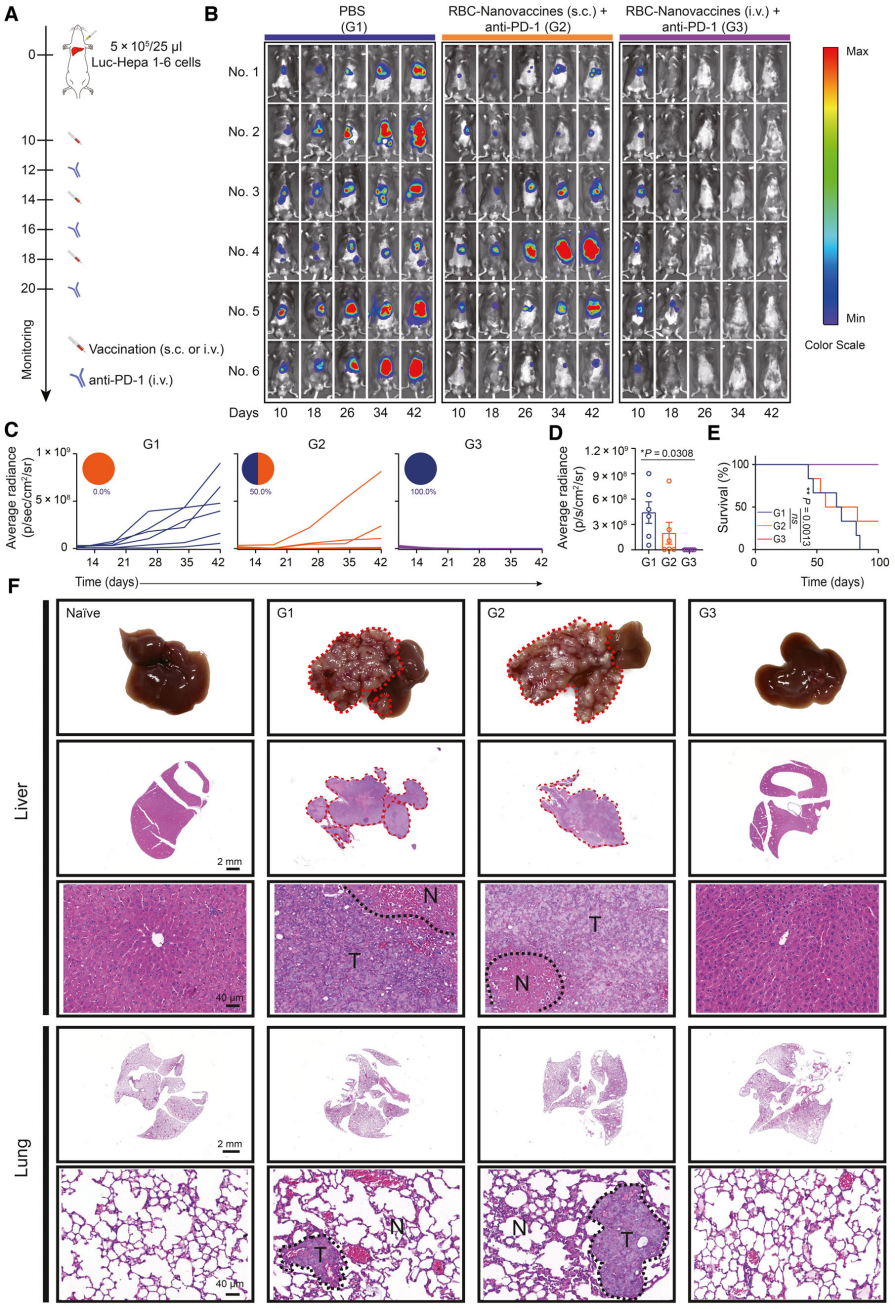
The RBC‐hitchhiking DNA nanovaccines combining with anti‐PD‐1 antibody to eliminate orthotopic Hepa 1‐6 tumors and inhibit tumor metastasis Schematic illustration of the schedule for subcutaneous (s.c.) or intravenous (i.v.) injection of the RBC‐Nanovaccines in the orthotopic Hepa 1‐6 tumor models.Bioluminescence imaging of mice in each group on days 10, 18, 26, 34, and 42 as indicated.Individual orthotopic Hepa 1‐6 tumor growth (measured by average radiance) after subcutaneous or intravenous inoculation with RBC‐Nanovaccines three times (*n* = 6 animals per group).The average radiation statistics for each group at 42 days.The survival curves of orthotopic Hepa 1‐6 tumor‐bearing mice after receiving different treatments as indicated (*n* = 6 animals per group).Representative images of the liver and orthotopic tumors of mice in each group on day 43 after receiving different treatments are indicated, and the H&E staining of liver tissues after receiving different treatments is indicated. Data information: Data in (D) are presented as mean ± SD. Statistical significance was calculated by two‐tailed Student's *t*‐test in (D) and log‐rank (Mantel‐Cox) test in (E). *P < 0.05 and **P < 0.01. ns means no significant difference. Source data are available online for this figure.

Encouraged by the above results, we further explored the immunotherapy effect of the RBC‐Nanovaccines through different immunization routes. Meanwhile, to more closely mimic the microenvironment where tumor cells grow in liver, we constructed an orthotopic HCC mouse model to evaluate the therapeutic efficacy. Luc‐Hepa 1‐6 cells were surgically inoculated into the liver lobes of C57BL/6 mice on day 0 and RBC‐Nanovaccines were injected three times on days 10, 14, and 18 (Fig 7A). Tumor burden was measured by bioluminescence imaging every 8 days from days 10 to 42. As shown in Fig 7B, compared with the PBS group that suffered from rapid tumor growth, the mice treated by the RBC‐Nanovaccines plus anti‐PD‐1 antibody could effectively delay tumor growth regardless of the vaccination route such as systemic administration (i.v.) or topical injection (s.c.), but subcutaneous inoculation of RBC‐Nanovaccines ultimately failed to induce tumor regression, with three out of six mice showing intensified signals on day 42 (3/6, 50.0%). Strikingly, intravenous injection (systemic administration) of RBC‐Nanovaccines could undoubtedly improve the response rate of immunotherapy (6/6, 100.0%), resulting in complete tumor regression and significantly prolonged survival time over the course of the study for more than 100 days (Fig 7C–E). Additionally, the therapeutic effect was further verified by photographing the liver and H&E staining analysis of organ sections (Fig 7F). Not only primary tumor but also the micro‐metastases that appeared in the lung tissue were also dramatically suppressed by the combination of RBC‐Nanovaccines and anti‐PD‐1 through systemic administration (Fig 7F). The superior therapeutic efficacy of RBC‐Nanovaccines through i.v. immunization route is ascribed to their spleen‐targeted ability to induce a rapid and strong antitumor immune response (Han *et al*, 2019; Zhai *et al*, 2021), whereas the immunity elicitation via s.c. route needs a much longer time and suffers from a low strength due to the limited APC activation and slow migration from the injection site to lymph node for T‐cell activation (Itano & Jenkins, 2003). Collectively, these results demonstrate that our proposed DNA neoantigen vaccine in virtue of the RBC‐based cascade delivery strategy especially through a systemic vaccination route has shown great promise to reinforce checkpoint blockade therapy to eradicate both primary tumor and spontaneous metastasis of advanced‐stage HCC.

## Discussion

NCVs have shown great potential for personalized antitumor therapy in several solid tumors with high TMB in early human clinical trials, but their application in tumors characterized as low or moderate TMB, such as HCC, is still a challenge. Up to now, only a total of nine clinical trials for HCC treatment by using DC‐ or peptide‐based NCVs have been registered for clinical investigation, while no impressive results have yet been reported (http://www.clinicaltrials.gov). Compared with DC and peptide formulations, DNA vaccines can evoke a long‐term immune response as a means of persistently encoding antigenic proteins once successfully being transfected in APCs. Recently, a DNA‐based neoantigen vaccine (GNOS‐PV02) administrated via intradermal injection in combination with electroporation was evaluated in patients with advanced HCC (NCT04251117). Unfortunately, the immunogenicity and therapeutic outcome of this DNA vaccine remain in need of improvement, as evidenced by an objective response rate of only 25%, even when used in combination with PD‐1 inhibitors and IL‐12, as observed in the latest single‐arm phase I/II clinical trial (Yarchoan *et al*, 2021; Perales *et al*, 2022). An additional issue regarding DNA vaccines in clinic trials is that all of them employ electroporation technic to transport DNA, which not only causes discomfort and pain to the patients but also needs a specialized equipment and technician involvement, thus restricting the application of this strategy (Nguyen *et al*, 2020). Alternatively, various nanomaterial‐based delivery systems have been explored to deliver DNA vectors in animal models with varying degrees of success, but how to direct them to a specific site (APCs in lymphoid tissues in this case) for unleashing their full potential as personalized therapeutics remains one of the major challenges. In this study, we proposed a cascade delivery strategy to transport DNA‐based neoantigen vaccine to spleen with abundant APCs and T cells for driving personalized antitumor immunity for HCC.

To regulate the *in vivo* biodistribution of our nanovaccines and achieve efficient enrichment in spleen rather than in liver, where NPs are often trapped after systemic administration, RBC‐hitchhiking strategy was employed in this work by leveraging the natural properties of RBC to hand off pathogens on its surface to the immune cells in the spleen (Minasyan, 2018). In this regard, Mitragotri group has done a series of pioneering works for our reference (Brenner *et al*, 2018; Zhao *et al*, 2019; Ukidve *et al*, 2020; Ferguson *et al*, 2022), whereas the potential of such a strategy in DNA‐based neoantigen delivery to yield robust specific immunity for personalized cancer therapy has not been reported. According to their report (Ukidve *et al*, 2020), the hitchhiking of nanoparticles involves two physical steps. First, nanoparticles adsorb to the surface of erythrocytes, initiating contact. Second, the erythrocyte membrane spreads around the nanoparticles, enhancing the strength of adhesion. Using PLGA nanoparticles as an example, they demonstrated that the binding between nanoparticles and red blood cells could be facilitated through noncovalent forces such as electrostatic interactions, hydrophobic interactions, and hydrogen bonding (Zhao *et al*, 2021). Our nanovaccine primarily utilizes PLGA as a carrier, thereby allowing for adsorption to the surface of red blood cells through these three types of interactions. Notably, the nanovaccine formulation also contains a PEI‐C_14_ cationic component that imparts a positive charge to the PLGA surface, further enhancing the binding to red blood cells via electrostatic interactions. Noticeably, this RBC‐hitchhiking nanovaccine has to face the challenge of the first‐pass entrapment in lung before entering in downstream organs like spleen, since the high shear stresses in lung capillaries will dislodge particles on RBCs (Zhao *et al*, 2019). Though adjusting the initial feed ratio of nanovaccines to RBC, the distribution of nanovaccines in our study was successfully shifted from lung to spleen, by enhancing shear resistance to avoid serious detachment in lung and enable a large fraction available to spleen.

By this design, our RBC‐hitchhiking nanovaccines elicited significantly stronger cellular immune responses than pure nanovaccines. Although very effective in preventing tumor occurrence, RBC‐hitchhiking nanovaccines as a monotherapy failed to induce tumor regression in the established HCC tumor model with an immune “cold” microenvironment (Appendix Fig S20). One reason probably accounting for treatment failure is the upregulation of multiple immune checkpoints such as PD‐1 and PD‐L1, which can be induced by a variety of cytokine secretion under treatment including IFN‐γ and TNF‐α in tumor microenvironment (Garcia‐Diaz *et al*, 2017; Jiang *et al*, 2019). Unsurprisingly, our RBC‐Nanovaccines promoted the secretion of polyfunctional IFN‐γ and TNF‐α from neoantigen‐specific T cells during treatment (Fig 5J and K), which has been reported to be not always beneficial for antitumor treatment, and in some cases, also contribute to a feedback loop of PD‐1/PD‐L1 expression (Appendix Fig S21) to mediate immune escape of cancer cells. However, the upregulation of PD‐1/PD‐L1 can be leveraged as an ideal target for immune checkpoint inhibitors (ICIs), in view of its great value as a predictive biomarker for sensitivity to ICIs (Davis & Patel, 2019). Thus, we rationally combine RBC‐Nanovaccines with anti‐PD‐1 antibody to provide optimal activation of T cells for the elimination of aggressively growing HCC tumors, which is also adopted in current studies and ongoing trials in clinic (Hu *et al*, 2018).

Despite the great potential of our RBC‐Nanovaccines to the overall antitumor efficacy, several aspects of the RBC‐hitchhiking DNA nanovaccines proposed in this work need to be explored in the future. First, the plasmid DNA can be further improved to encode multiple neoantigens to address the challenge of clonal heterogeneity of tumor cells and then elicit more robust and broader antitumor‐specific responses with less opportunity for immune escape. Second, there is still a lack of direct evidence to support the generation of antigen‐specific T cells in the mice after receiving RBC‐Nanovaccines because the acquisition of neoantigen peptide‐MHC tetramer indispensable in this assay is a time‐ and labor‐consuming task. Third, the influences of DNA nanovaccines on RBC have not been thoroughly studied, such as membrane alterations, agglutination, and expression of surface markers. A previous study showed that these variations also mediated the interactions of APCs with RBCs (Ukidve *et al*, 2020). That is, if we can develop various types of vehicles to fabricate DNA nanovaccines, perhaps the spleen targeting ability of these RBC‐hitchhiking nanovaccines can be further optimized. In addition, the nanovaccine's surface can be further modified with APC affinity ligand to increase its bioavailability, under the premise of spleen enrichment (Ferguson *et al*, 2022). Moreover, NCVs based on mRNA with an excellent safety profile, flexibility, and potency would also be a promising target worthy of further study by using our polymer‐nanoparticulated and RBC‐hitchhiked cascade delivery strategy. Lastly, the vaccination dose and frequency should also be considered to improve the therapeutic outcome.

Overall, this study provides a highly efficient immunization paradigm by hitchhiking neoantigen DNA nanovaccines on RBC to drive personalized antitumor immunity for HCC treatment, which is also ideally suited for reinforcing the ICIs with a striking therapeutic efficacy. Since this delivery strategy is easily tailored to different nucleic acid‐based vaccines encoding various antigens, it could afford a universal solution for the development of potent neoantigen vaccines to boost personalized antitumor immunity in multiple solid tumors that are resistant to standard immunotherapy.

## Materials and Methods

### Reagents

Reagents information can be found in Appendix Table S1.

### Mice

C57BL/6 male mice (aged 6–8 weeks) were purchased from China Wushi, Inc. (Shanghai, China). STING^−/−^ mice and TLR9^−/−^ mice (both on a C57BL/6 genetic background) were provided by Cyagen Biosciences (Suzhou, China). The mice were housed in a temperature‐ and humidity‐controlled animal facility with a 12‐h light/dark cycle. They had ad libitum access to food and water. All animal procedures were performed in strict accordance with the “National Animal Management Regulations of China” and approved by the Animal Ethics Committee of Mengchao Hepatobiliary Hospital of Fujian Medical University (MCHH‐AEC‐2022‐02).

### Cell lines

Hepa 1‐6 cells (mouse liver cancer cells), B16‐F10 cells (mouse melanoma cells), NIH/3T3 cells (mouse embryonic fibroblast cells), and 293FT cells (human embryonic kidney cell line) were supplied by American Type Culture Collection (ATCC, Manassas, VA). These cells were cultured and maintained in DMEM (containing 10% fetal bovine serum (FBS) and 100 IU/ml penicillin/streptomycin, Gibco, UA), and their supernatants were tested with the TransDetecte PCR Mycoplasma Detection Kit (TransGen Biotech, Beijing, China) to confirm the absence of mycoplasma infection. The Hepa 1‐6 cells, serving as the primary focus of this study, underwent additional authentication through multiplex PCR amplification of 18 short tandem repeat (STR) loci. The authentication report, provided by Genetic Testing Biotechnology Co., Ltd. (Suzhou, China), confirmed a 96.00% match to ExPASy cell line Hepa 1‐6. BMDCs were isolated from the bone marrow of 6–8 weekly aged C57BL/6 mice according to established methods (Wu *et al*, 2020), and then cultured in RPMI‐1640 (Gibco, USA) containing 10% FBS and 1% penicillin/streptomycin.

### Construction of pDNA encoding neoantigen or OVA_257_ _–264_

Hepa 1‐6 tumor cell‐specific neoantigen oligonucleotide (GGCCGTATTGGCCCCGCCACCTGTGAGCGGGATGAAGGCCCGAAACTACCTGCAGTTTCTGCCCTCGAAAACCAAGGTGGCTTAAGGCCAAACAGGCC) and OVA_257–264_ oligonucleotide (GGCCGTATTGGC CCCGCCACCTGTGAGCGGGAGCATCATCAACTTCGAGAAGCTGTAAGGCCAAACAGGCC) were synthesized and amplified using T3_SfiI (AGTGATTTCCGGCCTGTTTGGCC) and T7_SfiI (CGACTCACTATAGGGCCGTATTGGCC) primers. The PCR product was then purified using the MinElute kit and further digested with SfiI. Meanwhile, the plasmid PresentER cassette was also digested with SfiI and treated with calf intestinal phosphatase. Then, the digested PCR product and digested vector were purified with MinElute kit and ligated with T4 ligase to form neoantigen or OVA PresentER vector and further transformed into Stbl3 competent cells for plasmid preparation.

### Synthesis of PEI_25000_‐C_14_

PEI_25000_‐C_14_ was synthesized by reacting 1, 2‐epoxytetradecane and PEI_25000_ in absolute ethanol at a mass ratio of 1:2 at 90°C for 48 h. After cooling down to room temperature, the product was purified by precipitation in cold anhydrous ether.

### Preparation and characterization of DNA nanovaccines (pDNA‐NPs)

The pDNA‐NPs were prepared by the double emulsion solvent evaporation method (w/o/w). Briefly, 400 μl of diethylpyrocarbonate (DEPC) water containing 100 μg of plasmid DNA was added to 1 ml of dichloromethane containing PEI_25000_‐C_14_ (2 mg) and PLGA (10 mg). The above mixture was sonicated for 2 min at 60 W in an ice bath to form a primary W/O emulsion. Then, 3 ml DEPC water was added to the primary emulsion and further emulsified by ultrasound for 2 min at 60 W to form a secondary W/O/W emulsion. Subsequently, the dichloromethane was removed by a rotary evaporator (RE‐52AA, Shanghai), and the pDNA‐NPs were finally obtained. The morphology and size of the nanoparticles were characterized by scanning electron microscopy (SEM, Nova NanoSEM 230, USA) and transmission electron microscopy (TEM, JEOL, Japan). Nano ZS (Malvern Instruments, Malvern, UK) was used to investigate the hydrodynamic size (DLS) and zeta potential of pDNA‐NPs.

### *In vitro* pDNA release

To investigate the plasmid DNA release from nanovaccines *in vitro*, pDNA‐NPs (3.56 mg/ml) were respectively dispersed in 1 ml PBS buffer with pH 5.0 and 7.4 and incubated in a shaker at 37°C. Then, 0.5 ml supernatant was extracted after centrifugation at 13,800 *g* for 10 min at a predetermined time point, and 0.5 ml PBS buffer was added to keep the volume constant. The release of plasmid was evaluated by Nano Drop spectrophotometers (ND‐2000, USA).

### Cytotoxicity studies

BMDCs were seeded in 96‐well plates at a density of 2 × 10^4^ cells per well, and NIH/3T3 and Hepa 1‐6 cells were seeded in 96‐well plates at a density of 5 × 10^3^ cells per well, and incubated for 24 h at 37°C. Then, the culture medium containing different concentrations of DNA nanovaccines (0, 5, 10, 15, 20, 25, 30, and 35 μg/ml) was added to 96‐well plates, and the incubation was continued for 24 h. Afterward, the cells were washed with PBS, and 100 μl CCK‐8 working solution (containing 10 μl CCK‐8 solution and 90 μl medium) was added. Finally, the cell viability was measured by CCK‐8 assay.

### Internalization of DNA nanovaccines (pDNA‐NPs) to activate BMDCs

The BMDCs were isolated from the bone marrow of male C57BL/6 mice aged 6–8 weeks and cultured in RPMI 1640 medium containing 20 ng/ml murine GM‐CSF (R&D systems, 415‐ML‐020/CF), 10% FBS, and 10 ng/ml IL‐4 (404‐ML‐010/CF). Internalization of DNA nanovaccines by BMDCs was assessed by flow cytometry and CLSM. Immature BMDCs were seeded in confocal dishes at a density of 1 × 10^6^ cells per well and incubated overnight at 37°C, and the adhered BMDCs were co‐incubated with YOYO‐3‐labeled free pDNA and pDNA‐NPs for 2, 4, and 6 h, and the uptake of pDNA by BMDCs was observed by confocal laser scanning microscopy (CLSM, LSM 780, USA). Meanwhile, the cellular uptake was also quantified by flow cytometry (BD FACSAria TM III, USA). To further investigate the gene transfection in BMDCs, immature BMDCs (1 × 10^6^ cells per well) were seeded into a confocal dish, and the adhered BMDCs were treated with pDNA and pDNA‐NPs for 48 h and then observed by CLSM to monitor the expression of mCherry.

To explore the ability of DNA nanovaccines to stimulate BMDC maturation *in vitro*, immature BMDCs (5 × 10^5^ cells per well) were inoculated in a 24‐well plate and treated with pure NPs, pDNA, pDNA‐NPs, and LPS at 37°C for 24 h. The cells were collected by centrifugation at 800 *g* for 5 min, and then the BMDCs were stained with antibodies of anti‐CD11c‐APC, anti‐CD80‐PE, and anti‐CD86‐Cy7 for 30 min in the dark. Finally, the cells were analyzed by flow cytometry.

### Isolation of RBCs

Whole blood from healthy male C57BL/6 mice obtained through the eyeballs was collected using a heparin sodium anticoagulant tube. The collected whole blood was centrifuged at 1,000 *g* for 10 min at 4°C, and then the serum and buffy‐coat layers containing white blood cells were removed from the red blood cell compartment. The detached RBCs were gently resuspended in cold 1 × PBS and centrifuged at 500 *g* for 15 min at 4°C. The above procedure was repeated twice, and the final solution was gently resuspended in 10 ml cold 1 × PBS and stored at 4°C. The above‐mentioned solution was referred to as the RBC stock solution.

### Attachment of the DNA nanovaccines to RBC

The DNA nanovaccines were mixed with an equal volume of 1 × 10^8^ red blood cells in an Axygen 2.0 ml Self‐Standing Screw Cap Tubes, and the tubes were evenly rotated on a silent mixer (WH‐986, Kylin‐Bell, China) at a speed of 5 rpm for 1 h. The hitchhiked RBCs were centrifuged at 100 *g* for 5 min at 4°C and washed twice with 1 mL of cold PBS. The hitchhiked RBCs were finally resuspended in 500 μl of cold PBS and used for further characterization. To determine the binding efficiency, DNA nanovaccines were prelabeled with DIO to prepare RBC‐hitchhiking DNA nanovaccines, then lysed with deionized water for the fluorescence intensity measurement. Meanwhile, the RBC‐hitchhiking DNA nanovaccines were further tested by flow cytometry (BD, FACSVerse), CLSM (Zeiss LSM780), and SEM (Regulus SU8100). For SEM measurement, the RBC‐hitchhiking DNA nanovaccines were fixed with 2.5% glutaraldehyde solution for 1 h at room temperature, then dehydrated with a series of gradient alcohol (30, 40, 50, 60, 60, 70, 80, 90, and 100%) incubated at each concentration for 10 min, and finally dried naturally.

### *In vivo* biodistribution

RBC‐Nanovaccines obtained at different Nanovaccine‐to‐RBC ratios (10:1, 50:1, 100:1, and 250:1) were intravenously injected into C57BL/6 mice. After 24 h of injection, the mice were euthanized and the major organs (heart, liver, spleen, lung, and kidney) of the mice were excised, and the fluorescence intensity of DiI in the major organs was observed with an imaging system (ChemiDoc™MP, Bio‐Rad).

### Expression of neoantigen in spleen tissue

Male C57BL/6 mice of 22–25 g were randomly divided into different groups, which received intravenous injection of PBS, Nanovaccines (containing 10 μg pDNA), and RBC‐Nanovaccines at a Nanovaccine‐to‐RBC ratio of 100:1 (containing 10 μg pDNA), respectively. And 48 h after injection, the mice were euthanized and the spleen tissues were collected. Subsequently, the fluorescence intensity of the spleen tissues in each group was analyzed with an imaging system (ChemiDoc™MP, Bio‐Rad).

In addition, the freshly collected mouse spleen of each group was placed in an embedding box and then the OCT was added to immerse tissue. Subsequently, the bottom of the embedding box was slightly in contract with liquid N_2_ for 3–5 min until the spleen tissue was completely frozen. Afterward, the spleen tissue was sectioned into 5 μm thick slices using a cryostat (Leica CM 1950, IL, USA), followed by immersion in acetone for 5–10 min. The fixed sectioned tissues were then washed three times with 1 × PBS and stained with DAPI (Ex/Em, 340/488 nm, 1 mg/ml) for 30 min at room temperature. Finally, the fluorescence signal of the mCherry tag (Ex/Em, 587/610 nm) in the spleen was observed by CLSM.

### Flow cytometry analysis for immune cells

Single‐cell suspensions were mainly derived from peripheral blood, spleen, liver, and tumor tissues. Briefly, the peripheral blood collected from the eyeballs was transferred into a heparin sodium anticoagulant tube, then lysed with red blood cell lysate for 3 min, and washed with PBS three times. To obtain splenocytes, the spleen was added into six‐well plates with 1 ml of RPMI 1640 medium and blown evenly with a sterile syringe until the medium became turbid, and the medium containing cells was centrifuged by a Ficoll‐Paque™ PREMIUM sterile solution at 800 *g* for 30 min at 4°C, and then the single‐cell suspensions were washed with PBS three times. However, tumor and liver tissue were digested in RPMI 1640 medium containing type collagenase type IV (Sigma, 1 mg/ml), hyaluronidase (Sigma, 0.2 mg/ml), and deoxynuclease I (Gibco, 0.02 mg/ml) at 37°C for 1 h until the medium was turbid. Then, the above medium was passed through a 40 μm cell strainer, and the cells were collected by centrifugation at 800 *g* for 5 min. The above different sourced single‐cell suspensions were stained with corresponding antibodies listed below for 30 min at 4°C and then washed, centrifuged, resuspended, and finally analyzed by BD FACS II (BD LSR Analyzer II, CA, USA). Notably, for the staining of intracellular markers, such as IFN‐γ, the cells should be permeabilized using Cytofix/Cytoperm Plus (BD Biosciences) before the addition of antibody.

The fluorochrome antibodies used for immunostaining have good specificity for CD11c (eBioscienceTM, Cat no: 17‐0114‐82, Clone: N418, 1:100 dilution), CD80 (eBioscienceTM, Cat no: 12‐0801‐82, Clone: 16‐10A1, 1:100 dilution), CD86 (eBioscienceTM, Cat no: 25‐0862‐82, Clone: GL1, 1:100 dilution), MHC I (eBioscienceTM, Cat no: 11‐5958‐82, Clone: AF6‐88.5.5.3, 1:50 dilution), CD3 (eBioscienceTM, Cat no: 17‐0032‐82, Clone: 17A2, 1:100 dilution), CD3 (eBioscienceTM, Cat no: 11‐0032‐82, Clone: 17A2, 1:100 dilution), CD45 (eBioscienceTM, Cat no: 17‐0451‐82, Clone: 30‐F11, 1:100 dilution), CD8a (eBioscienceTM, Cat no: 12‐0081‐82, Clone: 53‐6.7, 1:100), CD8 (MBL, Cat no: D271‐4, Clone: KT15, 1:100 dilution), CD4 (Biolegend, Cat no: 100432, Clone: GK1.5, 1:100 dilution), CD4 (eBioscienceTM, Cat no: 11‐0042‐85, Clone: RM4‐5, 1:100 dilution), CD44 (eBioscienceTM, Cat no: 25‐0441‐82, Clone: IM7, 1:100 dilution), CD62L (eBioscienceTM, Cat no: 45‐0621‐82, Clone: MEL‐14, 1:100 dilution), IFN‐γ (eBioscienceTM, Cat no: 25‐7311‐82, Clone: XMG1.2, 1:100 dilution), and OVA_257–264_ (SIINFEKL) peptide bound to H‐2K^b^ (eBioscienceTM, Cat no: 12‐5743‐82, Clone: 25‐D1.16, 1:50 dilution). H‐2K^b^/OVA (SIINFEKL) tetramer was generated using the MBL Quickswitch Quant H‐2 Kb Tetramer Kit‐PE (Cat no: TB‐7400‐K1, 1:10 dilution) according to the manufacturer's protocol. The stained cells were examined by flow cytometry, and all antibodies were used according to the manufacturer's instructions. The results were analyzed by FlowJo software packages.

### Assessment of immune responses elicited by RBC‐Nanovaccines

To investigate the effect of different dosage forms and different immunization times on the immune response of mice, C57BL/6 mice were randomly divided into PBS group, Nanovaccine group, RBC‐Nanovaccine group, and RBC‐Nanovaccine^1^ group. On the 7th day, 200 μl of PBS, Nanovaccines, and RBC‐Nanovaccines (containing 500 μg nanovaccines) were injected intravenously (i.v.), and the same dose was injected on the 14th day except for RBC‐Nanovaccine^1^ group with only one immunization to induce a specific immune response in mice. The spleen was collected on day 18 and processed into a single‐cell suspension for flow cytometry analysis as described above.

### Prophylactic experiments

To examine the specificity of neoantigen DNA vaccine for preventing Hepa 1‐6 tumor growth, C57BL/6 mice were randomly divided into four groups: PBS group, Nanovaccine group, RBC‐Nanovaccine group, and RBC‐Nanovaccine^1^ group, and mice in each group were vaccinated as described above. On day 0, 1 × 10^6^ Hepa 1‐6 or B16‐F10 tumor cells were injected into the groin of mice to establish the Hepa 1‐6 or B16‐F10 prophylactic model. Starting from day 7, the body weight and tumor volume (V) of mice were monitored every 2 days, and digital pictures of mice in each group were taken when the tumor volume of one of the mice reached 1,500 mm^3^. The tumor volume was calculated as *V* = Length (mm) × Width (mm)^2^/2. The survival time was the time interval from the start of tumor inoculation to death or when the tumor volume exceeds 1,500 mm^3^.

### Therapeutic experiments

The elimination efficiency of as‐prepared RBC‐Nanovaccines on the established Hepa 1‐6 tumor model was investigated as below. In brief, 2 × 10^6^ Hepa 1‐6 cells were injected into the right groin on day 0. When the Hepa 1‐6 tumor volume reached 50 mm^3^, the mice were divided into PBS group (G1), Nanovaccine (G2) group, and RBC‐Nanovaccine group (G3). On days 0, 4, and 8, the mice were treated with PBS, Nanovaccines, and RBC‐Nanovaccines (containing 500 μg nanovaccines). In addition, one cohort of mice receiving RBC‐Nanovaccines was supplemented with intravenous injection of anti‐PD‐1 (2.5 mg/ml, Biolegend, Cat no: 135234, Clone: 29F.1A12) on days 2, 6, and 10. The tumor volume, body weight, and survival time of mice were monitored as described above.

The tumors and major organs of the mice were collected on the 24th day to evaluate the immune response in the mice after different treatments, and the tumors in each group were made into a single‐cell suspension for flow cytometry analysis according to the above method.

### Re‐challenge studies

To assess the effect of RBC‐Nanovaccines combined with anti‐PD‐1 on tumor recurrence, the Hepa 1‐6 tumor‐bearing mice were treated as described above, and the peripheral blood of each group of mice was collected on the 24th day. Then, the peripheral blood was processed into a single‐cell suspension for flow cytometry analysis as described above.

Subsequently, the cured mice were re‐inoculated with 1 × 10^6^ Hepa 1‐6 cells on day 60, and the untreated mice of the same age were designated as naïve group. The tumor volume, body weight, and survival time of mice were monitored.

### Orthotopic HCC tumor model

To study the effect of different vaccination routes on the treatment of orthotopic Hepa 1‐6 tumor, 5 × 10^5^ Luc‐Hepa 1‐6 cells (25 μl) were mixed with an equal volume of Matrigel suspension and slowly injected into the liver lobe by surgical operation. On day 10, the mice were injected intraperitoneally with 200 μl XenoLight‐D‐luciferin (15 mg/ml) to confirm the establishment of the orthotopic Hepa 1‐6 tumor model through bioluminescence imaging (PerkinElmer *IVIS* Spectrum, MA, USA). Afterward, the mice were randomly assigned to three groups: PBS, RBC‐Nanovaccines (s.c.) + anti‐PD‐1 (i.v.), and RBC‐Nanovaccines (i.v.) + anti‐PD‐1 (i.v.) groups. The mice in each group were treated with the indicated formula. Subsequently, orthotopic Hepa 1‐6 tumor growth was monitored by *in vivo* imaging, and the average radiance (bioluminescent signals, photons, s^−1^ cm^−2^ sr^−1^) was quantified using *IVIS* Living Image 4.2 software.

### *In vivo* depletion of T cells

To further assess the role of T‐cell populations in preventing tumorigenesis, C57BL/6 mice were intraperitoneally injected with 100 μg of anti‐CD4 antibody (BioXCell, Cat no: BE0003‐1, Clone: GK1.5) or anti‐CD8 antibody (BioXCell, Cat no: BE0117, Clone: YTS 169.4) on 15, 11, 8, 4, and 1 days before tumor inoculation, and the percentage of T cells in peripheral blood was analyzed by cytometry to ensure the depletion of T‐cell subsets during the experimental period. One group of mice treated with IgG isotype (BioXCell, Cat no: BE0090, Clone: LTF‐2) served as controls.

### *In vivo* safety assessment

C57BL/6 mice were intravenously injected with PBS and RBC‐Nanovaccines twice with an interval of 7 days. After 4 days of the second injection, the liver and serum of the mice in each group were collected. Liver samples were fixed with 4% paraformaldehyde solution for 24 h and then sliced into 5 μm thick tissue sections using a cryostat (Leica RM 2235, IL, USA) for histological observation. The serum was analyzed using an automated hematology analyzer.

The potential immune response in the liver tissues was further evaluated. Briefly, liver tissues of each group were prepared into single‐cell suspensions, followed by staining with antibodies of anti‐CD3‐APC and anti‐CD8‐PE for 30 min at 4°C and analyzed by flow cytometry.

### ELISPOT assay

IFN‐γ secreted by neoantigen‐specific T cells generated by RBC‐Nanovaccine inoculated mice was detected by ELISPOT kit (Mabtech, 3321‐4APT‐10). Briefly, C57BL/6 mice were injected intravenously with RBC‐Nanovaccines (containing 500 μg nanovaccines) on the days 7 and 14, respectively. Four days after the vaccination, the spleens of the mice were collected and T cells were extracted from the spleens as described above. At the same time, the immature BMDCs were collected by the above method and incubated with neoantigen peptide (4 μg) for 48 h. Then, 3 × 10^4^ matured BMDCs were co‐incubated with 3 × 10^5^ T cells in the ELISPOT plate for 48 h. After the incubation, the plate was washed with PBS and then incubated with R4‐6A2‐biotin antibody (1 μl/ml, 100 μl per well) for 2 h at room temperature. The plate was then washed again, and streptavidin ALP (1:1,000 dilution, 100 μl per well) was further added to the plate and incubated for 1 h at room temperature, followed by incubation with 3, 3′, 5, 5′‐Tetramethylbenzidine (TMB) substrate solution for 5–8 min at room temperature. Finally, the IFN‐γ spot‐forming was observed and analyzed by ELISPOT Analysis System (AT‐Spot‐2200, Beijing Antai Yongxin Medical Technology Co., Ltd).

### RNA sequencing and bioinformatic analysis

The tumor tissues of the PBS treated group (G1) and the RBC‐Nanovaccines + anti‐PD‐1 treated group (G5) were extracted and quickly frozen in liquid N_2_. Subsequently, total RNA was extracted using Triol reagent kit according to the manufacturer's protocol. After extraction of total RNA, mRNA was further enriched and fragmented, and cDNA was obtained by reverse transcription. Then, transcriptome libraries were further constructed according to the manufacturer's instructions and sequenced by the Illumina novaseq6000 platform (paired end, 150 bp). All qualified raw reads were aligned to the mouse genome (mm10, https://www.gencodegenes.org) using STAR (Dobin *et al*, 2012), and then expression levels of genes were further quantified using FPKM (transcripts per million) value. Differential expression analysis between different groups was conducted by limma package (Ritchie *et al*, 2015), and KEGG pathway enrichment analysis was conducted with clusterProfiler package using differentially expressed genes (|log_2_ fold change| > 1, *P*‐value < 0.05). The infiltration level of each immune cell type was inferred by CIBERSORT (Newman *et al*, 2015).

### Cytokine assay

To evaluate the systemic immune response elicited by different vaccine formulations, the blood was extracted from the prophylactic model mice and centrifuged at 900 *g* for 10 min to obtain serum. Subsequently, the concentrations of cytokines in the serum of each group were detected using an ELISA kit (TNF‐α, Boster, EK0527; IFN‐γ, Boster, EK0375; CRP, Boster, EK0977) according to the manufacturer's instructions.

For analysis of cytokines in tumors, the tumors after different treatments were isolated at the day 24, and 50 mg of tumors in each group was weighed and placed in PBS (500 μl) containing PMSF and protease inhibitor cocktail (MedChemExpress, China). Tumor tissues were then homogenized and centrifuged at 9,600 *g* for 10 min. Afterward, the supernatants were collected and the concentrations of cytokines in the tumors of each group were detected using an ELISA kit (TNF‐α, Boster, EK0527; IFN‐γ, Boster, EK0375; TGF‐β, Boster, EK0515; IL‐10, Boster, EK0417; IL‐12, Boster, EK0422) according to the manufacturer's instruction.

### Statistical analysis

Animals were randomized into different treatment groups. The experiments were conducted as nonblind test. No mice were excluded from all the experiments. The statistical analysis of all data was analyzed by one‐way analysis of variance (ANOVA) or unpaired two‐tailed Student's *t*‐test through using GraphPad Prism 8.0 software. The difference between groups was considered statistically significant at *P* < 0.05, *P < 0.05, **P < 0.01, and ***P < 0.001.

## Author contributions

**Ming Wu:** Conceptualization; formal analysis; investigation; writing – original draft; writing – review and editing. **Zijin Luo:** Formal analysis; validation; investigation; visualization; methodology. **Zhixiong Cai:** Conceptualization; methodology. **Qianqian Mao:** Investigation; methodology. **Zhenli Li:** Software; validation; methodology. **Hao Li:** Validation; methodology. **Cao Zhang:** Validation; investigation. **Yuting Zhang:** Validation; methodology. **Aoxue Zhong:** Validation; methodology. **Liming Wu:** Resources; project administration; writing – review and editing. **Xiaolong Liu:** Conceptualization; Resources; project administration; writing – review and editing.

## Disclosure and competing interests statement

The authors declare that they have no conflict of interest.

## Supporting information



Appendix

Expanded View Figures PDF

PDF+

Source Data for Figure 1

Source Data for Figure 2

Source Data for Figure 3

Source Data for Figure 4

Source Data for Figure 5

Source Data for Figure 6

Source Data for Figure 7

## Data Availability

Data are available in a public, open access repository. The raw sequencing data in this article have been deposited at Genome Sequencing Achieve database (GSA, https://ngdc.cncb.ac.cn/gsa) under the accession number CRA011425.
