# Supplementary material for: Spleen‐targeted neoantigen DNA vaccine for personalized immunotherapy of hepatocellular carcinoma
Source: EMBO Mol Med. 2023 Aug 8;15(10):e16836. doi: 10.15252/emmm.202216836 (PMC10565630; doi:10.15252/emmm.202216836)
Supplement: Supplementary file 1 — Appendix [file EMMM-15-e16836-s005.pdf]

Appendix Data

Table of contents:

APPENDIX FIGURES	PAGE
Appendix Figure S1	2
Appendix Figure S2	2
Appendix Figure S3	3
Appendix Figure S4	3
Appendix Figure S5	3
Appendix Figure S6	4
Appendix Figure S7	4
Appendix Figure S8	5
Appendix Figure S9	5
Appendix Figure S10	6
Appendix Figure S11	6
Appendix Figure S12	7
Appendix Figure S13	7
Appendix Figure S14	7
Appendix Figure S15	8
Appendix Figure S16	8
Appendix Figure S17	9
Appendix Figure S18	9
Appendix Figure S19	10
Appendix Figure S20	10
Appendix Figure S21	10
Appendix Figure S22	11
Appendix Figure S23	11
Appendix Figure S24	12
Appendix Figure S25	12
Appendix Figure S26	13
Appendix Table S1	14

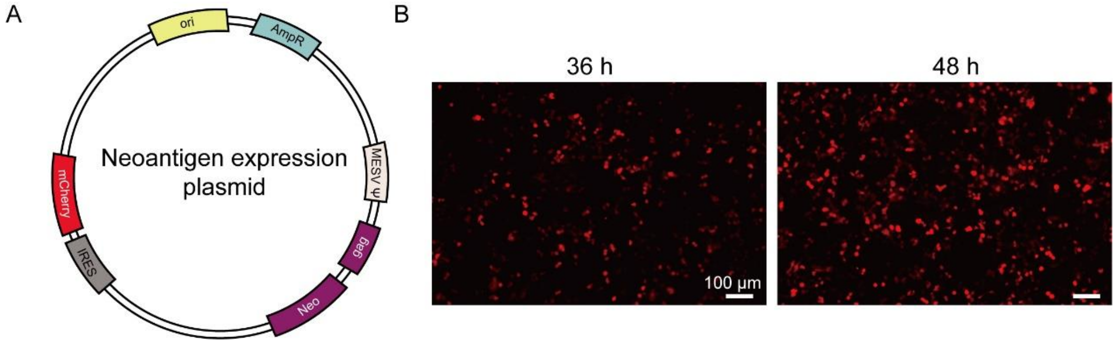

Appendix Figure S1. Construction of pDNA encoding Hepa1-6 liver cancer cell-specific neoantigen. (A) Expression vector of pDNA carrying the cassettes for neoantigen (Neo) and mCherry. (B) Fluorescence microscope image of pDNA transfected in 293FT cells by Lipofectamine 3000 (a commercial liposome reagent often used for nucleic acid transfection) for 36 h and 48 h.

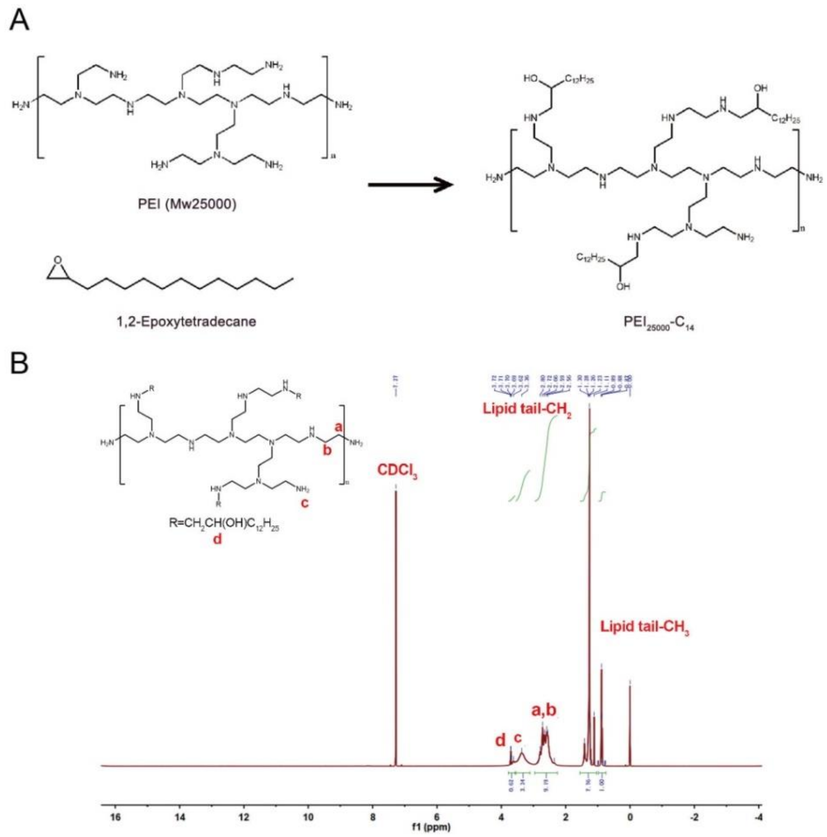

Appendix Figure S2. Synthesis of PEI_{25000}-C_{14}. (A) Synthesis route of PEI_{25000}-C_{14}. (B) The ^{1}HNMR spectrum of PEI_{25000}-C_{14} in $CDCl_3$.

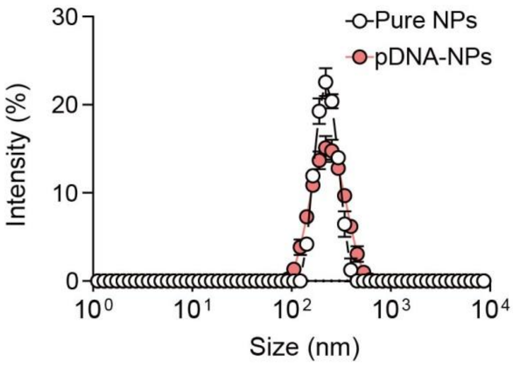

Appendix Figure S3. Hydrodynamic size distribution of pure NPs and pDNA-NPs measured by DLS (n = 3 biological replicates).

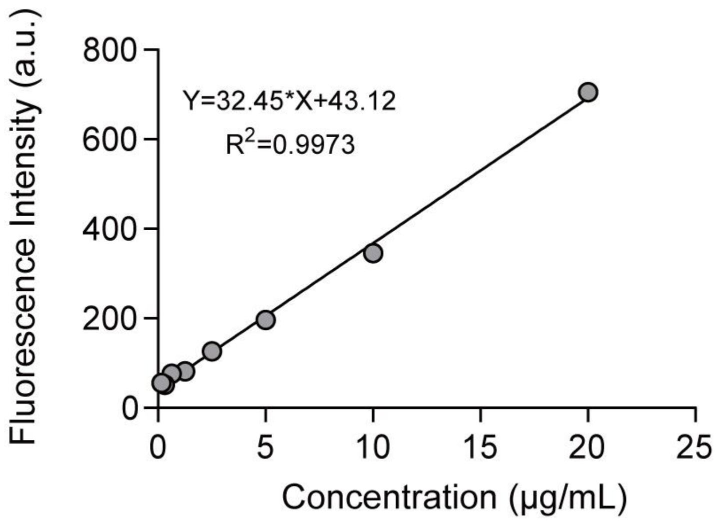

Appendix Figure S4. The standard curve of YOYO-3 labeled pDNA determined by fluorescence spectrophotometer. The various concentration of YOYO-3 labeled pDNA (0 mg/mL to 20 mg/mL) was quantified according to the linear fitting (Y = 32.45*X + 43.12, R^2 = 0.9973) of fluorescence intensity at 631 nm.

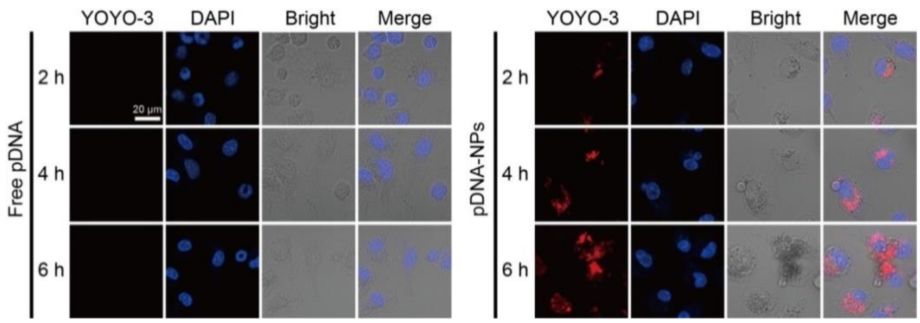

Appendix Figure S5. Internalization of DNA nanovaccines by BMDCs as indicated in Fig 1J. CLSM images of BMDCs treated with free pDNA and pDNA-NPs for 2, 4, and 6 h, respectively. Nucleus was stained with DAPI (blue) and pDNA was labeled with YOYO-3 (red).

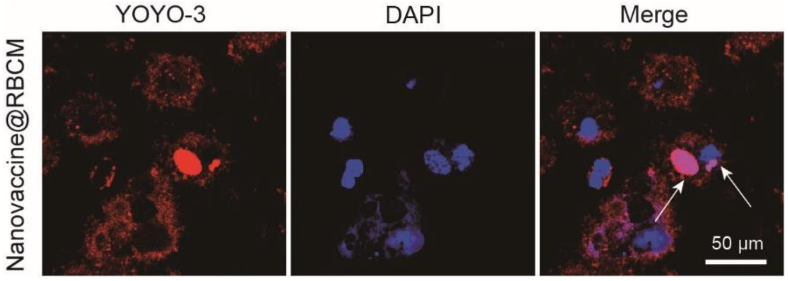

Appendix Figure S6. Internalization of DNA nanovaccines by BMDCs. CLSM images of BMDCs treated with pDNA-NPs for 24 h. Nucleus was stained with DAPI (blue) and pDNA was labeled with YOYO-3 (red). The localization of pDNA on the cell nucleus is indicated by the white arrow.

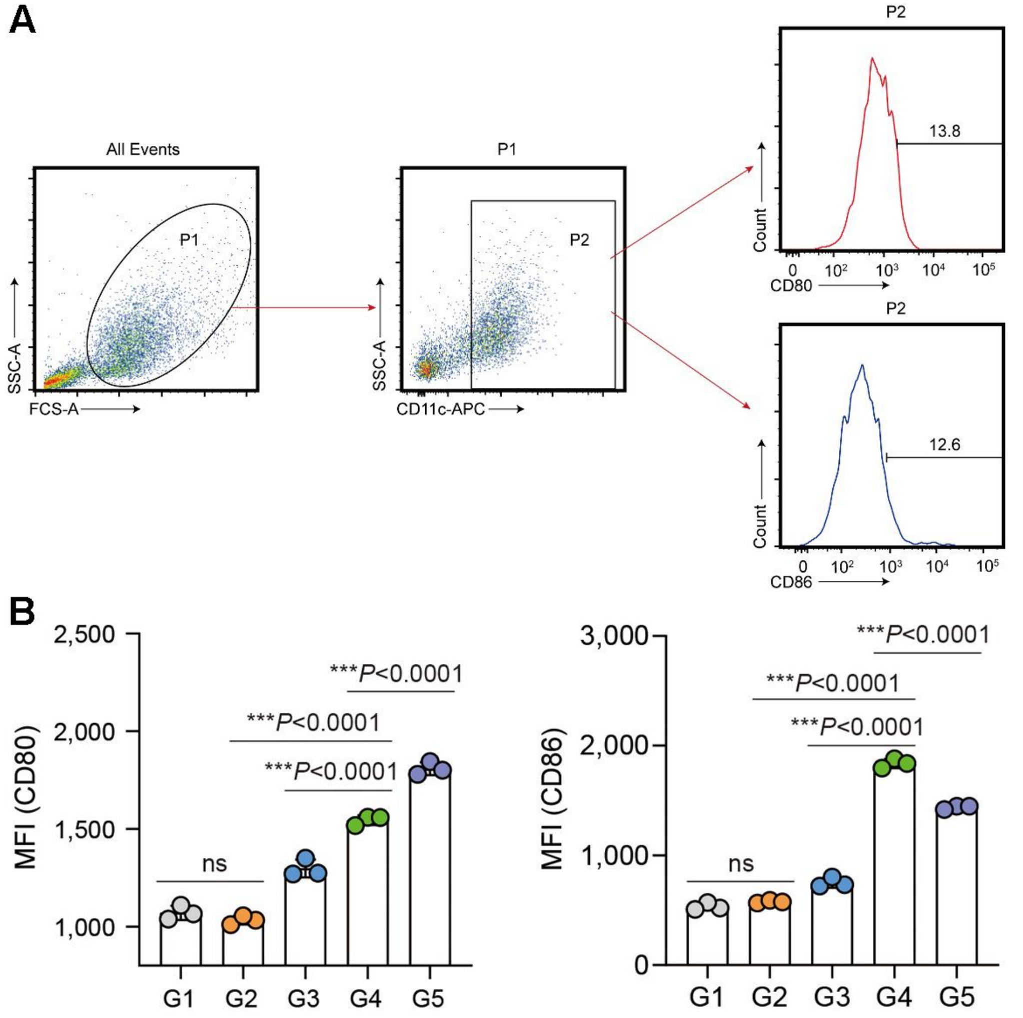

Appendix Figure S7. The expression of CD80 and CD86 of BMDCs incubated different formulations as indicated in Fig 1N and O. (A) Representative flow cytometry gating strategies for experiments in Fig 1N. To detect the maturation of BMDCs after receiving different treatments, BMDCs were stained with anti-CD11c-APC, anti-CD80-PE, and anti-CD86-PE-Cy7 at 4°C for 30 min for cytometry analysis. (B) The statistical analysis results corresponding to Fig 1O (n = 3 biological replicates). Data are presented as mean ± SD. Statistical analyses were conducted by one-way ANOVA with Tukey's multiple comparison test. ***$P < 0.001$. ns means no significant difference.

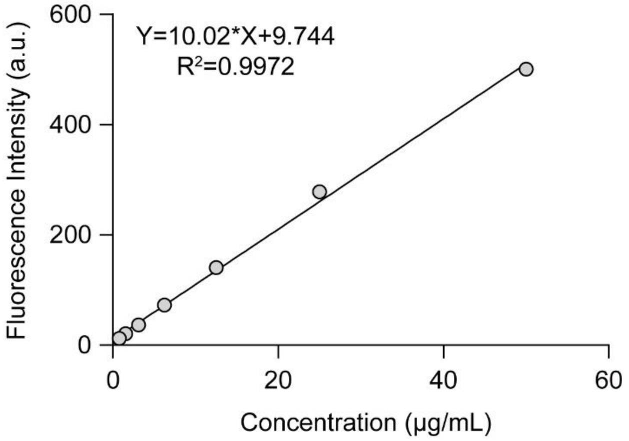

Appendix Figure S8. The standard curve of DIO labeled DNA nanovaccines determined by fluorescence spectrophotometer. The various concentration of DIO labeled DNA nanovaccines (0 mg/mL to 50 mg/mL) was quantified according to the linear fitting (Y = 10.02*X + 9.744, R^2 = 0.9972) of fluorescence intensity at 540 nm.

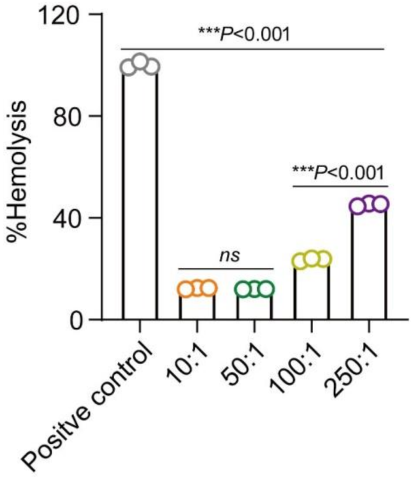

Appendix Figure S9. Hemolysis of RBCs after hitchhiking DNA nanovaccines at different nanovaccine-to-RBC ratios. RBC treated by pure water was used as a positive control (n = 3 biological replicates per group). Data are presented as mean ± SD. Statistical significance was calculated by one-way ANOVA with two-tailed Student's t-test. ***P < 0.001. ns means no significant difference.

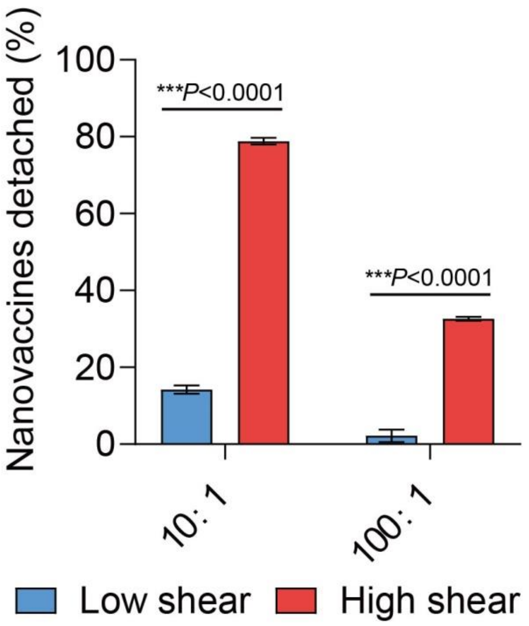

Appendix Figure S10. Effect of shear stress on the detachment of the hitchhiked nanovaccines from RBCs. Data is presented as mean ± SD (n = 3 biological replicates per group). Data are presented as mean ± SD. Statistical significance was calculated by unpaired two-tailed Student’ s t test. ****P* < 0.001.

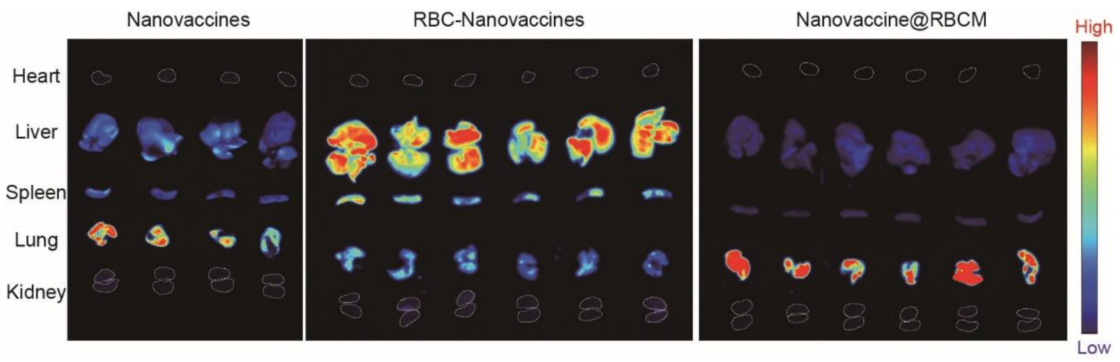

Appendix Figure S11. Biodistribution of different vaccine formulations related to Fig 2H. Ex vivo fluorescence images of major organs were obtained 24 hours after intravenous injection of DiI-labeled DNA nanovaccines that were hitchhiked on red blood cells (RBC-Nanovaccines). These images were compared to those of pure DNA nanovaccines or DNA nanovaccines coated with red blood cell membrane (Nanovaccines@RBCM).

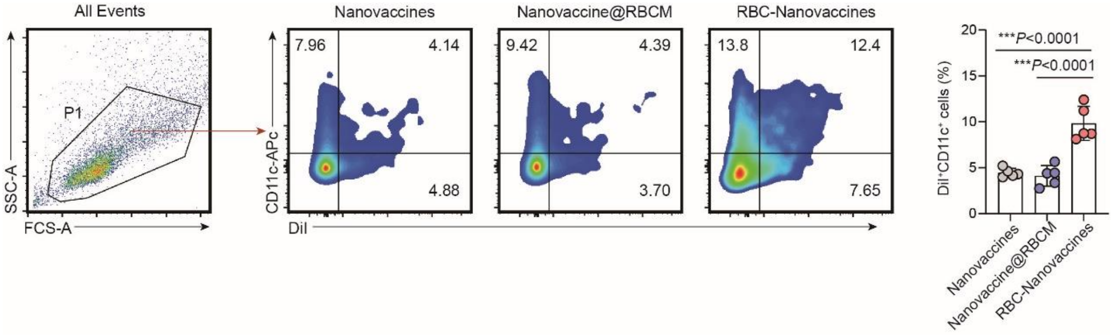

Appendix Figure S12. The internalization of nanovaccines by DC cells in the spleen. Representative flow cytometry plots (left) and quantification (right) are presented (n = 5 animals per group). Data are presented as mean ± SD. Statistical significance was calculated by one-way ANOVA with Tukey's multiple comparison test. ***$P < 0.001$.

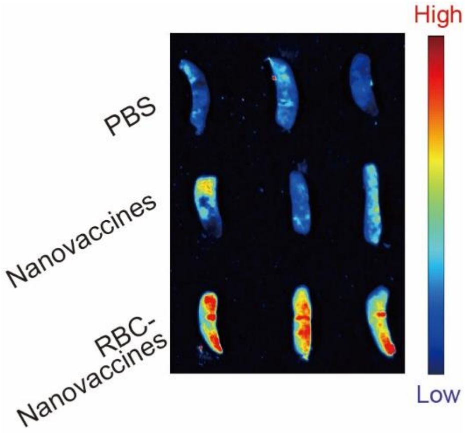

Appendix Figure S13. Fluorescence images related to Fig 2I and J. Each group was set with three biological replicates.

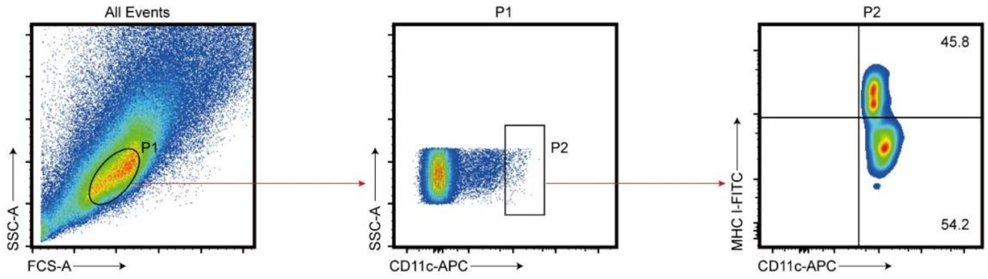

Appendix Figure S14. Representative flow cytometry gating strategies for experiments in Fig 3B. To detect the percentage of MHC I^+CD11c^+ cells in the spleen.

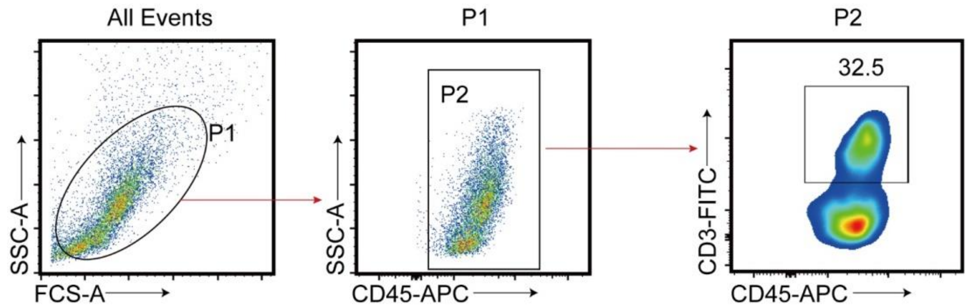

Appendix Figure S15. Representative flow cytometry gating strategies for experiments in Fig 3B. To detect the percentage of $CD45^+CD3^+$ T cells in the spleen.

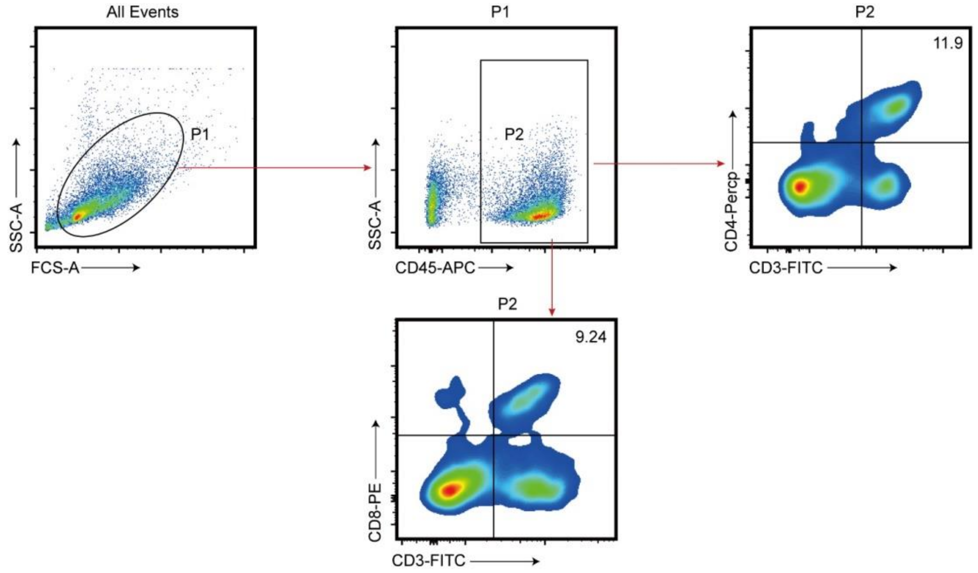

Appendix Figure S16. Representative flow cytometry gating strategies for experiments in Fig 3B. To detect the percentage of $CD45^+CD3^+CD4^+$ T cells and $CD45^+CD3^+CD8^+$ T cells in the spleen.

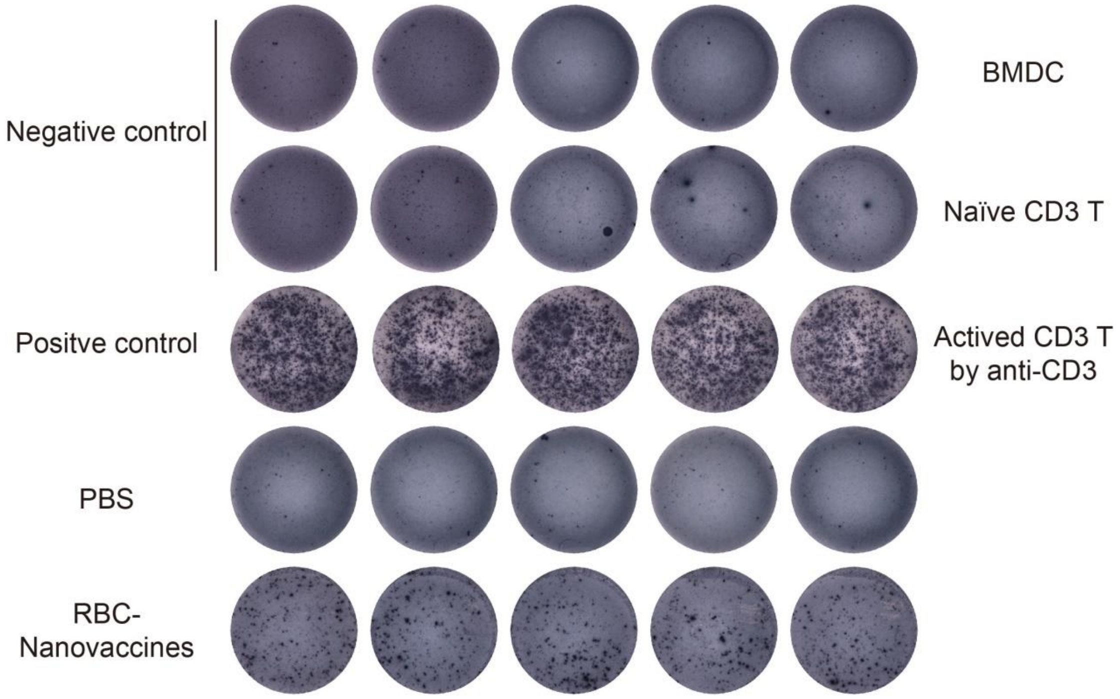

Appendix Figure S17. Activation of neoantigen-specific T cells related to Fig 3E. The spleens of C57BL/6 mice intravenously injected with PBS and RBC-Nanovaccines were excised and analyzed for IFN-γ spot formation by ELISPOT on day 11.

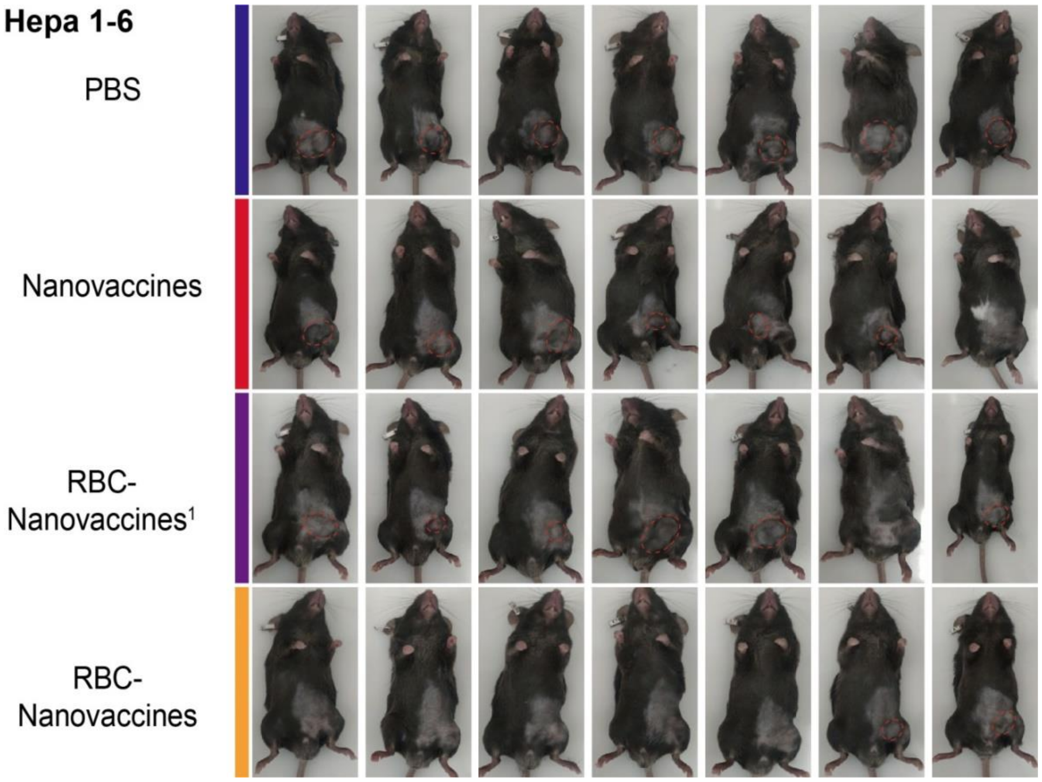

Appendix Figure S18. The digital images of survived mice in each group on the 27th day as indicated in Fig 4D (n = 7 animals per group).

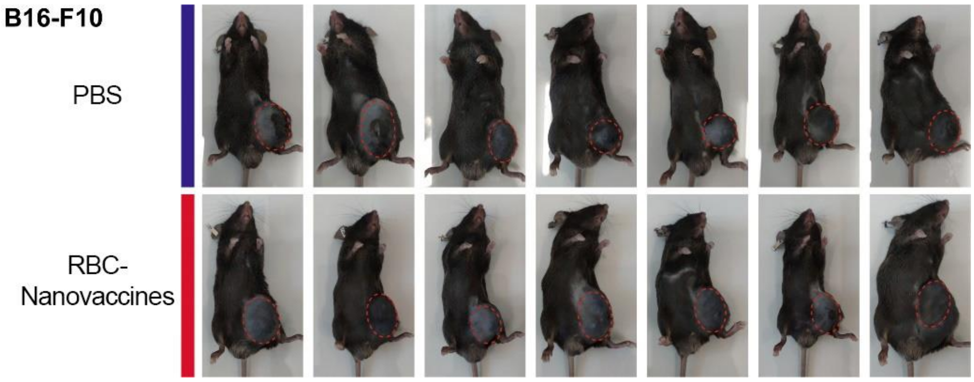

Appendix Figure S19. The digital images of survived mice in each group on the 17th day as indicated in Fig 4L (n = 7 animals per group per group).

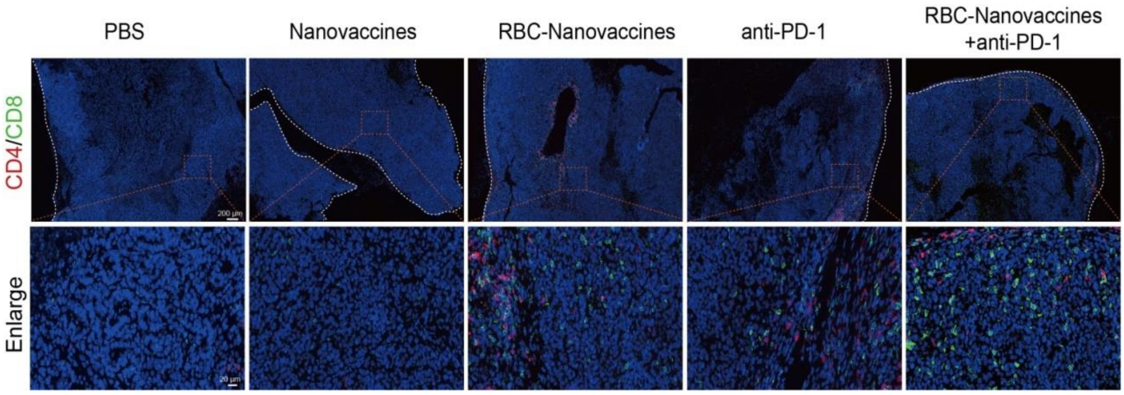

Appendix Figure S20. Immune cells in the tumor after different treatments related to Fig 5H. Immunofluorescence staining images of tumor infiltrated CD4$^+$ T cells (red) and CD8$^+$ T cells (green) on the 24th day after receiving different treatments as indicated. Nucleus were stained with DAPI. The pictures from the lower row are re-used from figure 5H.

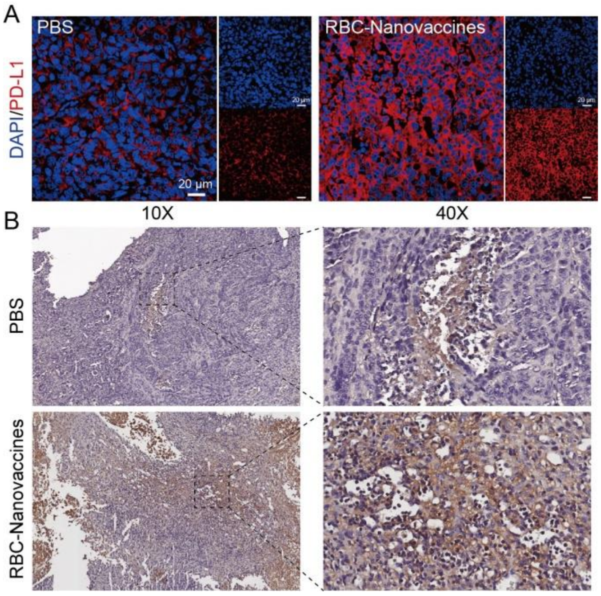

Appendix Figure S21. IF and IHC showing the expression of PD-L1 in tumor tissues.

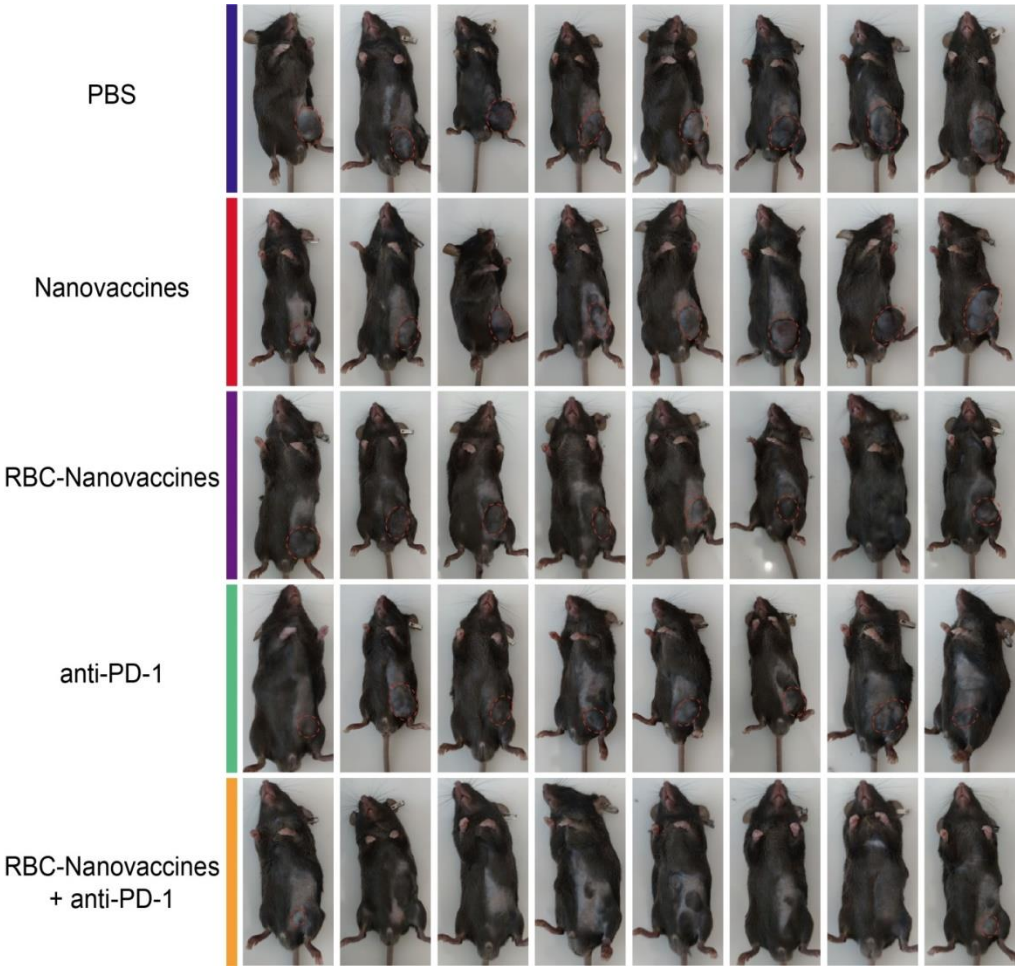

Appendix Figure S22. The digital images of survived mice in each group on the 24th day as indicated in Fig 5F (n = 8 animals per group per group).

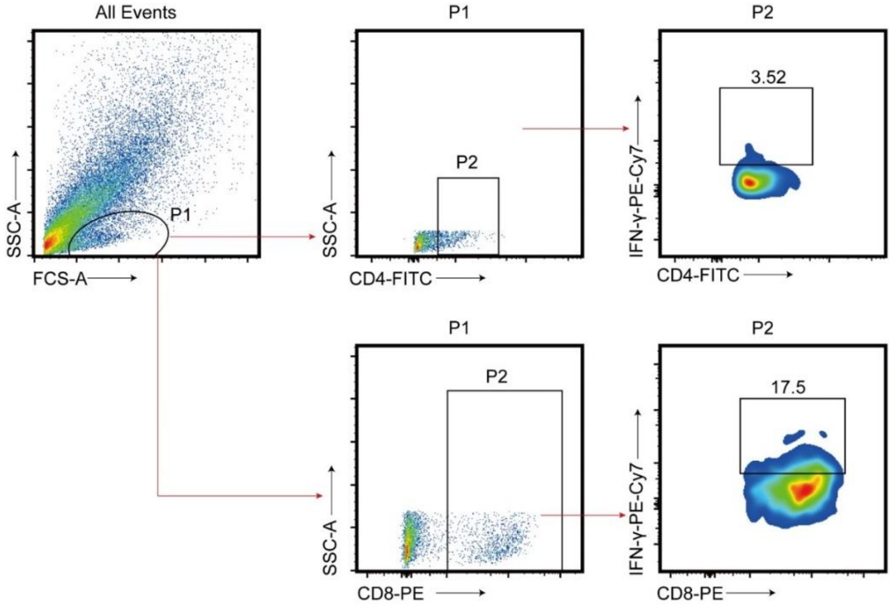

Appendix Figure S23. Representative flow cytometry gating strategies for experiments in Fig 5I. To detect the percentage of IFN-γ^+CD4$^+$ T cells and IFN-γ^+CD8$^+$ T cells in the tumor.

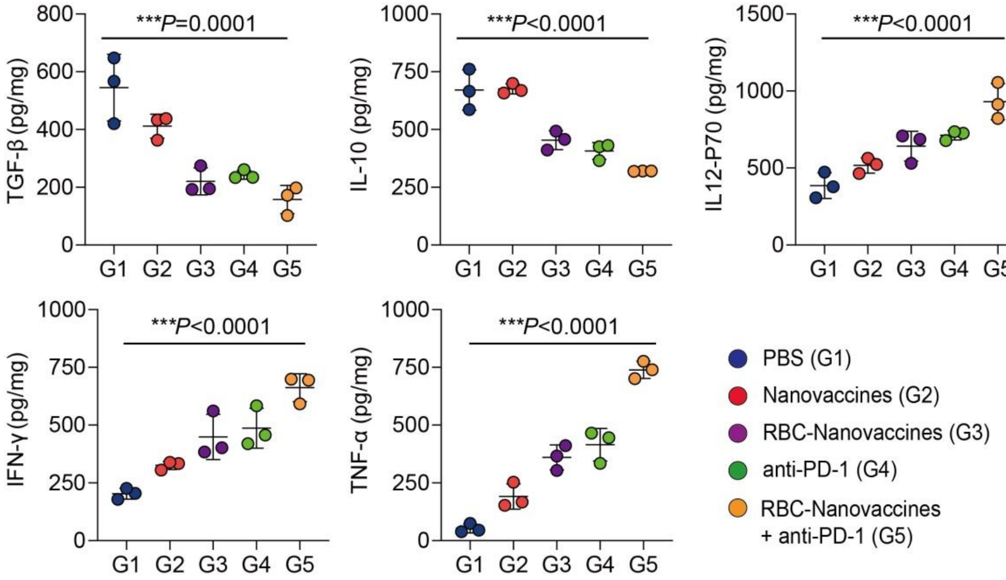

Appendix Figure S24. The levels of cytokines in the tumor after different treatments, which showed the origin data of Fig 5K. The concentration of TGF β, IL-10, IL-12, IFN-γ, and TNF-α in the tumor from mice in the groups of PBS, Nanovaccines, RBC-Nanovaccines, anti-PD-1, and RBC-Nanovaccines+anti-PD-1 analyzed by ELISA (n = 3). Data is presented as mean ± SD. Statistical significance was calculated by one-way ANOVA, ****P* < 0.001.

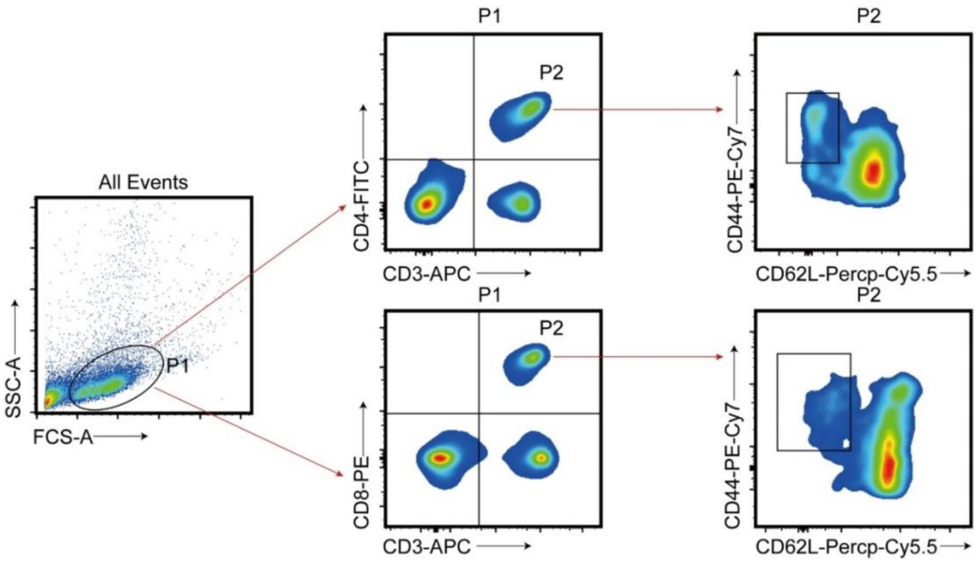

Appendix Figure S25. Representative flow cytometry gating strategies for experiments in Fig 6B. To detect the percentage of CD3$^+$CD4$^+$ T cells, CD3$^+$CD8$^+$ T cells and effector memory T cells (CD4$^+$CD44^{high}CD62L^{low} and CD8$^+$CD44^{high}CD62L^{low}) in the peripheral blood.

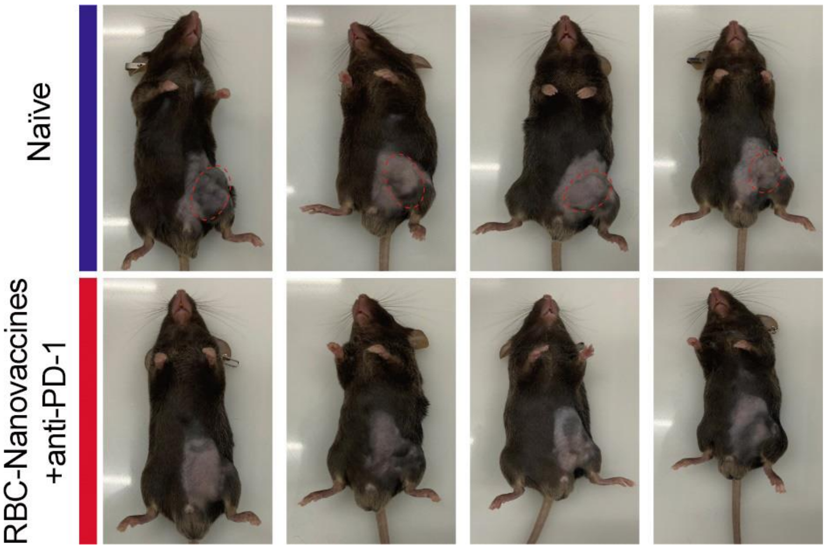

Appendix Figure S26. The digital images of survived mice in each group at the 100th day after subcutaneous rechallenge as indicated in Fig 6G (n = 4 animals per group).

Appendix Table S1. Key reagents information.

REAGENT OR RESOURSE	SOURSE	IDENTIFIER
PLGA	Sigma-Aldrich	Cat# 739944
PEI branched	Sigma-Aldrich	Cat# 408727
1,2-Epoxytetradecane	TCI	Cat# E0314
Dichloromethane	Sinopharm chemical Reagent	Cat# 80047318
Ethanol	Sinopharm chemical Reagent	Cat# 10009164
DAPI (4’, 6-Diamidino-2-phenylindole)	Dojindo Laboratories	Cat# D212
CCK-8 (Cell Counting Kit-8)	Dojindo Laboratories	Cat# CK04
DIO (3,3'-Dioctadecyloxacarbocyanine perchlorate)	Beyotime	Cat# C1038
DiI (1,1'-Dioctadecyl-3,3,3',3' -tetramethylindocarbocyanine perchlorate)	Beyotime	Cat# C1036
Lipopolysaccharide	Sigma-Aldrich	Cat# L3129
D-luciferin, sodium salt	PerkinElmer	Cat# 122799
Mouse IFN-γ Elisa Kit	Boster	Cat# EK0375
Mouse TNF-α Elisa Kit	Boster	Cat# EK0527
Mouse IL-12 Elisa Kit	Boster	Cat# EK0422
Mouse IL-10 Elisa Kit	Boster	Cat# EK0417
Mouse TGF-β Elisa Kit	Boster	Cat# EK0515
