# Supplementary material for: Spleen‐targeted neoantigen DNA vaccine for personalized immunotherapy of hepatocellular carcinoma
Source: EMBO Mol Med. 2023 Aug 8;15(10):e16836. doi: 10.15252/emmm.202216836 (PMC10565630; doi:10.15252/emmm.202216836)
Supplement: Supplementary file 2 — Expanded View Figures PDF [file EMMM-15-e16836-s002.pdf]

Expanded View Figures

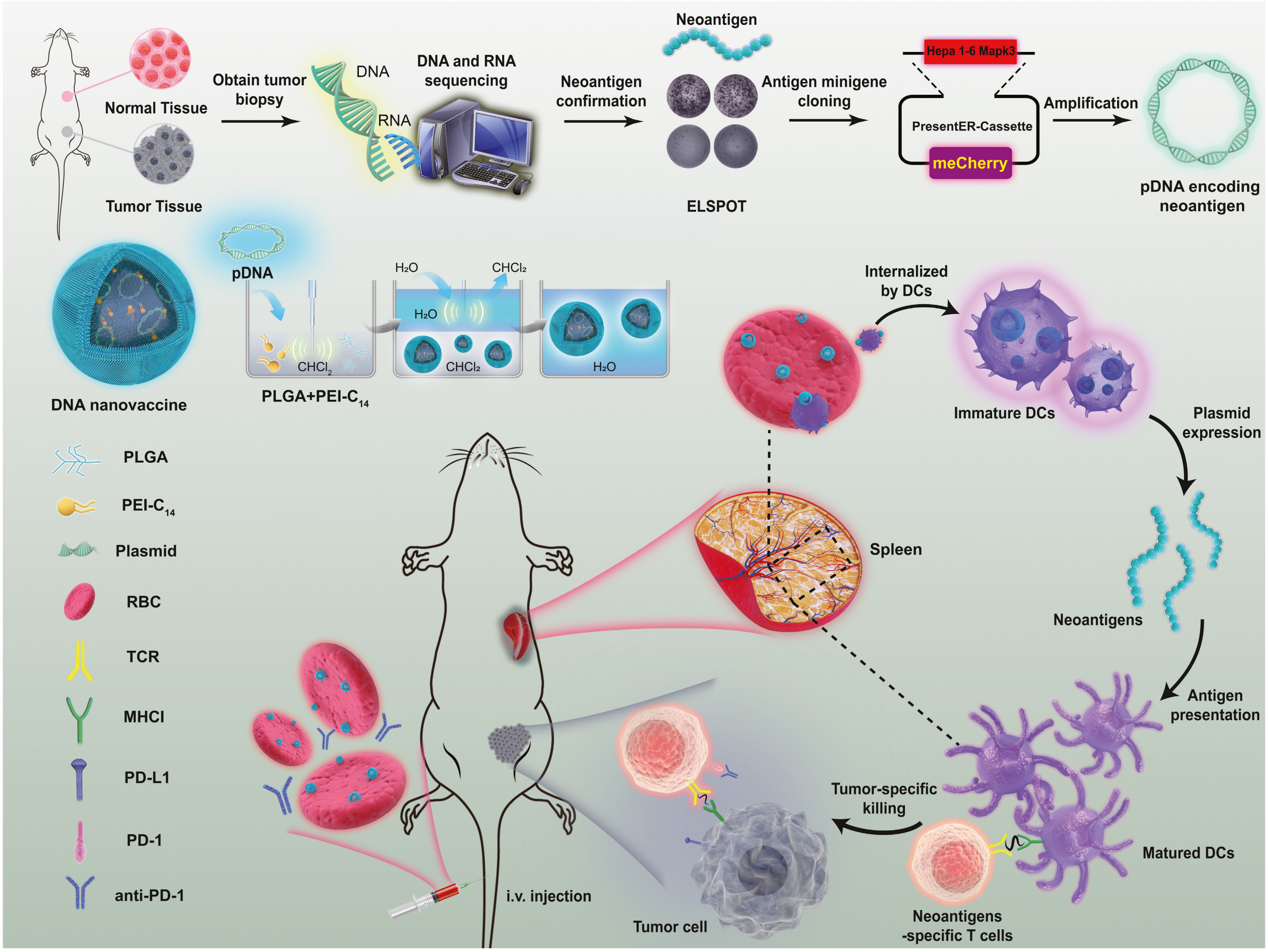

Figure EV1. Schematic illustration of the highly efficient DNA neoantigen vaccine by utilizing a cascade delivery strategy to drive personalized immunotherapy against HCC.

The Hepa 1-6 tumor-specific neoantigens were firstly screened by transcriptome sequencing and whole-exome sequencing, and the pDNA encoding neoantigens were constructed by using "PresentER" cassette. Subsequently, the DNA nanovaccines were synthesized by the double microemulsion method (W/O/W) with PEI_{25000}-C_{14} and PLGA, and then attached on the surface of the preisolated RBCs by the electrostatic adsorption to form a RBC-hitchhiking DNA nanovaccine (RBC-Nanovaccine). After systemic delivery, RBC-Nanovaccines are accumulated into the body's biggest lymphoid organ (spleen) by leveraging the innate "blood filtration" function of RBCs, then dislodging them from the RBCs to antigen-presenting cells (APCs) to promote the neoantigen production and cross-presentation. Finally, antigen-specific immune responses were activated, which prevented tumorigenesis and inhibited tumor growth and recurrence especially in combination with anti-PD-1 antibody, as evidenced in different models of Hepa 1-6 tumor-bearing mice.

© 2023 The Authors

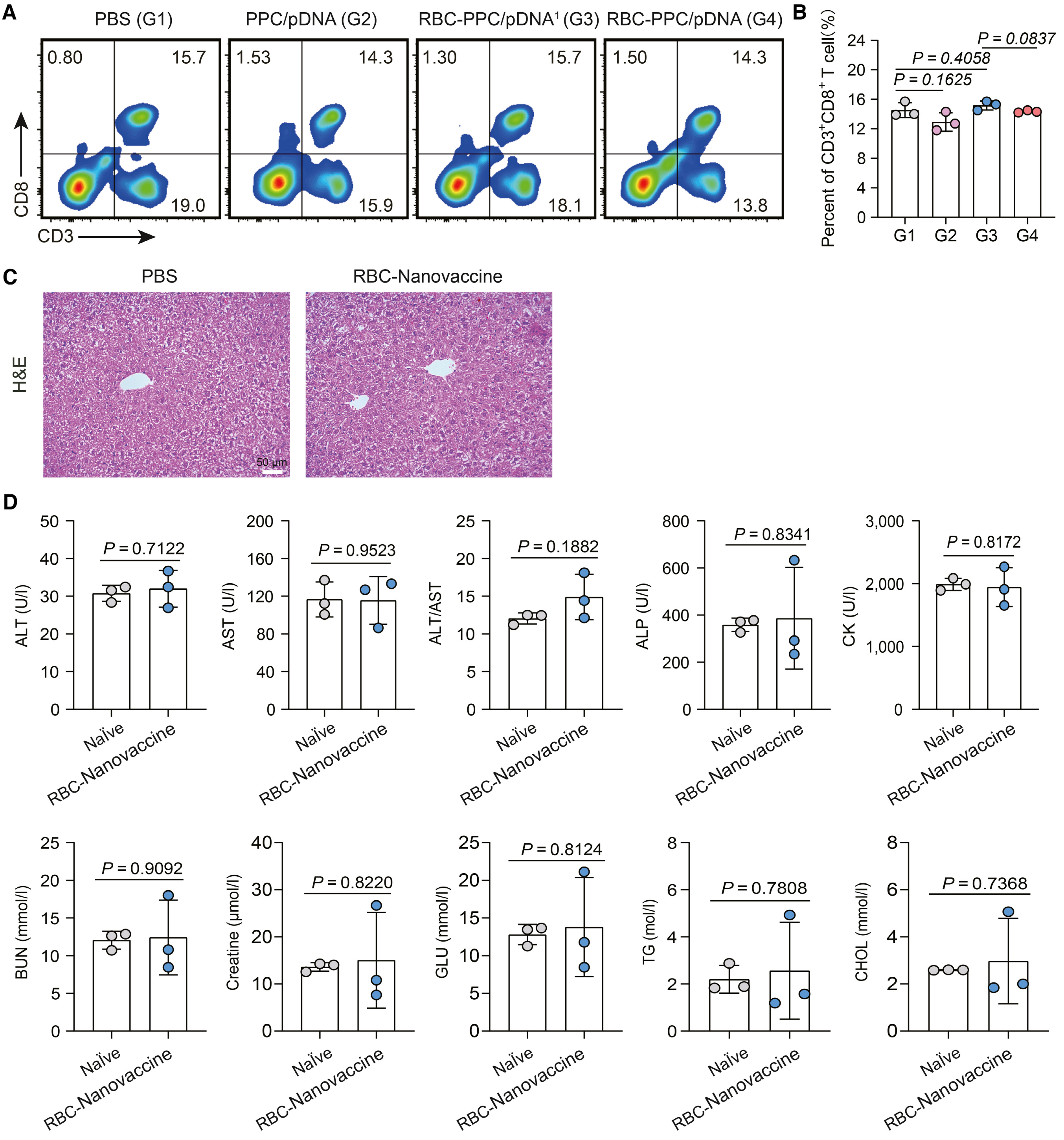

Figure EV2. Immune response in the liver tissue after RBC-Nanovaccine immunization.

A, B The percentage of $CD3^+CD8^+$ T cells in the liver after different treatments analyzed by flow cytometry (n = 3 animals per group). Data in (B) are presented as mean ± SD. Statistical significance was calculated by two-tailed Student's t-test. $P > 0.05$ means no significant difference.

C H&E stained micrographs of the liver tissue collected from PBS and RBC-Nanovaccine treated mice.

D Serum biochemistry data of Naïve group and RBC-Nanovaccine treated group, including alanine aminotransferase (ALT), aspartate aminotransferase (AST), alkaline phosphatase (ALP), ALT/AST, creatine kinase (CK), blood urea nitrogen (BUN), creatine, glucose acid (GLU), serum total cholesterol (TG), and cholesterol (CHOL) (n = 3 animals per group). Data are presented as mean ± SD. Statistical significance was calculated by unpaired two-tailed Student's t-test (B). $P > 0.05$ means no significant difference.

 © 2023 The Authors

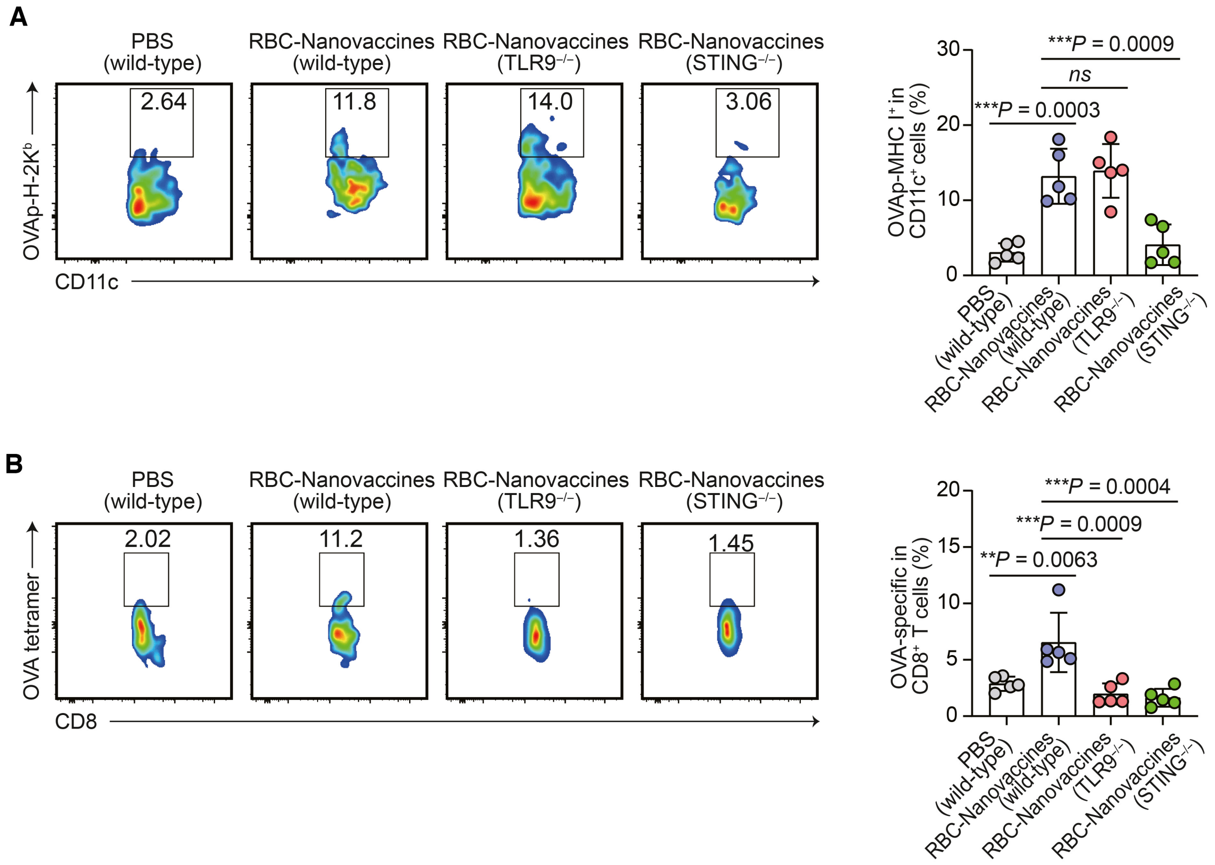

Figure EV3. The specific immune response driving by RBC-Nanovaccines by using OVA as the model antigen.

A Flow cytometry analysis of OVAp-H-2K^b complex-positive DCs in C57BL/6 wild-type, TLR9$^{-/-}$, and STING$^{-/-}$ mice (n = 5 animals per group).

B Flow cytometry analysis of OVAp-tetramer-positive CD8$^+$ T cells in C57BL/6 wild-type, TLR9$^{-/-}$, and STING$^{-/-}$ mice (n = 5 animals per group).

Data information: Data are presented as mean ± SD. Statistical significance was calculated by one-way ANOVA with Tukey's multiple comparison test. **P < 0.01 and ***P < 0.001.

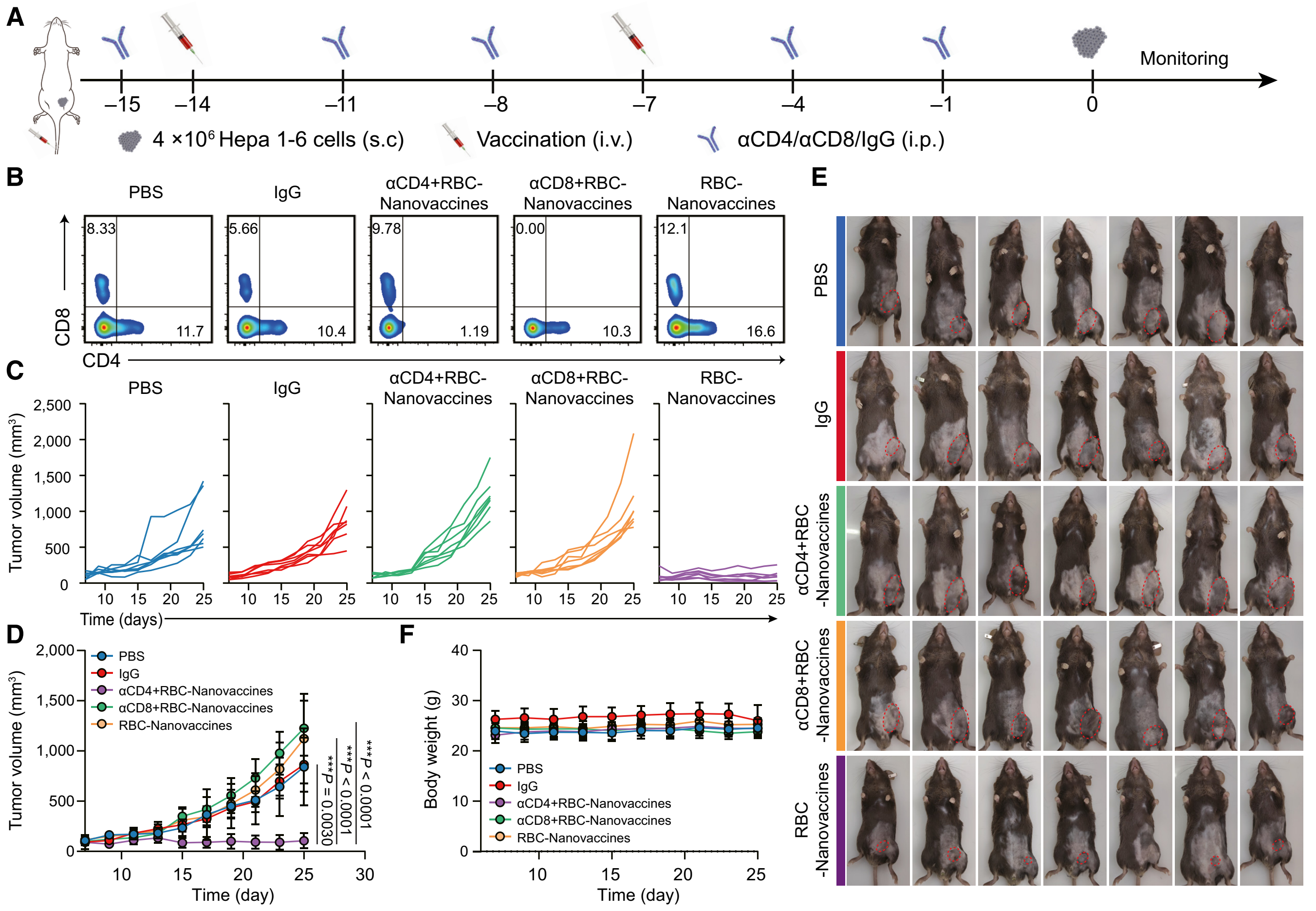

Figure EV4. RBC-Nanovaccines evoke prophylactic protection through CD4$^+$ T and CD8$^+$ T dependent cell responses.

A Schematic illustration of the schedule for the prophylactic protection against Hepa 1-6 tumor.

B The percentages of CD4$^+$ and CD8$^+$ T cells in the peripheral blood after receiving different treatments, which were analyzed by flow cytometry on the 0th day.

C, D Individual growth curves (C) and average tumor growth curves (D) after receiving different treatments (n = 7 animals per group).

E Representative images of Hepa 1-6 tumor-bearing mice in each group at the 25th day. The tumor area was labeled with red dashed circle.

F The body weight change of Hepa 1-6 tumor-bearing mice during the course of various treatments (n = 5 animals per group).

Data information: Data are presented as mean ± SD. Statistical significance was calculated by one-way ANOVA with Tukey's multiple comparison test. ***P < 0.001.

Figure EV5. Therapeutic effects of the RBC-hitchhiking DNA nanovaccine combining with anti-PD-1 antibody in the established Hepa 1-6 HCC tumor model.

A Optical microscopy images of H&E, Ki67 antigen immunohistochemistry staining, and TUNEL staining of tumor slices after receiving different treatments. Scale bar: 50 μm.

B Body weight change of Hepa 1-6 tumor-bearing mice after vaccination as indicated. Data are expressed as mean ± SEM (n = 8 animals per group).

C Histological analysis of H&E staining of mice major organs (heart, liver, spleen, lung, and kidney) collected from Hepa 1-6 tumor bearing mice receiving indicated treatments.

© 2023 The Authors

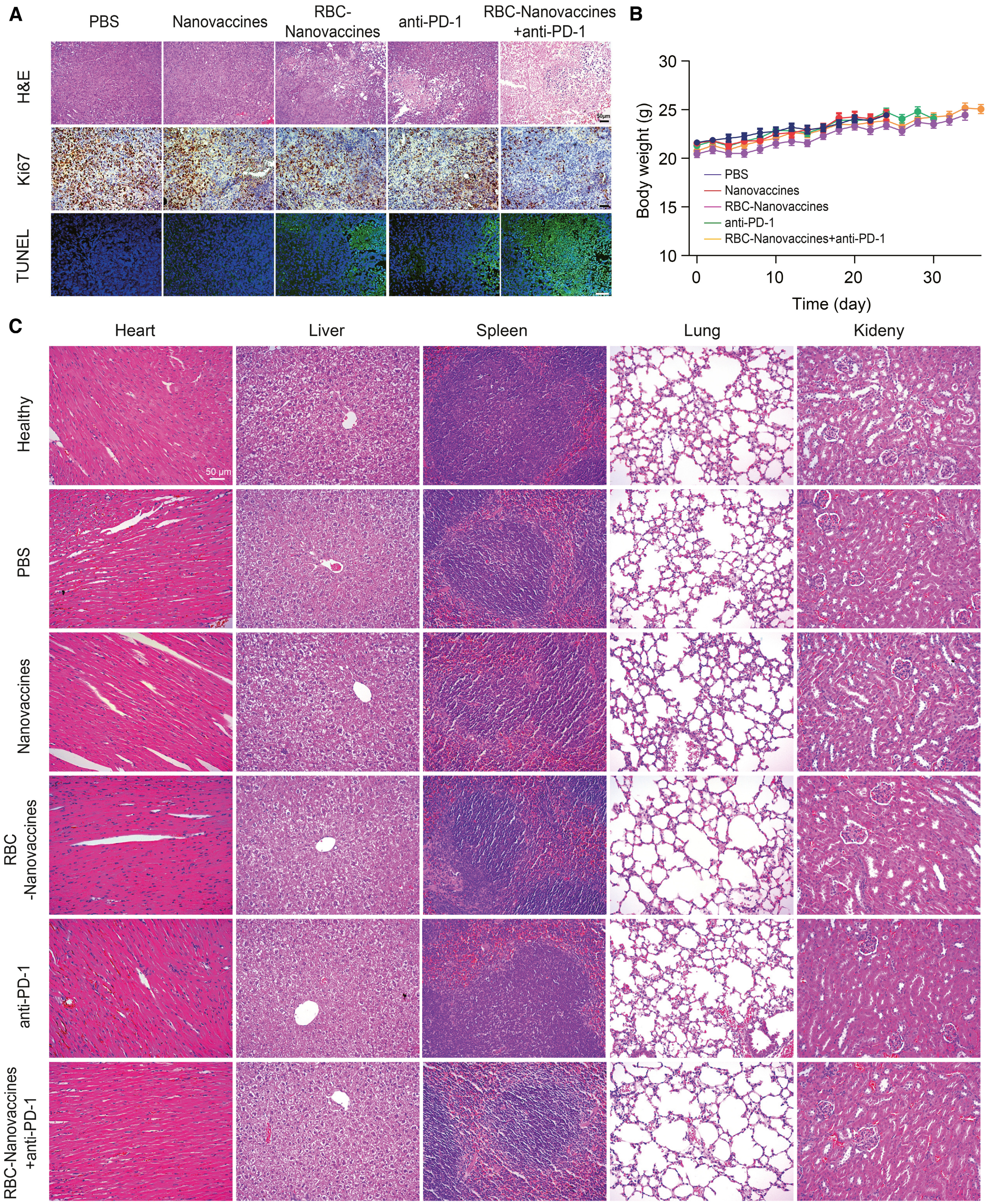
A
PBS
Nanovaccines
RBC-Nanovaccines
anti-PD-1
RBC-Nanovaccines +anti-PD-1
H&E
Ki67
TUNEL
B
Body weight (g)
Time (day)
PBS
Nanovaccines
RBC-Nanovaccines
anti-PD-1
RBC-Nanovaccines+anti-PD-1
C
Heart
Liver
Spleen
Lung
Kideny
Healthy
PBS
Nanovaccines
RBC -Nanovaccines
anti-PD-1
RBC-Nanovaccines +anti-PD-1
50 μm

Figure EV5.
