# Supplementary material for: Spleen‐targeted neoantigen DNA vaccine for personalized immunotherapy of hepatocellular carcinoma
Source: EMBO Mol Med. 2023 Aug 8;15(10):e16836. doi: 10.15252/emmm.202216836 (PMC10565630; doi:10.15252/emmm.202216836)
Supplement: Supplementary file 11 [file EMMM-15-e16836-s006.pdf]

EMBO Molecular Medicine

Spleen targeted neoantigen DNA vaccine for personalized immunotherapy of hepatocellular carcinoma

Ming Wu, Zijin Luo, Zhixiong Cai, Qianqian Mao, Zhenli Li, Hao Li, Cao Zhang, Yuting Zhang, Aoxue Zhong, Liming Wu, and Xiaolong Liu
DOI: 10.15252/emmm.202216836

Corresponding author(s): Xiaolong Liu (xiaoloong.liu@gmail.com) , Liming Wu (wlm@zju.edu.cn)

Review Timeline:

Submission Date:	3rd Sep 22
Editorial Decision:	19th Sep 22
Revision Received:	17th Jun 23
Editorial Decision:	14th Jul 23
Revision Received:	21st Jul 23
Accepted:	25th Jul 23

Editor: Jingyi Hou / Lise Roth

Transaction Report:

(Note: With the exception of the correction of typographical or spelling errors that could be a source of ambiguity, letters and reports are not edited. Depending on transfer agreements, referee reports obtained elsewhere may or may not be included in this compilation. Referee reports are anonymous unless the Referee chooses to sign their reports.)

19th Sep 2022

Dear Dr. Liu,

Thank you again for submitting your work to EMBO Molecular Medicine. We have now heard back from three referees who agreed to evaluate your manuscript. As you will see from the reports below, the referees acknowledge the potential interest of the study. However, they raise a series of concerns, which we would ask you to address in a revision of the manuscript.

Without reiterating all the points raised in the reviews below, some of the more substantial issues are the following:

- Referee #1's concern about whether the vaccine can drive antigen-specific immune response after vaccination.
- Referee #2 also mentioned that the analysis of specific immune response remains preliminary and requested CD8+T cell depletion experiment to better demonstrate their role in tumor growth inhibition.
- Referee #3's points 1 and 2 should be discussed, but addressing them experimentally is not mandatory for publication.

During our pre-decision cross-commenting (in which the referees are given a chance to make additional comments, including on each other's reports), Referees #1 and #2 made additional comments, which I have included below.

Please feel free to contact me in case you would like to discuss in further detail any of the issues raised by the referees. We would welcome the submission of a revised version within six months for further consideration. Please note that EMBO Molecular Medicine strongly supports a single round of revision. As acceptance or rejection of the manuscript will depend on another round of review, your responses should be as complete as possible.

EMBO Molecular Medicine has a "scooping protection" policy, whereby similar findings that are published by others during review or revision are not a criterion for rejection. Should you decide to submit a revised version, I do ask that you get in touch after three months if you have not completed it to update us on the status.

We are aware that many laboratories cannot function at full efficiency during the current COVID-19/SARS-CoV-2 pandemic and have therefore extended our "scooping protection policy" to cover the period required for a full revision to address the experimental issues. Please let me know should you need additional time, and also if you see a paper with related content published elsewhere.

Please read below for important editorial formatting and consult our author's guidelines for proper formatting of your revised article for EMBO Molecular Medicine.

I look forward to receiving your revised manuscript.

Sincerely,
Jingyi

Jingyi Hou
Editor
EMBO Molecular Medicine

*****

When submitting your revised manuscript, please carefully review the instructions that follow below. We perform an initial quality control of all revised manuscripts before re-review; failure to include requested items will delay the evaluation of your revision.

We require:

1) A .docx formatted version of the manuscript text (including legends for main figures, EV figures and tables). Please make sure that the changes are highlighted to be clearly visible.

2) Individual production quality figure files as .eps, .tif, .jpg (one file per figure). For guidance, download the 'Figure Guide PDF': (https://www.embopress.org/page/journal/17574684/authorguide#figureformat).

3) A .docx formatted letter INCLUDING the reviewers' reports and your detailed point-by-point responses to their comments. As part of the EMBO Press transparent editorial process, the point-by-point response is part of the Review Process File (RPF), which will be published alongside your paper.

4) A complete author checklist, which you can download from our author guidelines (https://www.embopress.org/page/journal/17574684/authorguide#submissionofrevisions). Please insert information in the checklist that is also reflected in the manuscript. The completed author checklist will also be part of the RPF.

5) Please note that all corresponding authors are required to supply an ORCID ID for their name upon submission of a revised manuscript.

6) It is mandatory to include a 'Data Availability' section after the Materials and Methods. Before submitting your revision, primary datasets produced in this study need to be deposited in an appropriate public database, and the accession numbers and database listed under 'Data Availability'. Please remember to provide a reviewer password if the datasets are not yet public (see https://www.embopress.org/page/journal/17574684/authorguide#dataavailability).

In case you have no data that requires deposition in a public database, please state so in this section. Note that the Data Availability Section is restricted to new primary data that are part of this study.

7) For data quantification: please specify the name of the statistical test used to generate error bars and P values, the number (n) of independent experiments (specify technical or biological replicates) underlying each data point and the test used to calculate p-values in each figure legend. The figure legends should contain a basic description of n, P and the test applied. Graphs must include a description of the bars and the error bars (s.d., s.e.m.). See also 'Figure Legend' guidelines: https://www.embopress.org/page/journal/17574684/authorguide#figureformat

8) We would also encourage you to include the source data for figure panels that show essential data. Numerical data should be provided as individual .xls or .csv files (including a tab describing the data). For blots or microscopy, uncropped images should be submitted (using a zip archive if multiple images need to be supplied for one panel). Additional information on source data and instruction on how to label the files are available at

.

9) Our journal encourages inclusion of *data citations in the reference list* to directly cite datasets that were re-used and obtained from public databases. Data citations in the article text are distinct from normal bibliographical citations and should directly link to the database records from which the data can be accessed. In the main text, data citations are formatted as follows: "Data ref: Smith et al, 2001" or "Data ref: NCBI Sequence Read Archive PRJNA342805, 2017". In the Reference list, data citations must be labeled with "[DATASET]". A data reference must provide the database name, accession number/identifiers and a resolvable link to the landing page from which the data can be accessed at the end of the reference. Further instructions are available at .

10) We replaced Supplementary Information with Expanded View (EV) Figures and Tables that are collapsible/expandable online. A maximum of 5 EV Figures can be typeset. EV Figures should be cited as 'Figure EV1, Figure EV2" etc... in the text and their respective legends should be included in the main text after the legends of regular figures.

- For the figures that you do NOT wish to display as Expanded View figures, they should be bundled together with their legends in a single PDF file called *Appendix*, which should start with a short Table of Content. Appendix figures should be referred to in the main text as: "Appendix Figure S1, Appendix Figure S2" etc.

- Additional Tables/Datasets should be labeled and referred to as Table EV1, Dataset EV1, etc. Legends have to be provided in a separate tab in case of .xls files. Alternatively, the legend can be supplied as a separate text file (README) and zipped together with the Table/Dataset file.

See detailed instructions here:

.

11) The paper explained: EMBO Molecular Medicine articles are accompanied by a summary of the articles to emphasize the major findings in the paper and their medical implications for the non-specialist reader. Please provide a draft summary of your article highlighting

- the medical issue you are addressing,

- the results obtained and

- their clinical impact.

This may be edited to ensure that readers understand the significance and context of the research. Please refer to any of our published articles for an example.

12) For more information: There is space at the end of each article to list relevant web links for further consultation by our readers. Could you identify some relevant ones and provide such information as well? Some examples are patient associations, relevant databases, OMIM/proteins/genes links, author's websites, etc...

13) Author contributions: the contribution of every author must be detailed in a separate section (before the acknowledgments).

14) A Conflict of Interest statement should be provided in the main text.

15) Every published paper now includes a 'Synopsis' to further enhance discoverability. Synopses are displayed on the journal webpage and are freely accessible to all readers. They include a short stand first (maximum of 300 characters, including space) as well as 2-5 one-sentences bullet points that summarizes the paper. Please write the bullet points to summarize the key NEW findings. They should be designed to be complementary to the abstract - i.e. not repeat the same text. We encourage inclusion of key acronyms and quantitative information (maximum of 30 words / bullet point). Please use the passive voice. Please attach these in a separate file or send them by email, we will incorporate them accordingly.

Please also suggest a striking image or visual abstract to illustrate your article as a PNG file 550 px wide x 300-600 px high.

EMBO Molecular Medicine has a "scooping protection" policy, whereby similar findings that are published by others during review or revision are not a criterion for rejection. Should you decide to submit a revised version, I do ask that you get in touch after three months if you have not completed it, to update us on the status.

***** Reviewer's comments *****

Referee #1 (Comments on Novelty/Model System for Author):

Major concerns:
1. Authors have not shown definitive evidence that nano-packaged DNA can be taken up by APC cells (like DC) in spleen, and present the antigenic signal to T cells.
2. In invitro experiments (Fig1 j&k), authors used YOYO-3 and orange DNA fluorescent stain to trace the DNA inside cell. DNAs are uptake into cells and transported into nuclei. However, in the figure, the red signal exclusively appeared in cytoplasm. Please explain. In Fig1 L&M, readers are not clear whether authors used YOYO-3 labeled DNA for expression. In this case, YOYO-3 orange signal could be mis-judged as mCherry signal after the plasmid DNA is transcribed and translated.

3. Authors used neoantigen derived from patients, for which well-characterized corresponding TCRs are lacking. In this case, the current work showed weak evidence that the Nano-package DNAs are driven antigen specific T cell response. I would suggest authors used model antigens, like OVA, for which both CD4 TCR (OT-II) and CD8 TCR (OT-1) can be used to definitely show that antigen be presented by APCs and that these APCs drive antigen-specific T cell response. Otherwise, the manuscript is making a weak argue.

4. In figure 3, authors try to persuade readers that nano-vector delivered pDNAs facilitated maturation of APCs by measuring CD80 and CD86. Authors also showed the activation state of T cells (cytokine and effector molecules secreted by T cells). This is not a strong argue. DNAs function as TLR agonist, which could effectively promote maturation of DCs and thereby activate T cells. The definite measurement of DC function in this case is to measure the antigen-MHC conjugate presented on DC plasma surface. Parallelly, authors should show the increase in number/percentage and activation state of antigen-specific T cells in peripheral or tumor tissues.

Minor issue:
1. Page 5-6: In our previous study, a Hepa1-6 tumor cell specific neoantigen (MKARNYLQSLPSKTKVA → MKARNYLQFLPSKTKVA) was identified with high affinity to MHC-I H-2Kb (C57BL/6 mice) based on whole exome sequencing and transcriptomic sequencing of murine HCC cell line (Hepa1-6 cells) and C57BL/6 mouse tail tissue by our home-established bioinformatic strategies, and further validated with strong anti-tumor immunogenicity in vivo. Afterward, the sequence of neoantigen peptide was engineered into DNA plasmid (pDNA) by using 'PresentER' cassette comprised of endoplasmic reticulum (ER) signal sequence followed by a short peptide/epitope, which enabled readily display of neoantigen epitope on APC cells according to previously reported methods (Figure 1A) (25).

None of the authors of current manuscript appeared in Reference 25. Please provide the exact reference.
2. In supplementary figure 2B, the 293 cells transfected with pDNA encoding mCherry. However, quite some cells show yellow signal. Where does the yellow signal come from?
3. Page 3. The intracellular red signal from YOYO-3-labeled pDNA well correlated with incubation time, and the international efficacy (in terms of mean MFI detected by flow cytometry) of DNA nanovaccines was much higher than free pDNA. The word "international" is probably a typo of "internalization".

Referee #1 (Remarks for Author):

there are quite some model antigens that could be used in this study to trace the transferring, endocytosis by DCs, and presenting process. Moreover, transgenic T cells specific for MHC-bound antigen will show the antigen-driven activation of these T cells.

Referee #2 (Comments on Novelty/Model System for Author):

The authors present a new formulation of DNA based on polymer lipid nanoparticles adhered to red blood cells. The rationale of this approach is convincing, based on the work of Mitragotri's group. The characterization of the vaccine is well described and its ability to target the spleen without being degraded by its passage through the lung. Different tumor models including orthotopic ones are developed and the results of the vaccine in these models support the strategy selected based on the combination of the vaccine with anti-PD-1.
1) My main criticisms concern the analysis of the specific immune response after vaccination and in particular the specific T lymphocyte response. Indeed, this response is mainly mentioned in figure 3F which remains very preliminary. It would be necessary to show whether this response persists after blocking with an anti-CMH class I or II antibody to define whether it is a predominant CD4+ or CD8+T cell response.
A neoepitope identified by the group (MKARNYLQFLPSKTKVA) seems to have been introduced into the plasmid. It would be necessary to identify the CD4+ and CD8+T lymphocyte responses against this peptide obtained after vaccination. Tetramer assays would be most suitable or at least cytometric assays with detection of intracellular cytokines but after sensitization of PBMCs with the specific neoepitope derived from HEPA 1-6. In Figure 5 I-J, cytometry images with IFNg detection are shown, but it does not appear that the cells were stimulated with tumor antigen.
It would also be important to know whether the vaccine response discriminates the neoepitope from the wild-type peptide.

2) In a general way, the change in immune subpopulations shown by cytometry by the authors (Fig 4 GH, Fig 5 IJ, Fig 6B, Fig 6C) cannot be related to an anti-tumor response, because this specificity is not shown (Fig 4 GH, Fig 5 IJ, Fig 6B, Fig 6C)
In the tumor protection experiments, the authors could deplete CD8+T lymphocytes to identify their role in this tumor growth inhibition

3) Figure 5L made from 3 mice/group is very heterogeneous and should be confirmed with more mice

4) In the discussion, it is mentioned that "Compared with DC and peptide formulations, DNA vaccines can evoke a long-term immune response as means to persistently encode antigenic proteins once successfully being transfected into APCs". Can the authors provide references to support this statement?

5) Statistical analyses are sometimes missing in some figures (1N, 2 HIJ, 5 KL...) and the number of times the experiments were replicated is not mentioned

6) Isn't it contradictory that the authors do not mention any toxicity with the presence of IFNg and TNFa in the blood of mice (Fig 3D). Did the authors measure CRP in these mice and did they not show clinical signs related to cytokine release syndrome

Minor Points
- The reference is missing in the discussion of the GNOS-PV02 clinical trial failure?
- In the introduction it is mentioned that liver cancer patients do not respond to immunotherapy. This sentence should be nuanced following the recent clinical successes also obtained by immunotherapy in this indication (Finn RS N Engl J Med 2020)

Referee #2 (Remarks for Author):

See my comments on item 4

Referee #3 (Comments on Novelty/Model System for Author):

The manuscript described a highly efficient DNA neoantigen vaccine by utilizing a cascade delivery strategy to improve their bioavailability in the spleen to drive personalized immunotherapy against HCC. Generally speaking, the experiments were all well designed and the authors have provided detailed data. However, this study lacks significant innovation. Erythrocyte-driven immune targeting has been reported widely and is an effective strategy to enhance systemic immunity (10.1073/pnas.200288011, 10.1126/sciadv.abi632). Furthermore, RBC-based vaccine (SQZ-AAC-HPV) has been approved

Referee #3 (Remarks for Author):

1. This study uses DNA to encode neoantigens, is there a risk that DNA will be integrated into the genome? I recommend using mRNA to encode neoantigens.
2. It is suggested that DNA-nanovaccine can be further modified with APC targeting modules to improve the specificity of transfection.
3. It is suggested to evaluate the stability of RBC-hitchhiking DNA nanovaccines in blood circulation. Compared with RBC-hitchhiking DNA nanovaccines, erythrocyte membrane encapsulated with DNA nanovaccines may be able to further protect DNA nanovaccines in blood circulation.
4. The author should introduce the principle of red blood cells adsorbing DNA nanovaccines. What are the requirements of this strategy for nanovaccines design?
5. The author confirmed pDNA-NP can promote DC maturation, please give some explanation.
6. It can be seen that RBC-hitchhiking DNA nanovaccines are also enriched in the liver. Will this strategy have obvious side effects on the liver?
7. When doing immune-related experiments, it is recommended that the number of mice in each group should be n=5 or more.

Referees' cross-commenting notes:

Referee #1
The major concerns on ability of nano-packaged vaccine to drive antigen specific T cell response can be addressed. Authors should package model antigens, for example OVA, and using tetramers to measure the increase of OT-1 or OT-2 equivalent T cells. This experiment should be done in paprel on mice of wildtype, CGAS-STINNG pathway deficient, and TLRs (for DNA) deficient background. These experiments can very clearly address reviewers' concers.

Referee #2
I think that the major concern raised by the manuscript on the absence of detailed analysis of antigen-specific T cell response could be addresed after a major revision.
I agree with the note. To assess the quality of the specific T cell response, the author could use tetramer with intracytoplasmic detection of cytokine and/or multicytokine Fluorospot/Elispot sorted CD4+ or CD8+T cells (Launay O et al N Engl J Med 2022). OVA antigen or the neoepitope identified by the authors may be selected if immunogeneic peptides for neoepitope are available.
I think that a depletion of CD8+T cells in the tumor protection experiment will also add insight in the vaccine induced immune responses which mediate this effect.

***** Reviewer's comments *****

Referee #1 (Comments on Novelty/Model System for Author):

Major concerns:

1. *Authors have not shown definitive evidence that nano-packaged DNA can be taken up by APC cells (like DC) in spleen, and present the antigenic signal to T cells.*

Response: Thanks very much for the excellent suggestion. To verify that APC cells in the spleen could uptake the nano-packaged DNA, the DiI labeled RBC-Nanovaccines were intravenously injected in to C57BL/6 mice. After 24 h of injection, the percentage of $DiI^{+}CD11c^{+}$ DCs isolated from the spleen were analyzed by flow cytometry. Meanwhile, according to reviewer 3's suggestion, we have also added Nanovaccines coated with RBC membrane (Nanovaccines@RBCM) rather than adsorbed on RBC as a comparison group. As shown in Appendix Figure S13, mice treated with RBC-Nanovaccines demonstrated significantly higher uptake efficacy in DCs, with a dual positive ratio of 9.83 ± 1.86%, compared to those treated with pure Nanovaccines (4.45 ± 0.84%) or Nanovaccine@RBCM (4.12 ± 1.12%). The corresponding results and explanations have been added in "page 11, line 4-10".

Furthermore, we also used OVA as the model antigen to verify the antigen presentation to T cells, which has been clarified in detailed in the following comments.

2. *In in vitro experiments (Fig1 j&k), authors used YOYO-3 and orange DNA fluorescent stain to trace the DNA inside cell. DNAs are uptake into cells and transported into nuclei. However, in the figure, the red signal exclusively appeared in cytoplasm. Please explain. In Fig1 L&M, readers are not clear whether authors used YOYO-3 labeled DNA for expression. In this case, YOYO-3 orange signal could be mis-judged as mCherry signal after the plasmid DNA is transcribed and translated.*

Response: Thanks very much for the excellent suggestion. To examine the cellular uptake and nuclear transport of the DNA nanovaccines, we extended the incubation time of YOYO-3-labeled DNA nanovaccines with DCs. After 24 hours, we observed a

partial colocalization of blue and red signals (Appendix Figure S7), indicating that a fraction of the DNA nanovaccines successfully delivered the DNA into the nucleus.

Regarding the use of mCherry and YOYO-3, we apologize for the confusion and misunderstanding to readers. In fact, for the cellular uptake experiments, we used DNA without mCherry tag but stained with YOYO-3. For transfection validation, we used mCherry-tagged DNA without YOYO-3 staining. However, neither of these fluorescent dyes was used in the subsequent functional experiments. We have included this information in the caption of Figure 1.

3. *Authors used neoantigen derived from patients, for which well-characterized corresponding TCRs are lacking. In this case, the current work showed weak evidence that the Nano-package DNAs are driven antigen specific T cell response. I would suggest authors used model antigens, like OVA, for which both CD4 TCR (OT-II) and CD8 TCR (OT-1) can be used to definitely show that antigen be presented by APCs and that these APCs drive antigen-specific T cell response. Otherwise, the manuscript is making a weak argue.*

Response: Thanks very much for the excellent suggestion. Based on the reviewer's suggestion, we performed additional experiments using OVA as a model antigen to examine the impact of RBC-Nanovaccines on antigen cross-presentation and the induction of specific T cell responses. In line with the vaccination protocol depicted in Figure 3, we intravenously immunized C57BL/6 wild-type mice with two doses of RBC-Nanovaccines carrying pDNA encoding the MHC I-restricted $OVA_{257-264}$ peptide (SIINFEKL). Furthermore, in response to the following reviewer's suggestion, we included C57BL/6 mice that are genetically deficient for STING ($STING^{-/-}$) or TLR9 ($TLR9^{-/-}$) as the control groups. These additional studies allowed us to assess the contributions of these two proteins (STING and TLR9), which are crucial in the DNA sensing pathway for initiating immune responses.

As shown in Fig EV1, after immunization of RBC-Nanovaccines, the presentation of $OVA_{257–264}$ by MHC I $H\text{-}2K^b$ ($H\text{-}2K^b$-OVA) was clearly detected in splenic $CD11c^+$ DCs of wild type mice and $TLR9^{-/-}$ mice, while there was almost no $H\text{-}2K^b$-OVA

positive signal in the splenic $CD11c^+$ DCs collected from $STING^{-/-}$ mice. Next, we further determined the OVA-specific T cells in splenocytes. Remarkably, immunization with RBC-Nanovaccines resulted in a much higher proportion of antigen-specific T cells in wild-type mice, with the percentage of OVA $tetramer^+$ in $CD3^+CD8^+$ cells reaching 6.55 ± 2.64%. In comparison, the percentage observed in $STING^{-/-}$ mice was 1.64 ± 0.79%, and in $TLR9^{-/-}$ mice was 1.97 ± 0.94%. In $TLR9^{-/-}$ mice, antigenic epitopes were found to be presented on DC cells, but no antigen-specific T cells were detected. This absence of antigen-specific T cells can be attributed to the essential role of TLR9 signaling pathways in T cells, which are required for their effective clonal expansion (*Immunol Res 45: 25-36*). Collectively, these results demonstrated that RBC-Nanovaccines could induce antigen-specific immune response, which is closely associated with the DNA-activated STING and TLR9 pathways.

These explanations have been added in "page 13, line 14 - page 14, line 8".

4. *In figure 3, authors try to persuade readers that nano-vector delivered pDNAs facilitated maturation of APCs by measuring CD80 and CD86. Authors also showed the activation state of T cells (cytokine and effector molecules secreted by T cells). This is not a strong argue. DNAs function as TLR agonist, which could effectively promote maturation of DCs and thereby activate T cells. The definite measurement of DC function in this case is to measure the antigen-MHC conjugate presented on DC plasma surface. Parallelly, authors should show the increase in number/percentage and activation state of antigen-specific T cells in peripheral or tumor tissues.*

Response: Thanks very much for the excellent suggestion. As mentioned above, we employed ovalbumin (OVA) as the model antigen to assess the presentation of OVA antigen-MHC complexes on the surface of DCs in the spleen of immunized mice. To detect antigen-specific T cells, we utilized $H\text{-}2K^b$-OVA (SIINFEKL) tetramers. As control groups, we included mice with STING knockout or TLR9 knockout to investigate the involvement of these pathways in the antigen-specific immune response. The results of this experiment, along with a detailed description, have been

presented in Figure EV1 and are described in “page 13, line 14 - page 14, line 8”.

Minor issue:

1. *Page 5-6: In our previous study, a Hepa1-6 tumor cell specific neoantigen (MKARNYLQSLPSKTKVA → MKARNYLQFLPSKTKVA) was identified with high affinity to MHC-I H-2Kb (C57BL/6 mice) based on whole exome sequencing and transcriptomic sequencing of murine HCC cell line (Hepa1-6 cells) and C57BL/6 mouse tail tissue by our home-established bioinformatic strategies, and further validated with strong anti-tumor immunogenicity in vivo. Afterward, the sequence of neoantigen peptide was engineered into DNA plasmid (pDNA) by using 'PresentER' cassette comprised of endoplasmic reticulum (ER) signal sequence followed by a short peptide/epitope, which enabled readily display of neoantigen epitope on APC cells according to previously reported methods (Figure 1A) (25).*

None of the authors of current manuscript appeared in Reference 25. Please provide the exact reference.

Response: Thanks very much for the excellent suggestion. We have added the correct reference in the revised manuscript (*Journal for immunotherapy of cancer, 2022, 10, e004389*).

2. *In supplementary figure 2B, the 293 cells transfected with pDNA encoding mCherry. However, quite some cells show yellow signal. Where does the yellow signal come from?*

Response: We apologize for the confusion caused by the yellow signal in the images. The yellow signal was unintentional and occurred due to the usage of pseudo-color during image acquisition. To provide a more visually representative illustration of mCherry expression, we have changed the pseudo-color from yellow to red, as shown in Appendix Figure S2.

3. *Page 3. The intracellular red signal from YOYO-3-labeled pDNA well correlated with incubation time, and the international efficacy (in terms of mean MFI detected by*

flow cytometry) of DNA nanovaccines was much higher than free pDNA. The word "international" is probably a typo of "internalization".

Response: We apologize for this mistake. We have carefully corrected such typos in the revised manuscript.

Referee #1 (Remarks for Author):

There are quite some model antigens that could be used in this study to trace the transferring, endocytosis by DCs, and presenting process. Moreover, transgenic T cells specific for MHC-bound antigen will show the antigen-driven activation of these T cells.

Response: Thanks very much for the excellent suggestion. In accordance with the reviewer's guidance, we conducted an investigation into the antigen presentation process of dendritic cells (DCs) using ovalbumin (OVA) as the model antigen. Additionally, we demonstrated that RBC-Nanovaccines can effectively elicit specific T cell responses by evaluating the levels of OVA epitope (SIINFEKL)-specific $CD8^+$ T cells in the spleen of immunized mice. Moreover, we included STING knockout and TLR9 knockout mice as the comparison groups to further elucidate the involvement of DNA sensing pathways. The findings and corresponding data are presented in Figure EV1.

Referee #2 (Comments on Novelty/Model System for Author):

The authors present a new formulation of DNA based on polymer lipid nanoparticles adhered to red blood cells. The rationale of this approach is convincing, based on the work of Mitragotri's group. The characterization of the vaccine is well described and its ability to target the spleen without being degraded by its passage through the lung. Different tumor models including orthotopic ones are developed and the results of the vaccine in these models support the strategy selected based on the combination of the vaccine with anti-PD-1.

Response: We sincerely appreciate the reviewer’s very positive comments and extremely constructive suggestions.

1) My main criticisms concern the analysis of the specific immune response after vaccination and in particular the specific T lymphocyte response. Indeed, this response is mainly mentioned in figure 3F which remains very preliminary.

Response: Thanks very much for the excellent suggestion. Based on the reviewer's suggestion, we performed additional experiments using OVA as a model antigen to examine the impact of RBC-Nanovaccines on antigen cross-presentation and the induction of specific T cell responses. OVA is a widely used model antigen due to its well-characterized epitopes and corresponding T-cell receptors (TCRs), which allow for the determination of antigen presentation and the identification of antigen-specific T cells.

In line with the vaccination protocol depicted in Figure 3, we intravenously immunized C57BL/6 wild-type mice with two doses of RBC-Nanovaccines carrying pDNA encoding the MHC I-restricted $OVA_{257\text{-}264}$ peptide (SIINFEKL). Furthermore, in response to the above reviewer's suggestion, we included C57BL/6 mice that are genetically deficient for STING ($STING^{-/-}$) or TLR9 ($TLR9^{-/-}$) as the control groups. These additional studies allowed us to assess the contributions of these two proteins (STING and TLR9), which are crucial in the DNA sensing pathway for initiating immune responses.

As shown in Fig EV1, after immunization of RBC-Nanovaccines, the presentation of $OVA_{257–264}$ by MHC I $H\text{-}2K^b$ ($H\text{-}2K^b$-OVA) was clearly detected in splenic $CD11c^+$ DCs of wild type mice and $TLR9^{-/-}$ mice, while there was almost no $H\text{-}2K^b$-OVA positive signal in the splenic $CD11c^+$ DCs collected from $STING^{-/-}$ mice. Next, we further determined the OVA-specific T cells in splenocytes. Remarkably, immunization with RBC-Nanovaccines resulted in a much higher proportion of antigen-specific T cells in wild-type mice, with the percentage of OVA $tetramer^+$ in $CD3^+CD8^+$ cells reaching 6.55 ± 2.64%. In comparison, the percentage observed in $STING^{-/-}$ mice was 1.64 ± 0.79%, and in $TLR9^{-/-}$ mice was 1.97 ± 0.94%. In $TLR9^{-/-}$ mice, antigenic epitopes were found to be presented on DC cells, but no antigen-specific T cells were detected. This absence of antigen-specific T cells can be

attributed to the essential role of TLR9 signaling pathways in T cells, which are required for their effective clonal expansion (*Immunol Res 45: 25-36*). Collectively, these results demonstrated that RBC-Nanovaccines could induce antigen-specific immune response, which is closely associated with the DNA-activated STING and TLR9 pathways.

These explanations have been added in “page 13, line 14 - page 14, line 8”.

It would be necessary to show whether this response persists after blocking with an anti-CMH class I or II antibody to define whether it is a predominant CD4+ or CD8+T cell response.

Response: Thanks very much for the excellent suggestion. To further assess the role of T cell populations in antitumor immune response, we conducted additional experiments to investigate the effects of RBC-Nanovaccines on tumor growth in mice depleted of $CD8^+$ or $CD4^+$ T cells. This was achieved by intraperitoneal injection of the corresponding neutralizing antibodies. The experimental timeline is illustrated in Fig EV2A. Confirmation of $CD8^+$ and $CD4^+$ T cell depletion was performed by flow cytometric analysis of peripheral blood mononuclear cells (PBMCs) (Fig EV2B). Continuous depletion studies (Fig EV2C to E) revealed that the antitumor effects of RBC-Nanovaccines were largely abolished upon depletion of $CD8^+$ T cells. Surprisingly, the protective effect was also lost after depletion of $CD4^+$ T cells, despite selection of neoantigens was based on their affinity to MHC I H-2K^b. These findings collectively demonstrate that the antitumor response induced by RBC-Nanovaccines is dependent on $CD8^+$ T cells through $CD4^+$ T cell-dependent mechanisms. It has been well reported that $CD4^+$ T cells played a synergistic role with $CD8^+$ T cells by recruiting them to the tumor site and enhancing their proliferation and effector functions (*Cancer Res., 1999, 59, 1071–1079; Cancer Res., 2010, 70, 8368–8377*). These explanations have been added in “page 15, line 18 - page 16, line 3”.

A neoepitope identified by the group (MKARNYLQFLPSKTKVA) seems to have been

introduced into the plasmid. It would be necessary to identify the CD4+ and CD8+T lymphocyte responses against this peptide obtained after vaccination.

Response: Thanks very much for the excellent suggestion. Regarding the neoepitope (MKARNYLQFLPSKTKVA) identified by our research group, we previously investigated the $CD4^{+}$ and $CD8^{+}$ T lymphocyte responses to this peptide in a recently published study (*J. Immunother. Cancer, 2022, 10, e004389*). In this study, we sorted $CD4^{+}$ and $CD8^{+}$ T cells from the spleen of immunized mice and stimulated them with autologous dendritic cells (DCs) pulsed with different neoantigen peptides. We assessed the immune response using the IFN-γ ELISPOT assay. Additionally, we compared the immunogenicity of neoantigen peptides with their corresponding wild-type peptides. The corresponding results extracted from that article are depicted in the figure below. Among these peptides, we selected Mapk3_S284F to incorporate into our plasmid. As shown in Figure Q1D, Mapk3_S284F could elicit significant immune responses in immunized mice compared to the corresponding wild-type peptide. Figure Q1E demonstrated that Mapk3_S284F was predominantly presented to $CD8^{+}$ T cells.

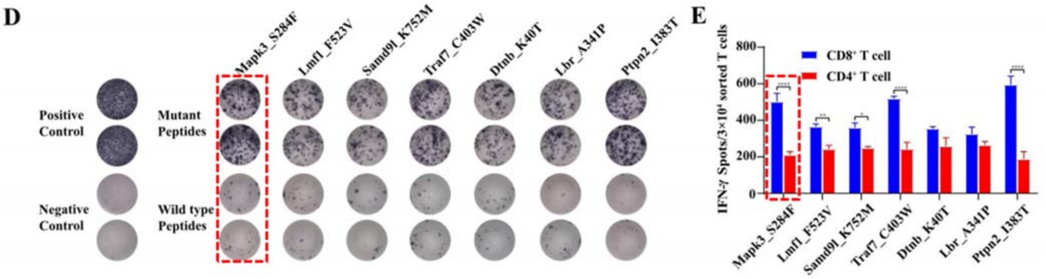

Figure Q1. (D) Cross-reactivity analysis between seven identified neoantigen peptides and corresponding wild-type peptides. (E) Potential neoantigen immunogenicity analysis of seven identified neoantigen peptides in sorted splenic $CD4^{+}$ and $CD8^{+}$ T cells. Results are shown as mean±SD. *p<0.05; **p<0.01; ***p<0.001; ****p<0.0001. Mapk3_S284F was highlighted by red dashed box.

Tetramer assays would be most suitable or at least cytometric assays with detection of intracellular cytokines but after sensitization of PBMCs with the specific neoepitope derived from HEPA 1-6.

Response: Thanks very much for the excellent suggestion. To assess the antigen-

specific immune response following vaccination, we employed Ovalbumin (OVA) as the model antigen and used tetramer assays to confirming antigen-specific T cells, as mentioned above.

In Figure 5 I-J, cytometry images with IFNg detection are shown, but it does not appear that the cells were stimulated with tumor antigen.

Response: Thanks very much for the excellent suggestion. Regarding Figure 5I-J, when we were analyzing the secretion of IFN-γ in tumor-infiltrating T cells, we did not stimulate the T cells with additional neoantigen peptides as we did in spleen samples as shown in Figure 3E, since the tumor tissue itself already contained a large number of neoantigens.

It would also be important to know whether the vaccine response discriminates the neoepitope from the wild-type peptide.

Response: Thanks very much for the excellent suggestion. As mentioned above, we validated the immunogenicity of the novel antigen through IFN-γ ELISPOT assay and used a wild-type peptide as a reference in our pervious work (*J. Immunother. Cancer, 2022, 10, e004389*). As shown in Figure Q1D, Mapk3_S284F could elicit significant immune responses in immunized mice compared to the wild-type peptide.

2) *In a general way, the change in immune subpopulations shown by cytometry by the authors (Fig 4 GH, Fig 5 IJ, Fig 6B, Fig 6C) cannot be related to an anti-tumor response, because this specificity is not shown (Fig 4 GH, Fig 5 IJ, Fig 6B, Fig 6C).*

Response: Thanks very much for the excellent suggestion. To assess the antigen-specific immune response following vaccination, we employed Ovalbumin (OVA) as the model antigen to examine the impact of RBC-Nanovaccines on antigen cross-presentation and the induction of specific T cell responses, as mentioned above.

In the tumor protection experiments, the authors could deplete CD8+T lymphocytes to identify their role in this tumor growth inhibition.

Response: Thanks very much for the excellent suggestion. To further assess the role of T cell populations in preventing tumorigenesis, we conducted additional experiments to investigate the effects of RBC-Nanovaccines on tumor growth in mice depleted of $CD8^+$ or $CD4^+$ T cells. This was achieved by intraperitoneal injection of the corresponding neutralizing antibodies. The experimental timeline is illustrated in Fig EV2A. Confirmation of $CD8^+$ and $CD4^+$ T cell depletion was performed by flow cytometric analysis of peripheral blood mononuclear cells (PBMCs) (Fig EV2B). Continuous depletion studies (Fig EV2C to E) revealed that the antitumor effects of RBC-Nanovaccines were largely abolished upon depletion of $CD8^+$ T cells. Surprisingly, the protective effect was also lost after depletion of $CD4^+$ T cells, despite selection of neoantigens was based on their affinity to MHC I H-$2K^b$. These findings collectively demonstrate that the antitumor response induced by RBC-Nanovaccines is dependent on $CD8^+$ T cells through $CD4^+$ T cell-dependent mechanisms. It has been well reported that $CD4^+$ T cells played a synergistic role with $CD8^+$ T cells by recruiting them to the tumor site and enhancing their proliferation and effector functions (*Cancer Res., 1999, 59, 1071–1079; Cancer Res., 2010, 70, 8368–8377*). These explanations have been added in "page 15, line 18 - page 16, line 3".

3) *Figure 5L made from 3 mice/group is very heterogeneous and should be confirmed with more mice.*

Response: Thanks very much for the excellent suggestion. In response to the reviewer's recommendation, we have increased the sample size in each group from three to five mice. However, the transcriptome sequencing results were affected by tumor heterogeneity, leading to suboptimal consistency. Despite this, we consistently observed an upregulation of pathways associated with chemokines and cytokine receptors in the treatment group across both independent experiments. To address this issue in future experiments, we will consider preselecting immune cells from tumor tissue prior to sequencing as a potential approach to reduce intra-group variability.

4) In the discussion, it is mentioned that "Compared with DC and peptide formulations, DNA vaccines can evoke a long-term immune response as means to persistently encode antigenic proteins once successfully being transfected into APCs". Can the authors provide references to support this statement?

Response: Thanks very much for the excellent suggestion. Following the reviewer's suggestion, the relevant references (*ACS Nano,* 2020, 14, 11623-11636; *Nat. Rev. Cancer, 2008,* 8, 108–120) have been added in the revised manuscript.

5) *Statistical analyses are sometimes missing in some figures (1N, 2 HIJ, 5 KL...) and the number of times the experiments were replicated is not mentioned.*

Response: Thanks very much for the excellent suggestion. We have made the corresponding modifications in the revised manuscript.

Regarding Figure 1N, the statistical differences between different groups were not easily discernible from the heat map. Therefore, to provide a clearer representation, we have included a bar graph in the Appendix Figure S8B along with the corresponding statistical analysis results.

Regarding Figure 2 HIJ, in our previous experiments, only a single sample was analyzed for each group, and hence, no statistical analysis was performed. To strengthen the reliability of our findings, we conducted replicates of these experiments and expanded the sample size to three or more per group. We have incorporated the statistical analysis results directly into the figure. Furthermore, the biological replicate data for each group can be found in Appendix Figure S12 and Figure S14.

Regarding Figure 5K, the statistical differences between different groups were not easily discernible from the heat map presented in Figure 1O. However, in our original version of manuscript, the results of the statistical analysis, presented in the form of bar graphs, were included in Appendix Figure S30.

Regarding Figure 5L, the heat map did not clearly indicate the statistical differences between different groups. However, as mentioned in the "RNA sequencing and bioinformatic analysis" section provided in the Supporting Information of our

original manuscript version, we conducted differential expression analysis between different groups using the limma package. Additionally, KEGG pathway enrichment analysis was performed using the clusterProfiler package with the criteria of differentially expressed genes (|$\log_2$ fold change| > 1, p-value < 0.05).

The information regarding the number of experiments or biological replicates has been included in the figure captions.

6) *Isn't it contradictory that the authors do not mention any toxicity with the presence of IFNg and TNFa in the blood of mice (Fig 3D). Did the authors measure CRP in these mice and did they not show clinical signs related to cytokine release syndrome.*

Response: Thanks very much for the excellent suggestion. Based on Figure 3D, it is evident that despite a slight increase in the levels of IFN-γ and TNF-α in the peripheral blood of immunized mice, both of these indicators remained within the safe range according to the previous literature (*Science Translational Medicine, 2021, 13, eabc2816; Journal of Controlled Release, 2022, 343, 1–12*). Additionally, in response to the reviewer's recommendation, we assessed the concentration of CRP in the peripheral blood. The results, depicted in Figure 3D, demonstrate no significant differences among the groups. Meanwhile, no abnormal phenomena were observed in the test animals. We have added more words in "page 12, line 16-21" to explain this issue.

Minor Points

- *The reference is missing in the discussion of the GNOS-PV02 clinical trial failure?*

Response: Thanks very much. According to the reviewer's suggestion, we have added relevant references. Considering the latest progress in this clinical trial during the process of revising the manuscript, we have made some changes to the original wording. "Unfortunately, the immunogenicity and therapeutic outcome of this DNA vaccine remain in need of improvement, as evidenced by an objective response rate of only 25%, even when used in combination with PD-1 inhibitors and IL-12, as observed in the latest single-arm phase I/II clinical trial (*J. Immunotherapy Cancer,*

2021;9. DOI: 10.1136/jitc-2021-SITC2021.453; *J. Immunotherapy Cancer,* 2022;10, DOI: 10.1136/jitc-2022-SITC2022.0693)"

- In the introduction it is mentioned that liver cancer patients do not respond to immunotherapy. This sentence should be nuanced following the recent clinical successes also obtained by immunotherapy in this indication (Finn RS N Engl J Med 2020).

Response: Thanks very much for the reviewer's suggestion. Indeed, we are aware of the remarkable achievements of atezolizumab in combination with bevacizumab in advanced unresectable liver cancer, which has now become the first-line treatment strategy for advanced liver cancer. However, this paper primarily focuses on the application of tumor neoantigen vaccines as a novel therapy in liver cancer treatment. Hence, we only emphasize the advancements in therapeutic tumor vaccine area and do not deeply discuss other immunotherapy approaches in the Introduction section.

Referee #3 (Comments on Novelty/Model System for Author):

The manuscript described a highly efficient DNA neoantigen vaccine by utilizing a cascade delivery strategy to improve their bioavailability in the spleen to drive personalized immunotherapy against HCC. Generally speaking, the experiments were all well designed and the authors have provided detailed data. However, this study lacks significant innovation. Erythrocyte-driven immune targeting has been reported widely and is an effective strategy to enhance systemic immunity (10.1073/pnas.200288011, 10.1126/sciadv.abi632). Furthermore, RBC-based vaccine (SQZ-AAC-HPV) has been approved.

Response: We sincerely thank to the reviewer for his/her careful reading and making the useful comments. Previous studies have reported various strategies based on RBC, such as RBC-hitchhiking, RBC-encapsulating, or RBC membrane-coating, to enhance the enrichment efficiency of therapeutic molecules in the spleen, thereby improving the effectiveness of tumor immunotherapy. As highlighted in the Introduction and Discussion, our research has been inspired by these findings and aims to harness red

blood cells for the delivery of tumor neoantigens. Taking into account the specific physicochemical properties of neoantigen nucleic acid vaccines and the distinct immune response mechanisms within the body, we investigate the resulting liver cancer-specific preventive and therapeutic effects, as well as the involved underlying immune response mechanisms. To the best of our knowledge, our study represents the first exploration of efficient red blood cell-driven delivery of liver cancer neoantigens for personalized immunotherapy of hepatocellular carcinoma.

Referee #3 (Remarks for Author):

1. *This study uses DNA to encode neoantigens, is there a risk that DNA will be integrated into the genome? I recommend using mRNA to encode neoantigens.*

Response: Thanks very much. For this concern, we performed additional whole-genome sequencing (WGS) to detect potential integration of plasmid in host genome. After sequencing, HIVID2 was deployed to detect integration sites in sequencing data (*Bioinformatics, 2021, 37, 1821–1827*). To avoid interference resulted from sequencing data size of control and test group, 1×10^8 reads was extracted from each sample and subjected to HIVID2 using human reference genome and plasmid sequence. While we observed sequencing reads mapping to both human genome and plasmid sequence, no integration was detected in both samples, confirming that the risk for DNA integration is minimal. The sequencing datasets produced in this study have been deposited at Genome Sequencing Achieve database (GSA, https://hgdc.cncb.ac.cn/gsa) under the accession number of CRA006378.

Regarding the second suggestion, we would like to clarify that the use of mRNA neoantigen vaccines falls outside the scope of this study. Implementing this approach would require a complete redesign and a separate investigation. We appreciate the understanding of the reviewer, and we will take into consideration the use of mRNA vaccines in our future research endeavors.

2. It is suggested that DNA-nanovaccine can be further modified with APC targeting modules to improve the specificity of transfection.

Response: Thanks very much. Modification of carriers with specific targeting modules of specific cells or organs are the most common strategies for active targeting, but facing several challenges as well, such as targeting molecular selection\coupling efficiency\coupling stability......, it is completely another discipline; many FDA approved carrier systems have also applied the passive targeting strategy without targeting molecular modification; furthermore, if we were to incorporate APC targeting molecules into our DNA nanovaccine as suggested by the reviewer, it would necessitate a complete redesign and restart of the entire project. It would essentially become an entirely different endeavor.

3. It is suggested to evaluate the stability of RBC-hitchhiking DNA nanovaccines in blood circulation. Compared with RBC-hitchhiking DNA nanovaccines, erythrocyte membrane encapsulated with DNA nanovaccines may be able to further protect DNA nanovaccines in blood circulation.

Response: Thanks very much for this extremely valuable comment. Following the reviewer's suggestion, we investigated the stability of nanovaccine adsorption on red blood cell surfaces under shear forces present in normal circulation conditions (1 Pa) and within pulmonary capillaries (6Pa). The results indicated that when the loading ratio was above 100:1, the majority of nanovaccines remained stably adhered without detachment (Appendix Figure S11).

Based on the reviewer's suggestion, we also extracted red blood cell membranes and subjected them to ultrasound treatment along with the nanovaccines, aiming to incorporate the cell membrane onto the surface of the nanovaccines. However, due to the strong positive charge of the nanovaccine itself, we observed the formation of aggregates when mixed with negatively charged red blood cell membranes. These aggregates were difficult for DC cells to uptake (Appendix Figure S13); and upon intravenous administration in mice, they exhibited significant deposition in the lungs, which would significantly cause toxicity such as pulmonary embolism and limit the accumulation in the spleen as well (Appendix Figure S12).

These explanations have been added in "page 10, line 26 - page 11, line 10".

4. The author should introduce the principle of red blood cells adsorbing DNA nanovaccines. What are the requirements of this strategy for nanovaccines design?

Response: Thanks very much for the helpful suggestion. As reported by Mitragotri et al. (*PNAS, 2020, 117, 17727-17736*), the hitchhiking of nanoparticles involves two physical steps. First, nanoparticles adsorb onto the surface of erythrocytes, initiating contact. Second, the erythrocyte membrane spreads around the nanoparticles, enhancing the strength of adhesion. They provided evidence using PLGA nanoparticles as an example to demonstrate that the binding between nanoparticles and red blood cells can be facilitated through non-covalent forces such as electrostatic interactions, hydrophobic interactions, and hydrogen bonding (*Nature Biomedical Engineering, 2021, 5, 441-454*). Our nanovaccine primarily utilizes PLGA as its carrier, thereby allowing for adsorption onto the surface of red blood cells through these three types of interactions. Notably, the nanovaccine formulation also incorporates a PEI-C_{14} cationic component, which imparts a positive charge to the PLGA surface, further enhancing the binding to red blood cells via electrostatic interactions. These explanations have been added in "page 21, line 1-13".

5. The author confirmed pDNA-NP can promote DC maturation, please give some explanation.

Response: Thanks very much. There are three reasons for the enhancement of DC maturation by DNA. Firstly, as previously described, PEI_{25000}-C_{14} can activate the TLR4 pathway. Secondly, the DNA vaccine itself can stimulate DC maturation through both TLR9 and STING pathways. Lastly, the nanovaccine improves the efficiency of DC uptake of DNA, thus further enhancing the immunological recognition effects of DNA. We have added relevant explanations in "page 8, line 21-24".

6. It can be seen that RBC-hitchhiking DNA nanovaccines are also enriched in the liver. Will this strategy have obvious side effects on the liver?

Response: Thanks very much. Indeed, we have observed a notable accumulation of

RBC-Nanovaccines in the liver. To assess this influence, we conducted a detailed assessment of the immune response within the liver tissue. Our findings revealed that immunization with RBC-Nanovaccines had a minimal impact on the frequency of $CD3^{+}CD8^{+}$ T cells in the liver (Appendix Figure S19). This can be attributed to the tolerogenic characteristics of local hepatic APCs, including myeloid and plasmacytoid dendritic cells, liver sinusoidal endothelial cells, Kupffer cells, and hepatocytes (*Nature Reviews Immunology, 2010, 10, 753–766*), which limit the effectiveness of DNA nanovaccines. Furthermore, histological analysis of liver tissue and serum chemical analysis of ALT, AST, and ALP (Appendix Figure S20) indicated that RBC-Nanovaccines did not cause significant side effects on the liver.

7. When doing immune-related experiments, it is recommended that the number of mice in each group should be n=5 or more.

Response: We appreciate the valuable suggestion to refine the manuscript. We have increased the number of mice per group in immune-related experiments to five mice per group.

Referees' cross-commenting notes:

Referee #1

The major concerns on ability of nano-packaged vaccine to drive antigen specific T cell response can be addressed. Authors should package model antigens, for example OVA, and using tetramers to measure the increase of OT-1 or OT-2 equivalent T cells. This experiment should be done in paprel on mice of wildtype, CGAS-STINNG pathway deficient, and TLRs (for DNA) deficient background. These experiments can very clearly address reviewers' concers.

Response: Thanks very much for the valuable suggestion. Based on the reviewer's suggestion, we performed additional experiments using OVA as the model antigen to examine the impact of RBC-Nanovaccines on antigen cross-presentation and the induction of specific T cell responses. In line with the vaccination protocol depicted in Figure 3, we intravenously immunized C57BL/6 wild-type mice with two doses of

RBC-Nanovaccines carrying pDNA encoding the MHC I-restricted $OVA_{257-264}$ peptide (SIINFEKL). Furthermore, we included C57BL/6 mice that are genetically deficient for STING ($STING^{-/-}$) or TLR9 ($TLR9^{-/-}$) as the control groups. These additional studies allowed us to assess the contributions of these two proteins (STING and TLR9), which are crucial in the DNA sensing pathway for initiating immune responses.

As shown in Figure EV1, after immunization of RBC-Nanovaccines, the presentation of $OVA_{257–264}$ by MHC I H-$2K^b$ (H-$2K^b$-OVA) was clearly detected in splenic $CD11c^+$ DCs of wild type mice, while there was almost no H-2Kb-OVA positive signal in splenic $CD11c^+$ DCs collected from $STING^{-/-}$ or $TLR9^{-/-}$ mice. Next, we further determined OVA-specific T cells in splenocytes. Remarkably, immunization with RBC-Nanovaccines resulted in a much higher proportion of antigen-specific T cells (OVA $tetramer^+$ in $CD3^+CD8^+$ cells) in wild-type mice compared to both $STING^{-/-}$ or $TLR9^{-/-}$ mice. These results demonstrated that RBC-Nanovaccines could induce antigen-specific immune response, which is also closely associated with the DNA-activated STING and TLR9 pathways.

These explanations have been added in "page 13, line 14 - page 14, line 8".

Referee #2

I think that the major concern raised by the manuscript on the absence of detailed analysis of antigen-specific T cell response could be addresed after a major revision.

I agree with the note. To assess the quality of the specific T cell response, the author could use tetramer with intracytoplasmic detection of cytokine and/or multicytokine Fluorospot/Elispot sorted CD4+ or CD8+T cells (Launay O et al N Engl J Med 2022).

OVA antigen or the neoepitope identified by the authors may be selected if immunogeneic peptides for neoepitope are available. I think that a depletion of CD8+T cells in the tumor protection experiment will also add insight in the vaccine induced immune responses which mediate this effect.

Response: Thanks very much for the extremely valuable suggestion. As mentioned

above, we employed ovalbumin (OVA) as the model antigen to assess the presentation of OVA antigen-MHC complexes on the surface of DCs in the spleens of immunized mice. To detect antigen-specific T cells, we utilized H-2K^b/OVA (SIINFEKL) tetramers. As control groups, we included mice with STING knockout or TLR9 knockout to investigate the involvement of these pathways in the antigen-specific immune response. The results of this experiment, along with a detailed description, have been presented in Figure EV1 and are described in "page 13, line 14 - page 14, line 8".

To further assess the role of T cell populations in preventing tumorigenesis, we conducted additional experiments to investigate the effects of RBC-Nanovaccines on tumor growth in mice depleted of CD8$^+$ or CD4$^+$ T cells. This was achieved by intraperitoneal injection of the corresponding neutralizing antibodies (Figure EV2). The experimental timeline is illustrated in Figure EV2A. Confirmation of CD8$^+$ and CD4$^+$ T cell depletion was performed by flow cytometric analysis of peripheral blood mononuclear cells (PBMCs) (Figure EV2B). Continuous depletion studies (Figure EV2C to E) revealed that the antitumor effects of RBC-Nanovaccines were largely abolished upon depletion of CD8$^+$ T cells. Surprisingly, the protective effect was also lost after depletion of CD4$^+$ T cells, despite selecting neoantigens based on their affinity to MHC I H-2K^b. These findings collectively demonstrate that the antitumor response induced by RBC-Nanovaccines is dependent on CD8$^+$ T cells through CD4$^+$ T cell-dependent mechanisms. It has been reported that CD4$^+$ T cells played a synergistic role with CD8$^+$ T cells by recruiting them to the tumor site and enhancing their proliferation and effector functions (*Cancer Res., 1999, 59, 1071–1079; Cancer Res., 2010, 70, 8368–8377*).

These explanations have been added in "page 15, line 18 - page 16, line 3".

14th Jul 2023

Dear Dr. Liu,

Thank you for the submission of your revised manuscript to EMBO Molecular Medicine. We have now received the reports from the 3 referees who re-reviewed your manuscript. As you will see, they are supportive of publication pending minor revisions, and we will therefore be able to accept your manuscript once the following points will be addressed:

1/ Referees' comments: please address the remaining minor concerns/questions from referee #1 in writing.

2/ Main manuscript text:
- Please address the queries from our data editors in the related "Data edited MS file", remove the red text and only keep in track changes any new modification.
- As mentioned also by referee #1, the text needs to be carefully proof-read for grammar and typos.
- Materials and methods:
o Cell lines: please indicate whether the cells were tested for mycoplasma contamination and authenticated. Please adjust the checklist accordingly.
o Antibodies: please provide dilutions/concentrations used
o Data Availability: please note that the data must be public before acceptance of the manuscript. Please also check that the link you provided is correct. Kindly remove the sentence: "Expanded View for this article is available online."
- Acknowledgements: The funding information should match the information provided in the submission system: Funding 2022-S-rc1 is currently missing from the submission system.
- Please remove "correspondence information" from p. 34
- The "Captions" heading should be changed to "Figure Legends"

3/ Figures and Appendix:
- We note that you currently have 2 EV figures, and 32 Appendix figures. Please note that you have the possibility to make some of the Appendix figures EV figures, expendable online (up to 5 EV figures).
- Appendix figure S24 is currently not referenced in the manuscript text, please adjust accordingly.
- If images are reused in different figures, it must be stated in the figure legends. We noted possible re-use of images in figure 6H and Appendix Figure S23
- Please check the legend of Appendix Figure S25: it references Figure 5F, should it be 6F?
- We would also encourage you to include the source data for figure panels that show essential data. Numerical data should be provided as individual .xls or .csv files (including a tab describing the data). For blots or microscopy, uncropped images should be submitted (using a zip archive if multiple images need to be supplied for one panel). Additional information on source data and instruction on how to label the files are available at
.

4/ Thank you for providing a synopsis text and image. I slightly edited the text to fit with our style and format, please let me know if you agree with the following or amend as you see fit:

To address the limited efficacy of current personalized cancer vaccines and improve hepatocellular carcinoma (HCC) immunotherapy, a red blood cell (RBC) hitchhiking strategy was developed for targeted delivery of a neoantigen DNA vaccine to the spleen.
- Polymer-lipid nanoparticles (NPs) were designed to encapsulate neoantigen-carrying plasmid DNA (pDNA). These nanovaccines were then attached to pre-isolated RBCs to spread DNA vaccine delivery.
- RBC-hitchhiking DNA nanovaccines preferentially accumulated in the spleen, promoting neoantigen expression by antigen-presenting cells (APCs) and inducing a strong neoantigen-specific T cell response.
- This vaccination regimen prevented tumorigenesis in a personalized manner and slowed down tumor growth in the aggressively growing HCC tumor characterized by an immune "cold" microenvironment.
- Combining RBC-hitchhiking DNA nanovaccines with anti-PD-1 checkpoint blockade led to complete tumor regression (75% and 100% in subcutaneous and orthotopic HCC mouse models, respectively) and prolonged mouse survival.

Please upload your image as a separate file in jpg or png format 550 px wide x 300-600 px high. The text should remain legible.

5/ As part of the EMBO Publications transparent editorial process initiative (see our Editorial at http://embomolmed.embopress.org/content/2/9/329), EMBO Molecular Medicine will publish online a Review Process File (RPF) to accompany accepted manuscripts.
This file will be published in conjunction with your paper and will include the anonymous referee reports, your point-by-point response and all pertinent correspondence relating to the manuscript. Let us know whether you agree with the publication of the RPF and as here, if you want to remove or not any figures from it prior to publication.

Please note that the Authors checklist will be published at the end of the RPF.

I look forward to receiving your revised manuscript.

Yours sincerely,

Lise Roth

Lise Roth, PhD
Senior Editor
EMBO Molecular Medicine

The system will prompt you to fill in your funding and payment information. This will allow Wiley to send you a quote for the article processing charge (APC) in case of acceptance. This quote takes into account any reduction or fee waivers that you may be eligible for. Authors do not need to pay any fees before their manuscript is accepted and transferred to our publisher.

***** Reviewer's comments *****

Referee #1 (Comments on Novelty/Model System for Author):

Reviewer can not understand how OVA257-264 DNA veccine plasmid is constructed. in Construction of pDNA encoding neoantigen or OVA257-264, authors said that "Nucleotide: GGCCGTATTGGCCCCGCCACCTGTGAGCGGGAGCATCATCAACTTCGAGAAGCTGTAAGGCCAAACAGGCC is synthesized." could author specify how the OVA antigen is translated? the translated peptide would be "AVLAPPPVSGSIINFEKL*". This peptide could not be processed by DCs and therefore, can not be presented. Please explain.

Referee #1 (Remarks for Author):

scientifically, the manuscript didnt explained how the experiments worked (See).
I would suggest the authors to have professionals to edit the language of the manuscript. line26-27, page 11: "due to their readily diffuse", should be ready diffuse.

Referee #2 (Comments on Novelty/Model System for Author):

many complementary innovative techniques for the immunomonitoring of DNA vaccines
There is a medical need for this kind of products

Referee #2 (Remarks for Author):

The authors have satisfactorily answered to my previous concerns

Referee #3 (Remarks for Author):

The authors have addressed most, if not all, comments raised and this reviewer has no more concerns.

Dear Dr. Roth,

Thanks very much for your time and efforts on our manuscript entitled "Spleen targeted neoantigen DNA vaccine for personalized immunotherapy of hepatocellular carcinoma" (Manuscript reference number: EMM-2022-16836-V2). We also sincerely appreciate your and the reviewers' very constructive and insightful comments on our manuscript. All of the comments arisen by the editor and reviewers have been carefully addressed point by point as follows. The manuscript has also been carefully revised according to the editor's and reviewers' suggestions. The revised parts were marked by red in the revised manuscript.

Sincerely yours,

Xiaolong Liu, Ph.D., Prof.,

Mengchao Hepatobiliary Hospital of Fujian Medical University

***** Editor's comments *****

1. Referees' comments: please address the remaining minor concerns/questions from referee #1 in writing.

Response: Thanks very much. We have addressed the remaining minor concerns from referee #1, which are listed in the following responses.

2. Main manuscript text:

- Please address the queries from our data editors in the related "Data edited MS file", remove the red text and only keep in track changes any new modification.

Response: Thanks very much. We have addressed the queries from your data editors in the related "Data edited MS file", removed the red text, and only kept track of any new changes in the revised manuscript.

- As mentioned also by referee #1, the text needs to be carefully proof-read for grammar and typos.

Response: Thanks very much. The manuscript text has been meticulously read to

avoid spelling or grammatical errors as much as possible.

- *Materials and methods:*

o Cell lines: please indicate whether the cells were tested for mycoplasma contamination and authenticated. Please adjust the checklist accordingly.

Response: Thanks very much. We have added the corresponding information in "page 23, line 14-20" as follows: "These cells were cultured and maintained in DMEM (containing 10% fetal bovine serum (FBS) and 100 IU/mL penicillin/streptomycin, Gibco, UA), and their supernatants were tested with the TransDetecte PCR Mycoplasma Detection Kit (TransGen Biotech, Beijing, China) to confirm the absence of mycoplasma infection. The Hepa1-6 cells, serving as the primary focus of this study, underwent additional authentication through multiplex PCR amplification of eighteen short tandem repeat (STR) loci. The authentication report, provided by Genetic Testing Biotechnology Co., Ltd. (Suzhou, China), confirmed a 96.00% match to ExPASy cell line Hepa1-6."

o Antibodies: please provide dilutions/concentrations used

Response: Thanks very much. We have added the corresponding information in the revised manuscript.

o Data Availability: please note that the data must be public before acceptance of the manuscript. Please also check that the link you provided is correct. Kindly remove the sentence: "Expanded View for this article is available online."

Response: Thanks very much. We have reconfirmed the accuracy of the provided link information. Meanwhile, the sentence: "Expanded View for this article is available online." has been removed in the revised manuscript.

- *Acknowledgements: The funding information should match the information provided in the submission system: Funding 2022-S-rc1 is currently missing from the submission system.*

Response: Thanks very much. The information of funding 2022-S-rc1 has been added in the submission system.

- Please remove "correspondence information" from p. 34

Response: Thanks very much. This information has been removed in the revised manuscript.

- The "Captions" heading should be changed to "Figure Legends"

Response: Thanks very much. The "Captions" heading has been changed to "Figure Legends" in the revised manuscript.

3. Figures and Appendix:

- We note that you currently have 2 EV figures, and 32 Appendix figures. Please note that you have the possibility to make some of the Appendix figures EV figures, expendable online (up to 5 EV figures).

Response: Thanks very much. Appendix Fig S1 is now presented as Fig EV1, while Appendix Fig S19 and Fig S20 have been re-arranged and are now shown as Fig EV2. Additionally, Appendix Fig S26 to S28 have been moved and are now included as Fig EV5.

- Appendix figure S24 is currently not referenced in the manuscript text, please adjust accordingly.

Response: Thanks very much. Appendix figure S24 (Appendix Fig S21 in the revised manuscript) has been correctly referenced in the revised manuscript. "Interestingly, the most common negative immune checkpoint PD-L1 was found to be obviously upregulated in tumor tissues of RBC-Nanovaccines-treated mice according to immunofluorescence (IF) and immunohistochemistry (IHC) analysis (Appendix Fig S21)." (page 16, line 6-9)

- If images are reused in different figures, it must be stated in the figure legends. We

noted possible re-use of images in figure 6H and Appendix Figure S23
- Please check the legend of Appendix Figure S25: it references Figure 5F, should it be 6F?

Response: We sincerely apologize for mistakenly reversing the order of Figure 5 and Figure 6 in the submission system and combined PDF version. Specifically, Appendix Figure S23 corresponds to Figure 5H, and Appendix Figure S25 corresponds to Figure 5F. We have corrected them in the revised manuscript. The re-use of images in Figure 5H now has been clearly indicated in the figure legend of Appendix Figure S20 (originally Appendix Figure S23 as you mentioned) in the revised manuscript.

- We would also encourage you to include the source data for figure panels that show essential data. Numerical data should be provided as individual .xls or .csv files (including a tab describing the data). For blots or microscopy, uncropped images should be submitted (using a zip archive if multiple images need to be supplied for one panel). Additional information on source data and instruction on how to label the files are available at https://www.embopress.org/page/journal/17574684/authorguide#sourcedata.

Response: Thanks very much. Now, we have uploaded the source data for figure panels in the submission system.

4. Thank you for providing a synopsis text and image. I slightly edited the text to fit with our style and format, please let me know if you agree with the following or amend as you see fit:

To address the limited efficacy of current personalized cancer vaccines and improve hepatocellular carcinoma (HCC) immunotherapy, a red blood cell (RBC) hitchhiking strategy was developed for targeted delivery of a neoantigen DNA vaccine to the spleen.

- Polymer-lipid nanoparticles (NPs) were designed to encapsulate neoantigen-carrying plasmid DNA (pDNA). These nanovaccines were then attached to pre-isolated RBCs to spread DNA vaccine delivery.

- RBC-hitchhiking DNA nanovaccines preferentially accumulated in the spleen, promoting neoantigen expression by antigen-presenting cells (APCs) and inducing a strong neoantigen-specific T cell response.
- This vaccination regimen prevented tumorigenesis in a personalized manner and slowed down tumor growth in the aggressively growing HCC tumor characterized by an immune "cold" microenvironment.
- Combining RBC-hitchhiking DNA nanovaccines with anti-PD-1 checkpoint blockade led to complete tumor regression (75% and 100% in subcutaneous and orthotopic HCC mouse models, respectively) and prolonged mouse survival.

Response: Thanks very much. We fully agree with the modifications mentioned above.

Please upload your image as a separate file in jpg or png format 550 px wide x 300-600 px high. The text should remain legible.

Response: Thanks very much. We have now uploaded the synopsis image as a separate file in the submission system.

5. As part of the EMBO Publications transparent editorial process initiative (see our Editorial at http://embomolmed.embopress.org/content/2/9/329), EMBO Molecular Medicine will publish online a Review Process File (RPF) to accompany accepted manuscripts.
This file will be published in conjunction with your paper and will include the anonymous referee reports, your point-by-point response and all pertinent correspondence relating to the manuscript. Let us know whether you agree with the publication of the RPF and as here, if you want to remove or not any figures from it prior to publication.
Please note that the Authors checklist will be published at the end of the RPF.

Response: Thanks very much. We agree with the publication of the RPF along with our paper.

***** Reviewer's comments *****

Referee #1 (Comments on Novelty/Model System for Author):

Reviewer can not understand how OVA257-264 DNA veccine plasmid is constructed. in Construction of pDNA encoding neoantigen or OVA257-264, authors said that "Nucleotide: GGCCGTATTGGCCCCGCCACCTGTGAGCGGGAGCATCATCAACTTCGAGAAGCTGTAAGGCCAAACAGGCC is synthesized." could author specify how the OVA antigen is translated? the translated peptide would be "AVLAPPPVSGSIINFEKL". This peptide could not be processed by DCs and therefore, can not be presented. Please explain.*

Response: Thanks very much. We constructed the OVA257-264 PresentER plasmid (GGCCGTATTGGCCCCGCCACCTGTGAGCGGGAGCATCATCAACTTCGAGAAGCTG(amino acid sequence: SIINFEKL)TAAGGCCAAACAGGCC) following the "Protocol for cloning MHC ligand minigenes into PresentER vector" process from Ron S Gejman and David A Scheinberg group (Figure Q1 for reviewer only). The PresentER OVA$_{257-264}$ antigen minigene consists of an endoplasmic reticulum (ER) signal sequence derived from a Human Betaretrovirus (GenBank: AFZ15783.1), followed by a short peptide/epitope like OVA antigen. Expression and presentation of the peptide on MHC-I do not require proteasomal degradation or peptide processing, allowing for precise definition of the epitope displayed to the immune system. Consequently, the sequence ultimately expressed and presented on the cell surface was the SIINFEKL epitope peptide.

a

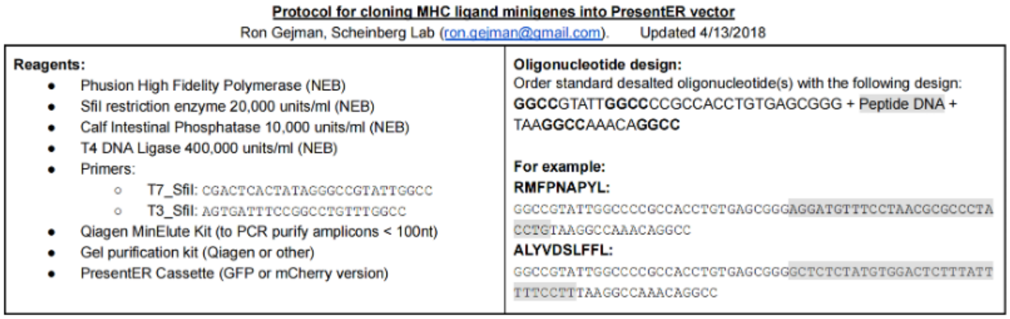

Protocol for cloning MHC ligand minigenes into PresentER vector

Ron Gejman, Scheinberg Lab (ron.gejman@gmail.com). Updated 4/13/2018

Reagents:

- Phusion High Fidelity Polymerase (NEB)
- SfiI restriction enzyme 20,000 units/ml (NEB)
- Calf Intestinal Phosphatase 10,000 units/ml (NEB)
- T4 DNA Ligase 400,000 units/ml (NEB)
- Primers:
  - T7_SfiI: CGACTCACTATAGGGCCGTATTGGCC
  - T3_SfiI: AGTGATTTCCGGCCTGTTTGGCC
- Qiagen MinElute Kit (to PCR purify amplicons < 100nt)
- Gel purification kit (Qiagen or other)
- PresentER Cassette (GFP or mCherry version)

Oligonucleotide design:

Order standard desalted oligonucleotide(s) with the following design:

GGCCGTATT**GGCC**CCGCCACCTGTGAGCGGG + Peptide DNA + TAA**GGCC**AAACA**GGCC**

For example:

RMFPNAPYL:

GGCCGTATTGGCCCCGCCACCTGTGAGCGGGAGGATGTTTCCTAACGCGCCCTACCTGTAAGGCCAAACAGGCC

ALYVDSLFFL:

GGCCGTATTGGCCCCGCCACCTGTGAGCGGGGCTCTCTATGTGGACTCTTTATTTTTCCTTTAAGGCCAAACAGGCC

b

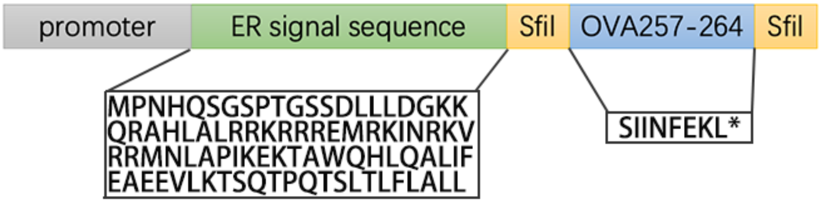

Figure Q1. (a) The information of the PresentER vector downloaded from Addgene, which was provided by Ron S Gejman and David A Scheinberg group. (b) The expressed protein sequence of ER signal plus $OVA_{257\text{-}264}$.

Referee #1 (Remarks for Author):

scientifically, the manuscript didnt explained how the experiments worked (See). I would suggest the authors to have professionals to edit the language of the manuscript. line26-27, page 11: "due to their readily diffuse", should be ready diffuse.

Response: Thanks for your careful checks. Based on your comments, we have polished our manuscript.

25th Jul 2023

Dear Dr. Liu,

Thank you for submitting your revised files. I am pleased to inform you that your manuscript is now accepted for publication in EMBO Molecular Medicine!
Before I can send your manuscript to our publisher, please address the remaining minor editorial issues:

1. Please address the queries from our data editors in the related "Data edited MS file". I copied these comments here for your convenience:
o Figure 1I: Is it meant SEM (standard error of the mean)? In the "Data information" section below, data in I are defined as mean +/- SD, which is a contradiction, please check.
o Figure 5: Please define the statistical test in G.
2. In the Appendix Figure S20, please indicate in the legend: "The pictures from the lower row are re-used from figure 5H".

Additionally, if you used an image data base for scientific iconography (e.g., BioRender), please let us know if you have a license that allows for publication in an academic journal and indicate its use in the manuscript.

Once these modifications are made, please accept all changes, and remove the red text. You may send me the final files via email and I'll upload them in the submission system.

Please read below for additional IMPORTANT information regarding your article, its publication and the production process.

Congratulations on your interesting work!

With kind regards,

Lise Roth

Lise Roth, PhD
Senior Editor
EMBO Molecular Medicine

*** *** *** IMPORTANT INFORMATION *** *** ***

SPEED OF PUBLICATION
The journal aims for rapid publication of papers, using using the advance online publication "Early View" to expedite the process: A properly copy-edited and formatted version will be published as "Early View" after the proofs have been corrected. Please help the Editors and publisher avoid delays by providing e-mail address(es), telephone and fax numbers at which author(s) can be contacted.

Should you be planning a Press Release on your article, please get in contact with embomolmed@wiley.com as early as possible, in order to coordinate publication and release dates.

LICENSE AND PAYMENT:

All articles published in EMBO Molecular Medicine are fully open access: immediately and freely available to read, download and share.

EMBO Molecular Medicine charges an article processing charge (APC) to cover the publication costs. You, as the corresponding author for this manuscript, should have already received a quote with the article processing fee separately. Please let us know in case this quote has not been received.

Once your article is at Wiley for editorial production you will receive an email from Wiley's Author Services system, which will ask you to log in and will present you with the publication license form for completion. Within the same system the publication fee

can be paid by credit card, an invoice, pro forma invoice or purchase order can be requested.

Payment of the publication charge and the signed Open Access Agreement form must be received before the article can be published online.

PROOFS

You will receive the proofs by e-mail approximately 2 weeks after all relevant files have been sent o our Production Office. Please return them within 48 hours and if there should be any problems, please contact the production office at embopressproduction@wiley.com.

Please inform us if there is likely to be any difficulty in reaching you at the above address at that time. Failure to meet our deadlines may result in a delay of publication.

All further communications concerning your paper proofs should quote reference number EMM-2022-16836-V3 and be directed to the production office at embopressproduction@wiley.com.

Dear Dr. Roth,

Thank you for accepting our manuscript for publication in EMBO Molecular Medicine (Manuscript reference number: EMM-2022-16836-V3). We also sincerely appreciate your and other data editors' very constructive on our manuscript. All of editorial issues have been carefully addressed point by point as follows. The revised Manuscript Text and Appendix Figures are attached in the email, please review them at your convenience.

Sincerely yours,

Xiaolong Liu, Ph.D., Prof.,

Mengchao Hepatobiliary Hospital of Fujian Medical University

***** Editor's comments *****

1. Please address the queries from our data editors in the related "Data edited MS file". I copied these comments here for your convenience:

Response: Thanks very much. We have addressed the queries from your data editors in the related "Data edited MS file".

o Figure 1I: Is it meant SEM (standard error of the mean)? In the "Data information" section below, data in I are defined as mean +/- SD, which is a contradiction, please check.

Response: Thanks very much. We have carefully examined the raw data and confirmed that Figure 1I represents mean +/- SD. To avoid ambiguity, we have modified the presentation format of Figure 1I. The revised Figure 1 has been attached to this email. Kindly assist me in uploading it to the submission system to replace the original one.

o Figure 5: Please define the statistical test in G.

Response: Thanks very much. The Gehan-Breslow-Wilcoxon test was employed for (G), and this information has been added to the legend of the Figure 5.

2. In the Appendix Figure S20, please indicate in the legend: "The pictures from the lower row are re-used from figure 5H".

Response: Thanks very much. These words have been added in the legend of Figure S20.

Additionally, if you used an image data base for scientific iconography (e.g., BioRender), please let us know if you have a license that allows for publication in an academic journal and indicate its use in the manuscript.

Response: Thanks very much. We confirm that all the images used in the article are original and have not been sourced from previously published articles or scientific iconography platforms like BioRender.

EMBO Press Author Checklist

Corresponding Author Name: Xiaolong Liu	
Journal Submitted to: EMBO Molecular Medicine	
Manuscript Number: EMM-2022-16836-V2	

USEFUL LINKS FOR COMPLETING THIS FORM
The EMBO Journal - Author Guidelines
EMBO Reports - Author Guidelines
Molecular Systems Biology - Author Guidelines
EMBO Molecular Medicine - Author Guidelines

Reporting Checklist for Life Science Articles (updated January
This checklist is adapted from Materials Design Analysis Reporting (MDAR) Checklist for Authors. MDAR establishes a minimum set of requirements in transparent reporting in the life sciences (see Statement of Task: 10.31222/osf.io/9sm4x). Please follow the journal's guidelines in preparing your manuscript.
Please note that a copy of this checklist will be published alongside your article.

Abridged guidelines for figures
1. Data
The data shown in figures should satisfy the following conditions:
- the data were obtained and processed according to the field's best practice and are presented to reflect the results of the experiments in an accurate and unbiased manner.
- ideally, figure panels should include only measurements that are directly comparable to each other and obtained with the same assay.
- plots include clearly labeled error bars for independent experiments and sample sizes. Unless justified, error bars should not be shown for technical
- if n<5, the individual data points from each experiment should be plotted. Any statistical test employed should be justified.
- Source Data should be included to report the data underlying figures according to the guidelines set out in the authorship guidelines on Data

2. Captions
Each figure caption should contain the following information, for each panel where they are relevant:
- a specification of the experimental system investigated (eg cell line, species name).
- the assay(s) and method(s) used to carry out the reported observations and measurements.
- an explicit mention of the biological and chemical entity(ies) that are being measured.
- an explicit mention of the biological and chemical entity(ies) that are altered/varied/perturbed in a controlled manner.
- the exact sample size (n) for each experimental group/condition, given as a number, not a range;
- a description of the sample collection allowing the reader to understand whether the samples represent technical or biological replicates (including how many animals, litters, cultures, etc.).
- a statement of how many times the experiment shown was independently replicated in the laboratory.
- definitions of statistical methods and measures:
  - common tests, such as t-test (please specify whether paired vs. unpaired), simple χ2 tests, Wilcoxon and Mann-Whitney tests, can be unambiguously identified by name only, but more complex techniques should be described in the methods section;
  - are tests one-sided or two-sided?
  - are there adjustments for multiple comparisons?
  - exact statistical test results, e.g., P values = x but not P values < x;
  - definition of 'center values' as median or average;
  - definition of error bars as s.d. or s.e.m.

Please complete ALL of the questions below.
Select "Not Applicable" only when the requested information is not relevant for your study.

Materials

Newly Created Materials	Information included in the manuscript?	In which section is the information available? (Reagents and Tools Table, Materials and Methods, Figures, Data Availability Section)
New materials and reagents need to be available; do any restrictions apply?	Not Applicable	

Antibodies	Information included in the manuscript?	In which section is the information available? (Reagents and Tools Table, Materials and Methods, Figures, Data Availability Section)
For **antibodies** provide the following information:<br>- Commercial antibodies: RRID (if possible) or supplier name, catalogue number and or/clone number<br>- Non-commercial: RRID or citation	Yes	Materials and Methods

DNA and RNA sequences	Information included in the manuscript?	In which section is the information available? (Reagents and Tools Table, Materials and Methods, Figures, Data Availability Section)
**Short novel DNA or RNA including primers, probes**: provide the sequences.	Yes	Materials and Methods

Cell materials	Information included in the manuscript?	In which section is the information available? (Reagents and Tools Table, Materials and Methods, Figures, Data Availability Section)
**Cell lines:** Provide species information, strain. Provide accession number in repository **OR** supplier name, catalog number, clone number, and/**OR** RRID.	Yes	Materials and Methods
**Primary cultures:** Provide species, strain, sex of origin, genetic modification status.	Yes	Materials and Methods
Report if the cell lines were recently **authenticated** (e.g., by STR profiling) and tested for mycoplasma contamination.	Yes	Materials and Methods

Experimental animals	Information included in the manuscript?	In which section is the information available? (Reagents and Tools Table, Materials and Methods, Figures, Data Availability Section)
**Laboratory animals or Model organisms:** Provide species, strain, sex, age, genetic modification status. Provide accession number in repository **OR** supplier name, catalog number, clone number, **OR** RRID.	Yes	Materials and Methods
**Animal observed in or captured from the field:** Provide species, sex, and age where possible.	Not Applicable	
Please detail **housing and husbandry conditions**.	Yes	Materials and Methods

Plants and microbes	Information included in the manuscript?	In which section is the information available? (Reagents and Tools Table, Materials and Methods, Figures, Data Availability Section)
**Plants:** provide species and strain, ecotype and cultivar where relevant, unique accession number if available, and source (including location for collected wild specimens).	Not Applicable	
**Microbes:** provide species and strain, unique accession number if available, and source.	Not Applicable	

Human research participants	Information included in the manuscript?	In which section is the information available? (Reagents and Tools Table, Materials and Methods, Figures, Data Availability Section)
If collected and within the bounds of privacy constraints report on age, sex and gender or ethnicity for all study participants.	Not Applicable	

Core facilities	Information included in the manuscript?	In which section is the information available? (Reagents and Tools Table, Materials and Methods, Figures, Data Availability Section)
If your work benefited from core facilities, was their service mentioned in the acknowledgments section?	Not Applicable	

Design

Study protocol	Information included in the manuscript?	In which section is the information available? (Reagents and Tools Table, Materials and Methods, Figures, Data Availability Section)
If study protocol has been **pre-registered, provide DOI in the manuscript**. For clinical trials, provide the trial registration number **OR** cite DOI.	Not Applicable	
Report the **clinical trial registration number** (at ClinicalTrials.gov or equivalent), where applicable.	Not Applicable	

Laboratory protocol	Information included in the manuscript?	In which section is the information available? (Reagents and Tools Table, Materials and Methods, Figures, Data Availability Section)
Provide DOI OR other citation details if **external detailed step-by-step protocols** are available.	Not Applicable	

Experimental study design and statistics	Information included in the manuscript?	In which section is the information available? (Reagents and Tools Table, Materials and Methods, Figures, Data Availability Section)
Include a statement about **sample size** estimate even if no statistical methods were used.	Yes	Figure legends
Were any steps taken to minimize the effects of subjective bias when allocating animals/samples to treatment (e.g. **randomization procedure**)? If yes, have they been described?	Yes	Materials and Methods
Include a statement about **blinding** even if no blinding was done.	Yes	Materials and Methods
Describe **inclusion/exclusion criteria** if samples or animals were excluded from the analysis. Were the criteria pre-established?<br><br>If sample or data points were omitted from analysis, report if this was due to attrition or intentional exclusion and provide justification.	Yes	Materials and Methods
For every figure, are **statistical tests** justified as appropriate? Do the data meet the assumptions of the tests (e.g., normal distribution)? Describe any methods used to assess it. Is there an estimate of variation within each group of data? Is the variance similar between the groups that are being statistically compared?	Yes	Materials and Methods

Sample definition and in-laboratory replication	Information included in the manuscript?	In which section is the information available? (Reagents and Tools Table, Materials and Methods, Figures, Data Availability Section)
In the figure legends: state number of times the experiment was **replicated** in laboratory.	Yes	Figure legends
In the figure legends: define whether data describe **technical or biological replicates**.	Yes	Figure legends

Ethics

Ethics	Information included in the manuscript?	In which section is the information available? (Reagents and Tools Table, Materials and Methods, Figures, Data Availability Section)
Studies involving **human participants**: State details of **authority granting ethics approval** (IRB or equivalent committee(s), provide reference number for approval.	Not Applicable	
Studies involving **human participants**: Include a statement confirming that **informed consent** was obtained from all subjects and that the experiments conformed to the principles set out in the WMA Declaration of Helsinki and the Department of Health and Human Services Belmont Report.	Not Applicable	
Studies involving **human participants:** For publication of **patient photos**, include a statement confirming that consent to publish was obtained.	Not Applicable	
Studies involving experimental **animals**: State details of **authority granting ethics approval** (IRB or equivalent committee(s), provide reference number for approval. Include a statement of compliance with ethical regulations.	Yes	Materials and Methods
Studies involving **specimen and field samples:** State if relevant **permits** obtained, provide details of authority approving study; if none were required, explain why.	Not Applicable	

Dual Use Research of Concern (DURC)	Information included in the manuscript?	In which section is the information available? (Reagents and Tools Table, Materials and Methods, Figures, Data Availability Section)
Could your study fall under dual use research restrictions? Please check biosecurity documents and list of **select agents and toxins** (CDC): https://www.selectagents.gov/sat/list.htm	Not Applicable	
If you used a select agent, is the security level of the lab appropriate and reported in the manuscript?	Not Applicable	
If a study is subject to dual use research of concern regulations, is the name of the **authority granting approval and reference number** for the regulatory approval provided in the manuscript?	Not Applicable	

Reporting

The MDAR framework recommends adoption of discipline-specific guidelines, established and endorsed through community initiatives. Journals have their own policy about requiring specific guidelines and recommendations to complement MDAR.

Adherence to community standards	Information included in the manuscript?	In which section is the information available? (Reagents and Tools Table, Materials and Methods, Figures, Data Availability Section)
State if relevant guidelines or checklists (e.g., **ICMJE, MIBBI, ARRIVE, PRISMA**) have been followed or provided.	Not Applicable	
For **tumor marker prognostic studies,** we recommend that you follow the **REMARK** reporting guidelines (see link list at top right). See author guidelines, under 'Reporting Guidelines'. Please confirm you have followed these guidelines.	Not Applicable	
For **phase II and III randomized controlled trials**, please refer to the **CONSORT** flow diagram (see link list at top right) and submit the CONSORT checklist (see link list at top right) with your submission. See author guidelines, under 'Reporting Guidelines'. Please confirm you have submitted this list.	Not Applicable	

Data Availability

Data availability	Information included in the manuscript?	In which section is the information available? (Reagents and Tools Table, Materials and Methods, Figures, Data Availability Section)
Have **primary datasets** been deposited according to the journal's guidelines (see 'Data Deposition' section) and the respective accession numbers provided in the Data Availability Section?	Yes	Data Availability
Were **human clinical and genomic datasets** deposited in a public access-controlled repository in accordance to ethical obligations to the patients and to the applicable consent agreement?	Not Applicable	
Are **computational models** that are central and integral to a study available without restrictions in a machine-readable form? Were the relevant accession numbers or links provided?	Not Applicable	
If publicly available data were reused, provide the respective **data citations in the reference list**.	Not Applicable	
